# Supplementary material for: Assessing household financial burdens for preprimary education and associated socioeconomic inequalities: a case study in China
Source: BMJ Paediatr Open. 2023 Aug 10;7(1):e001971. doi: 10.1136/bmjpo-2023-001971 (PMC10423832; doi:10.1136/bmjpo-2023-001971)
Supplement: Supplementary data [file bmjpo-2023-001971supp001.pdf]

Appendix

Chapter 1 Sampling Process of the China Household Finance Survey (CHFS)
Chapter 2 Constructing variables on socioecnomic charisteristics
Chapter 3 Testing the statistical significance of inequalities in PPE spending and HBPPE using Cumming and Finch's "rule of eye"
Chapter 4. Calculating provincial-level disparities in PPE spending and % of households with HBPPE
Chapter 5. Sensitivity tests using proportion of households spending on PPE as outcome variable and using continuous household net wealth as a predictor
Figure A1. Government education spending on preschool education
Figure A2. Preschool enrollment and gross enrollment rate
Figure A3. Geographical distribution of the sampled counties
Figure A4. Level and inequalities of PPE spending per child excluding out-school expenditure
Figure A5. Level of % of households with HBPPE (threshold=10%) across 29 provinces
Figure A6. Level of % of households with HBPPE (threshold=20%) across 29 provinces
Figure A7. Level and inequalities of % of households with HBPPE (thresehold=10%) by type of kindergarten, maternal education, household net wealth quintile, and current residential area
Figure A8. Level and inequalities of % of households with HBPPE (thresehold=20%) by type of kindergarten, maternal education, household net wealth quintile, and current residential area
Figure A9. Level and inequalities of % of households with HBPPE (threshold=7%) by excluding out-school spending from PPE spending
Figure A10. Level and inequalities of % of households with HBPPE (threshold=10%) by excluding out-school spending from PPE spending
Figure A11. Level and inequalities of % of households with HBPPE (threshold=20%) by excluding out-school spending from PPE spending
Figure A12. Level of % of household expenditure on PPE across 29 provinces
Figure A13. Level and inequalities of % of household expenditure on PPE by type of kindergarten, maternal education, household net wealth quintile, and current residential area
Figure A14. Level and inequalities of % of household expenditure on PPE by excluding out-school spending from PPE spending

Table A1. Significance testing of inequalities in PPE spending per child using Cumming and Finch's "rule of eye"
Table A2. Level of % of households with HBPPE (threshold=7%) across 29 provinces and weighted mean difference of the mean between the provincial level and the national level
Table A3. Level of % of households with HBPPE (threshold=10%) across 29 provinces and weighted mean difference of the mean between the provincial level and the national level
Table A4. Level of % of households with HBPPE (threshold=20%) across 29 provinces and weighted mean difference of the mean between the provincial level and the national level
Table A5. Significance testing of inequalities in HBPPE (threshold=7%) using Cumming and Finch's "rule of eye"

Wei Y, *et al. BMJ Paediatrics Open* 2023; 7:e001971. doi: 10.1136/bmjpo-2023-001971

Table A6. Significance testing of inequalities in HBPPE (threshold=10%) using Cumming and Finch's "rule of eye"
Table A7. Significance testing of inequalities in HBPPE (threshold=20%) using Cumming and Finch's "rule of eye"
Table A8. Level of % of household expenditure on PPE across 29 provinces and weighted mean difference of the mean between the provincial level and the national level
Table A9. Multilevel linear regression results on factors associated with logged % of household expenditure on PPE
Table A10. Beta regression results on factors associated with % of household expenditure on PPE
Table A11. Multilevel logit regression results on factors associated with household status on HBPPE by setting household net wealth as a continuous variable
Table A12. Multilevel linear regression and Beta regression results on factors associated with % of household expenditure on PPE by setting household net wealth as a continuous variable

Chapter 1 Sampling Process of the China Household Finance Survey (CHFS)

The CHFS survey employs a stratified three-stage probability proportion-to-size random sample design. The primary sampling units include 2,585 counties/provincial cities in China. Tibet, Xinjiang, Hong Kong, Macao, and Chinese Taipei are not included in the primary sampling stage. The second stage of sampling involves selecting residential committees/villages from the counties/cities. The last stage involves selecting households from the residential committees/villages. A household unit is defined as all members of a housing unit related by blood, marriage, or some other legal arrangement; or two or more persons use incomes to make joint expenditures, or a single person living with others but is financially independent.

The CHFS is nationally and provincially representative survey conducted by the Southwestern University of Finance and Economics in China.[1] The CHFS collects rich information on household assets, income, wealth, and consumption. It also collects socioeconomic and demographic information on the sampled households and their members, including residential areas, household members' ages, gender, education levels, marital status, etc. The CHFS data are publicly available and have been widely used in the fields of economics, sociology, and education. By the end of November 2021, more than 35,000 researchers and 3,000 research institutions had registered and applied for the dataset, and the related studies have been published in top peer-reviewed journals.[2] Since 2017, the CHFS survey collaborated with the China Institute for Educational Finance Research and included a module on household spending on young children's care and education. The module includes questions on young children's schooling status, types of schooling, and household spending on various items that are related to preprimary attendance.

The CHFS 2019 covers 29 provinces (excluding Tibet, Xinjiang, Inner Mongolia, Hong Kong, and Macau), 345 cities/districts/counties, 34,643 households, and 107,008 individuals. Our focus was on household spending related to attending kindergarten. In China, kindergarten is usually offered to children 3-6 years old, but children under age 3 and over age 6 may also attend. In this study, we focused on children attending kindergarten, including those under age 3 or over age 6

who still attended kindergarten (18.2%). The 2019 data includes 4,085 young children aged 3-6 years old and 2,797 of these children attended kindergartens, yielding a 68.5% net enrollment rate.[3] The number of children attending kindergarten in all age groups is 3,415, yielding 83.6% of the gross enrollment rate, very close to the governmental estimate (83.4%).[4] Among households in this dataset, 42 (0.18%) didn't report spending information on PPE, and two reported PPE spending exceeding total household expenditures, both of which groups were excluded. The final sample includes 3,371 young children attending kindergartens, from 3,111 households (9% of the total sample), who reported available PPE expenditure data.

References

1. Gan, L., Yin, Z., Jia, N., Xu, S., & Ma, S. (2013). Data you need to know about China, Springer.
2. Survey and Research Center for China Household Finance, at https://chfs.swufe.edu.cn/
3. The net enrolment rate (NER) is the number of students of the age of preprimary education (age 3-6) that are enrolled in that level of education, expressed as a percentage of the total population in that age group.
4. The gross enrollment rate (GER) is the total enrollment in preprimary education, regardless of age, expressed as a percentage of the population in the official age group (age 3-6) corresponding to this level of education. The GER can exceed 100% because of early or late entry and/or grade repetition.

Chapter 2 Constructing variables on socioecnomic charisteristics

Household economic status. In this study, we used per capita household net wealth to measure household economic status. Household net wealth was derived by calculating the difference between a household's assets and its debts. Household total assets include financial assets (e.g., cash, deposit balances, social security, stock/bond value, etc.) and non-financial assets (e.g., agricultural capital, industrial capital, commercial capital, housing, vehicles, and durable goods). Total household debts include financial asset debts, agricultural debts, business debts, housing debts, vehicle debts, other non-financial asset debts, educational debts, credit card debts, medical-care debts, and other debts. In this study, we divided households into five quintiles according to their per capita net wealth. We examined the difference in % of households with HBPPE across the five wealth quintiles.

Maternal schooling. We divided maternal schooling into four categories: primary school or below, middle school, high school, and college or above. About 12% of households in the sample of the study did not report the mothers' education; we replaced the missing data with the predicted values of mother's education by regressing mothers' education on fathers' education (ordered logit regression coefficient = 1.217, $p < 0.001$; correlation coefficient = 0.746) or household heads' education (ordered logit regression coefficient = 1.393, $p < 0.001$; correlation coefficient = 0.632).

Residential area. Because the urban-rural divide is still one of the main structural problems facing the schooling system in China, we also examined the disparities between urban and rural residential areas.

Type of kindergarten. Kindergartens in China can be categorized into public and private types, as discussed above. Households need to pay higher tuition fees to attend private kindergartens than public kindergartens. We assessed the disparities in the percentages of households with HBPPE by kindergarten type.

Chapter 3 Testing the statistical significance of inequalities in PPE spending and HBPPE using Cumming and Finch's "rule of eye"

To test whether inequalities in PPE spending or HBPPE significantly existed between the two sub-groups (e.g. male vs female), we compared the 95% confidence intervals (CIs) of the two estimates using Cumming and Finch's "rule of eye" for comparing two independent means.[1] We calculated three components that were required to implement the "rule of thumb": marginal errors, overlap of the 95% CIs of the two estimates, and proportion overlap. (1) Marginal error refers to the absolute difference between the mean and its lower or higher bound of a 95% CI. (2) An overlap of CIs refers to the difference between one estimate's higher bound and the other estimate's lower bound. (3) Proportion overlap is then calculated as the overlap of the 95% CIs between the two estimates divided by the average marginal errors between the two estimates.

Using Cumming and Finch's "rule of eye", we obtained proportion overlap using Equation PA1 or PA2 as follows, depending on the size of mean estimates for the two sub-groups. For example, when comparing the mean estimates for gender, if the mean estimate for male is lower than that for female, then

$$Proportion\ overlap = \frac{95\%\ CI\ upper\ bound_{male} - 95\%\ CI\ lower\ bound_{female}}{(Margin\ of\ error_{male} + Margin\ of\ error_{female})/2} \qquad \textbf{(PA1)}$$

Or, if the mean estimate for male is higher than that for female, then

$$Proportion\ overlap = \frac{95\%\ CI\ upper\ bound_{female} - 95\%\ CI\ lower\ bound_{male}}{(Margin\ of\ error_{male} + Margin\ of\ error_{female})/2} \qquad \textbf{(PA2)}$$

According to the "rule of eye", when sample sizes of two samples are both at least 10, and the margins of error between the two estimates do not differ by more than a factor of two, a proportion overlap less than 0.5 indicates a significant statistical relationship at the 0.05 level (i.e. $p<0.05$).

References

1. Cumming, G., & Finch, S. (2005). Inference by eye: Confidence intervals and how to read pictures of data. *American Psychologist*, 60(2),170-180.

Chapter 4. Calculating provincial-level disparities in PPE spending and % of households wth HBPPE

Considering the large variation in economic development and geographical situations across the provinces, we further assessed the provincial-level disparities in the level of PPE spending per child and % of households with HBPPE across the 29 provinces. The information will help the central government to identify the provinces with high percentages of households with HBPPE.

Provincial-level inequalities were estimated with the weighted mean from the national mean, which was calculated as the values of the mean difference between each province and the national average, weighted by each province's population share (= $\frac{pop_i \times (r_i - r)}{pop}$), where *pop* is the national population size, pop_i is the population size of province *i*, *r* is the national mean, r_i is the mean of province *i*.). The measures took zero as the reference, with positive values indicating provincial means higher than the national average, and negative values indicating provincial means lower than the national average.

Chapter 5. Sensitivity tests using proportion of households spending on PPE as the outcome variable and using continuous household net wealth as a predictor

We first calculated the provincial-level disparities in the % of household expenditure on PPE across the 29 provinces. On average, households in the Northeastern and middle areas spend higher % of household expenditure on PPE, with Shaanxi (15.4%) and Liaoning (14.8%) showing the highest level. Hebei (6.4%) and Gansu (6.0%) had the lowest level (**Appendix Figure A12 and Table A8**). The pattern across the provinces is similar to the % of households with HBPPE.

In terms of socioeconomic inequalities (**Figure A13**), % of expenditure on PPE was higher among those households sending children to private kindergartens (10.5%) than to public kindergartens (8.1%). Statistically significant differences were not observed across urban and rural households, households with different levels of maternal education, households with different wealth quintiles. The results are consistent with the ones using dichotomous HBPPE. **Figure A14** shows results excluding out-school spending from PPE. The difference between private and public kindergartens increases. Meanwhile, the difference between the highest wealth quintile and the lowest one was statistically sigficant.

Tables A9 and A10 present the regression results using % of household total expenditure on PPE as outcome variable. We used both a multilevel linear regression and a beta regression for estimation. The results are consistent with the main regression results.

Tables A11 and A12 present the regression results using continuous household instead of net wealth quintiles as the predictor. We used a multilevel logit regression, multilevel linear regression and a beta regression for estimation. The results are consistent with the main regression results.

Figure A1. Government education spending on preschool education (Source: China Educational Finance Statistical Yearbook)

Wei Y, *et al. BMJ Paediatrics Open* 2023; 7:e001971. doi: 10.1136/bmjpo-2023-001971

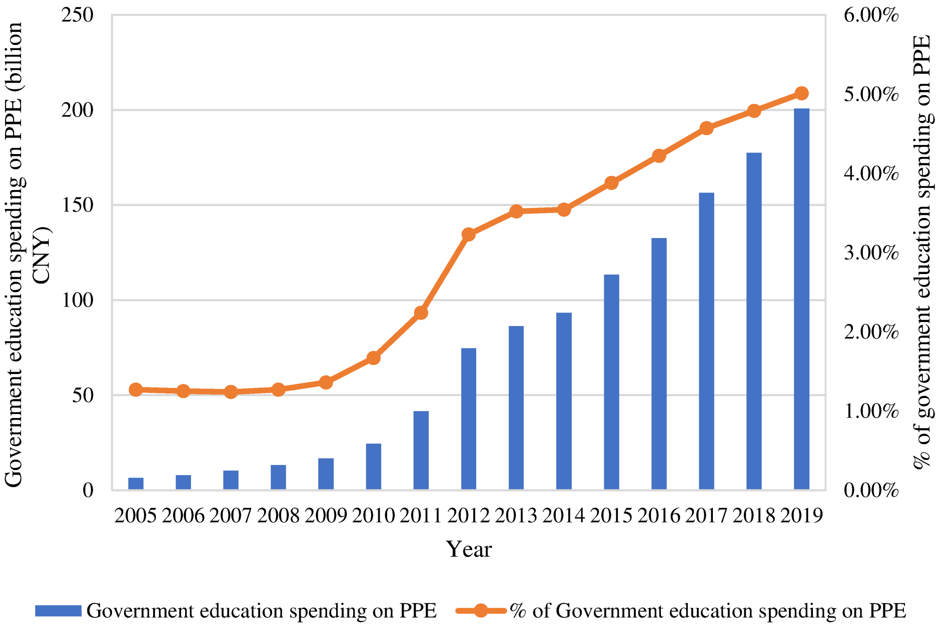

Figure A2. Preschool enrollment and gross enrollment rate (Source: China Education Statistical Yearbook)

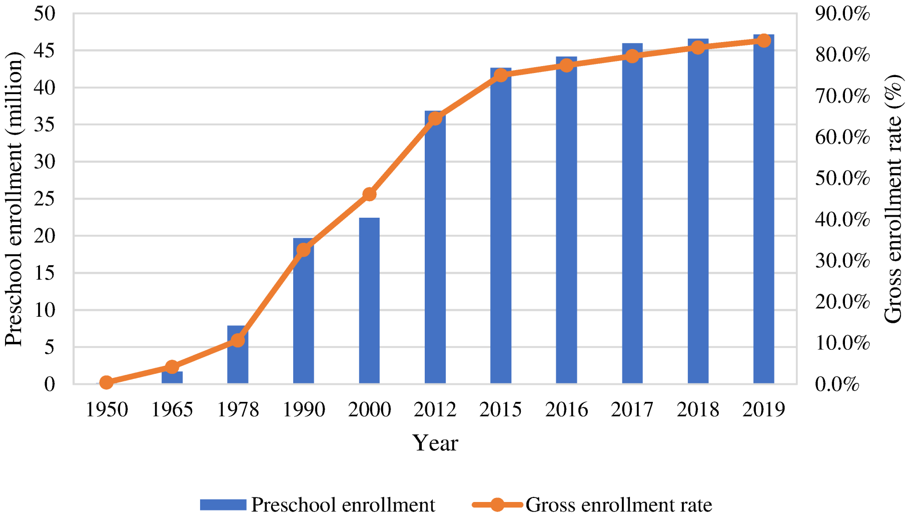

Figure A3. Geographical distribution of the sampled counties

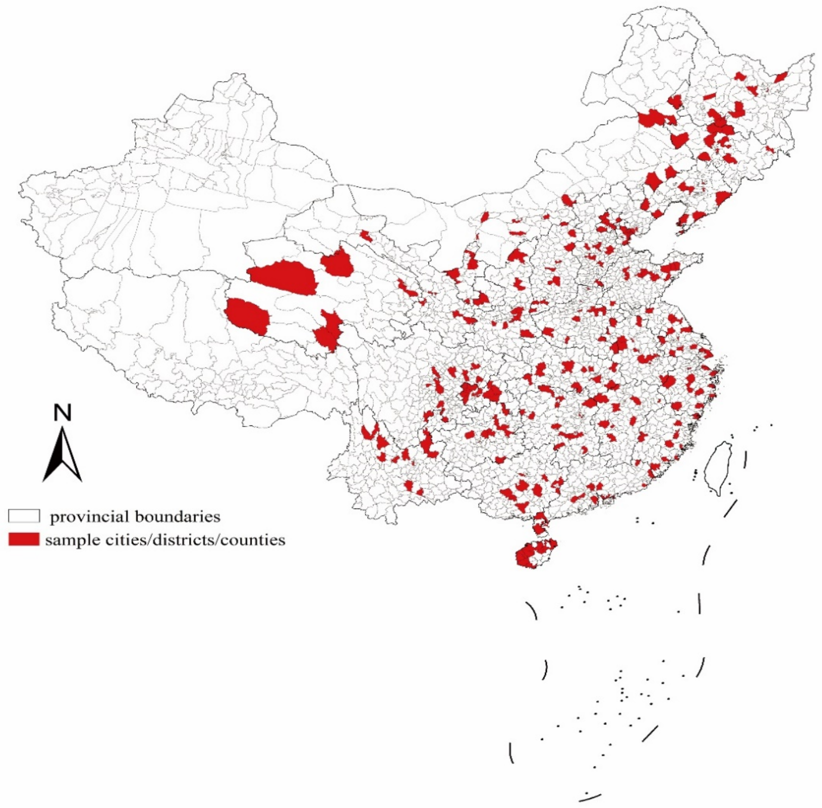
N
provincial boundaries
sample cities/districts/counties

=

Figure A4. Level and inequalities of PPE spending per child excluding out-school expenditure

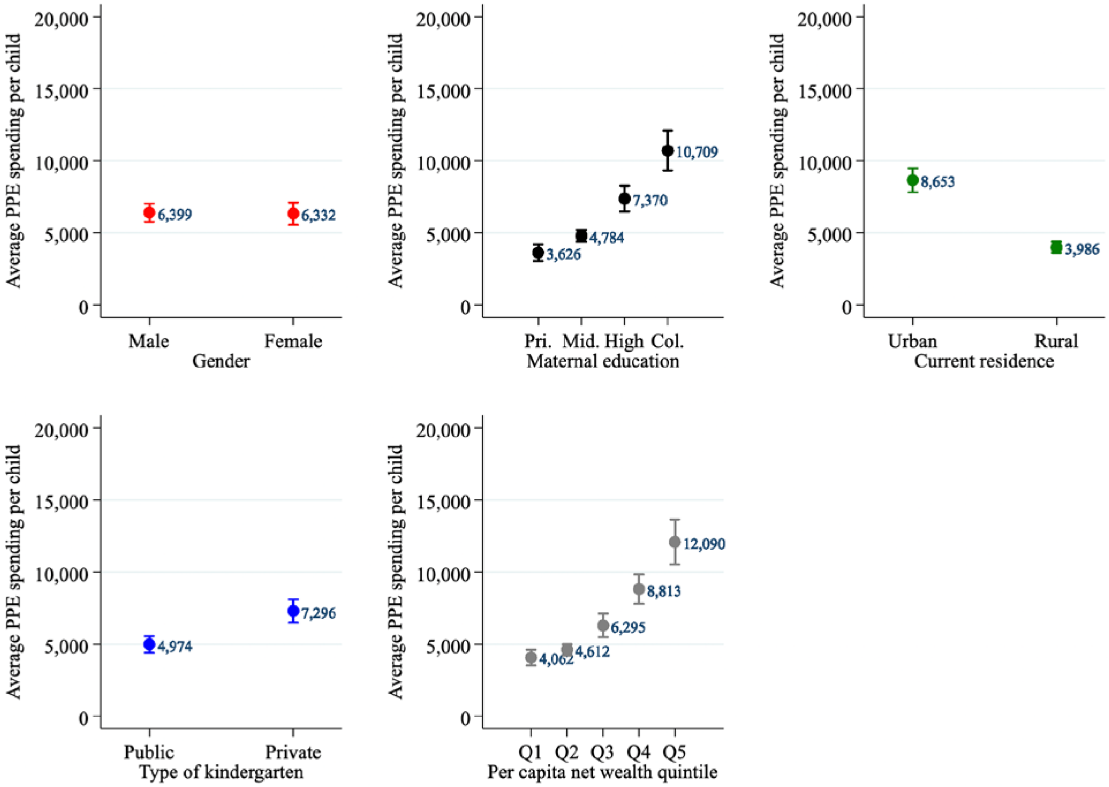

Notes: “Pri.”, “Mid.”, “High”, and “Col.” refers to maternal schooling at the primary and below, middle, high, and college and above levels, respectively.

Figure A5. Level of % of households with HBPPE (threshold=10%) across 29 provinces

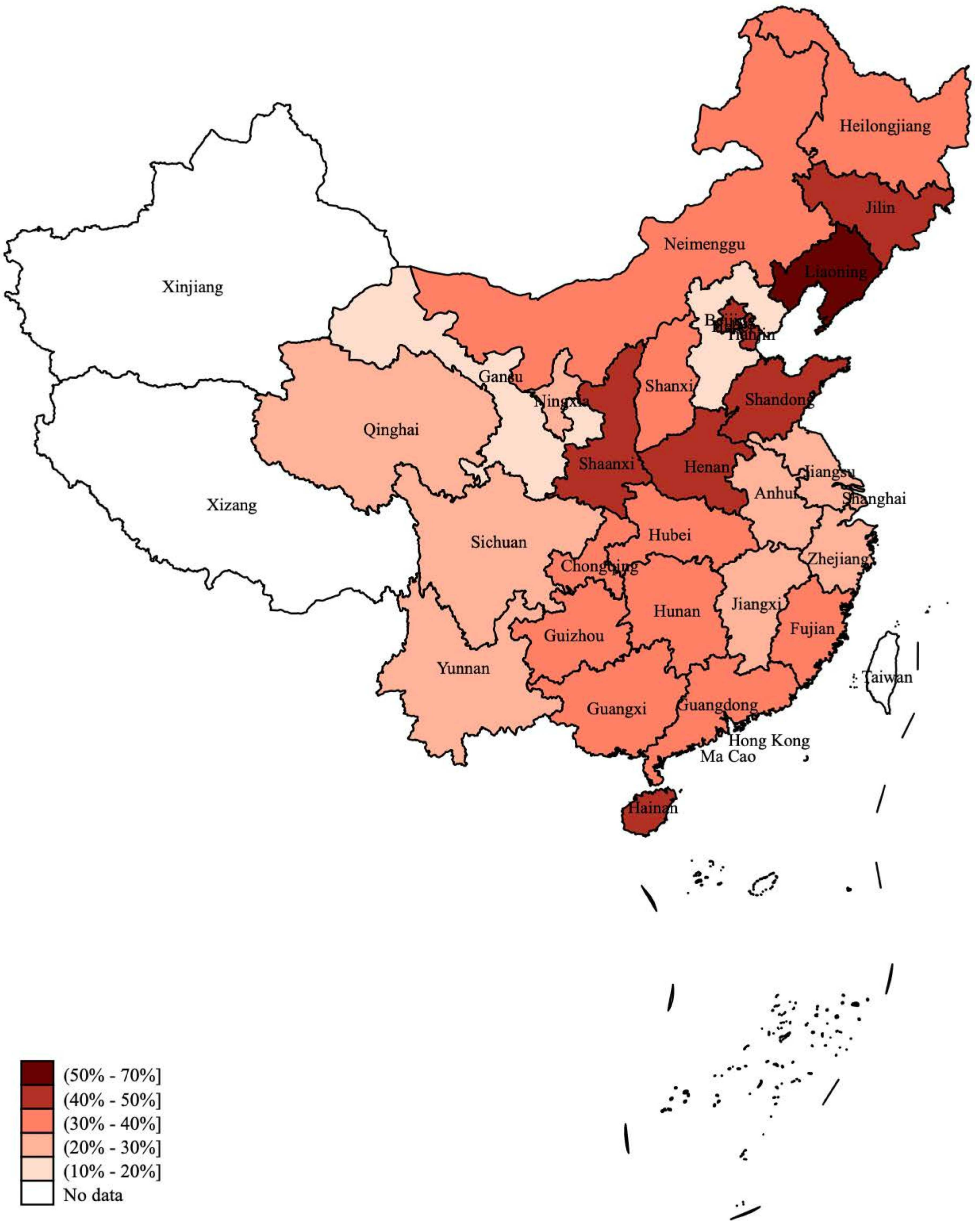

Figure A6. Level of % of households with HBPPE (threshold=20%) across 29 provinces

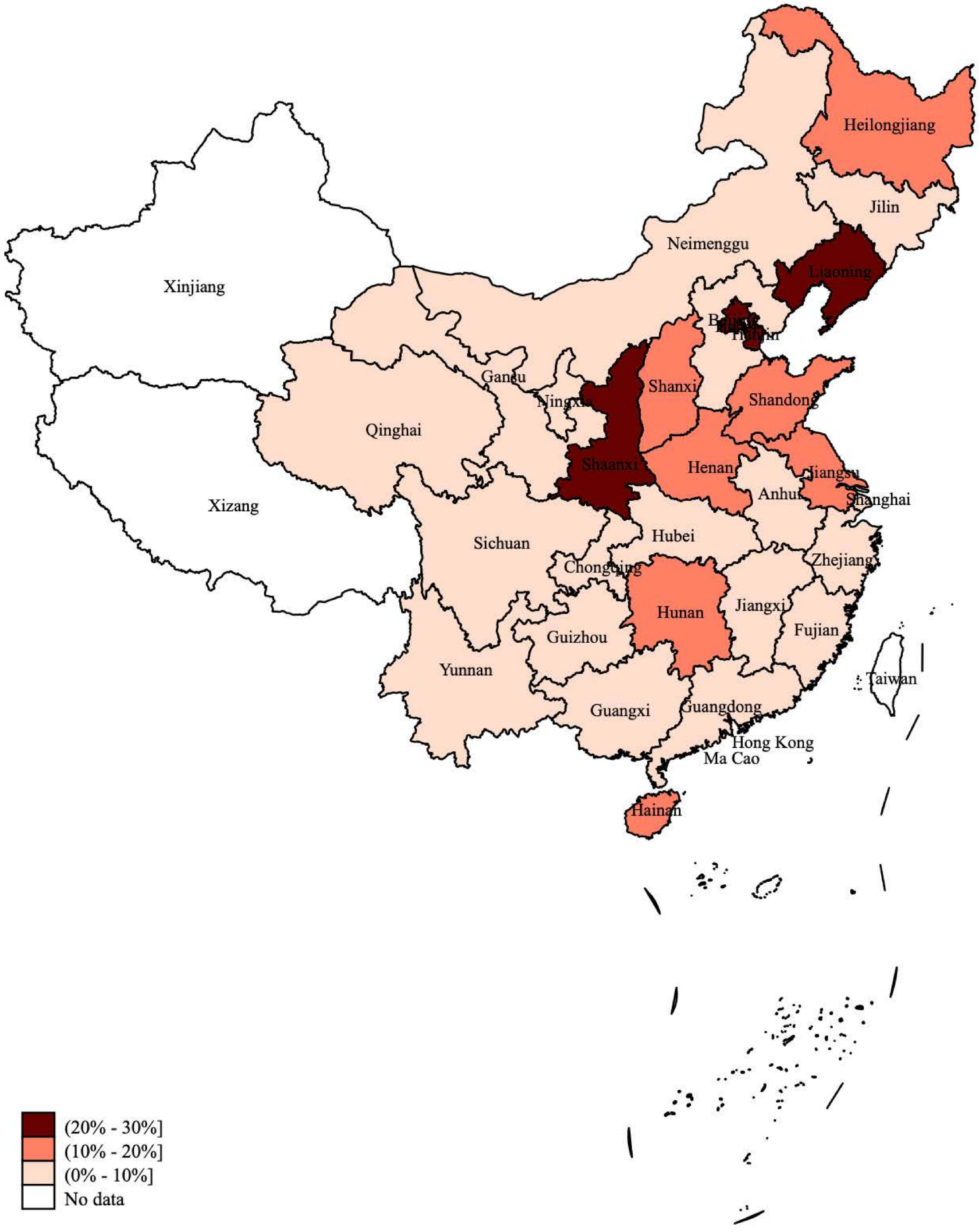

Figure A7. Level and inequalities of % of households with HBPPE (thresehold=10%) by type of kindergarten, maternal education, household net wealth quintile, and current residential area

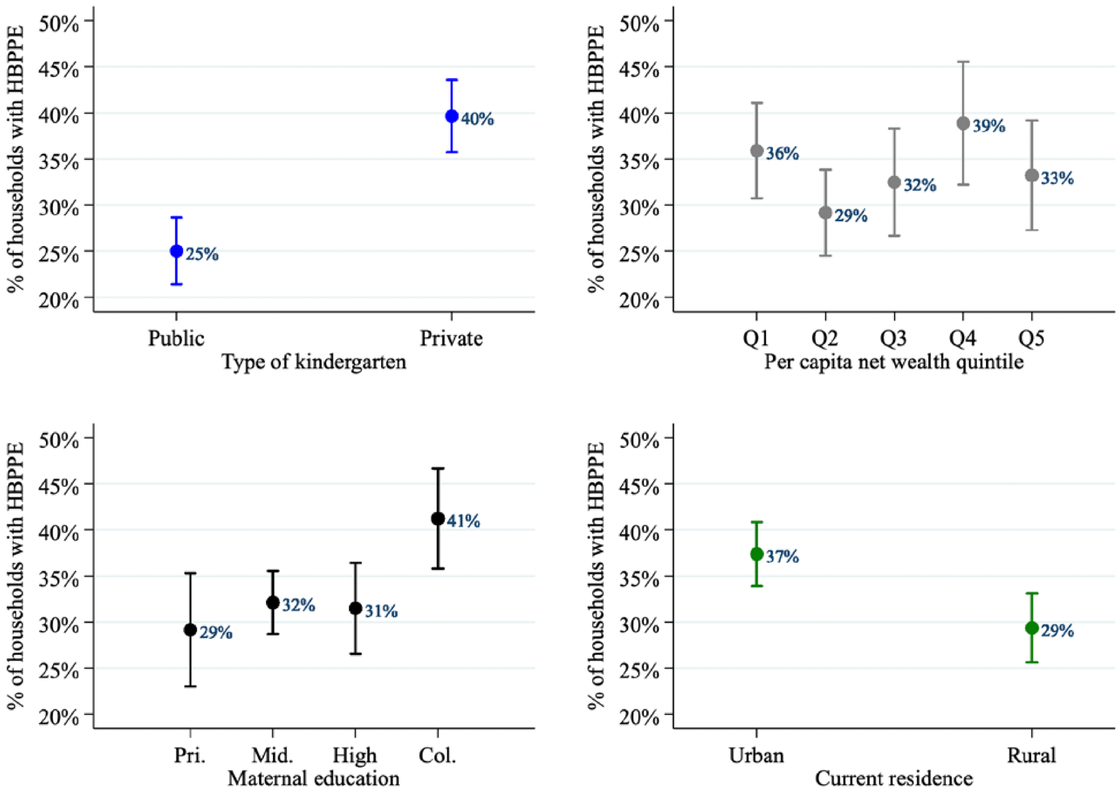

Notes: "Pri.", "Mid.", "High", and "Col." refers to maternal schooling at the primary and below, middle, high, and college and above levels, respectively.

Figure A8. Level and inequalities of % of households with HBPPE (thresehold=20%) by type of kindergarten, maternal education, household net wealth quintile, and current residential area

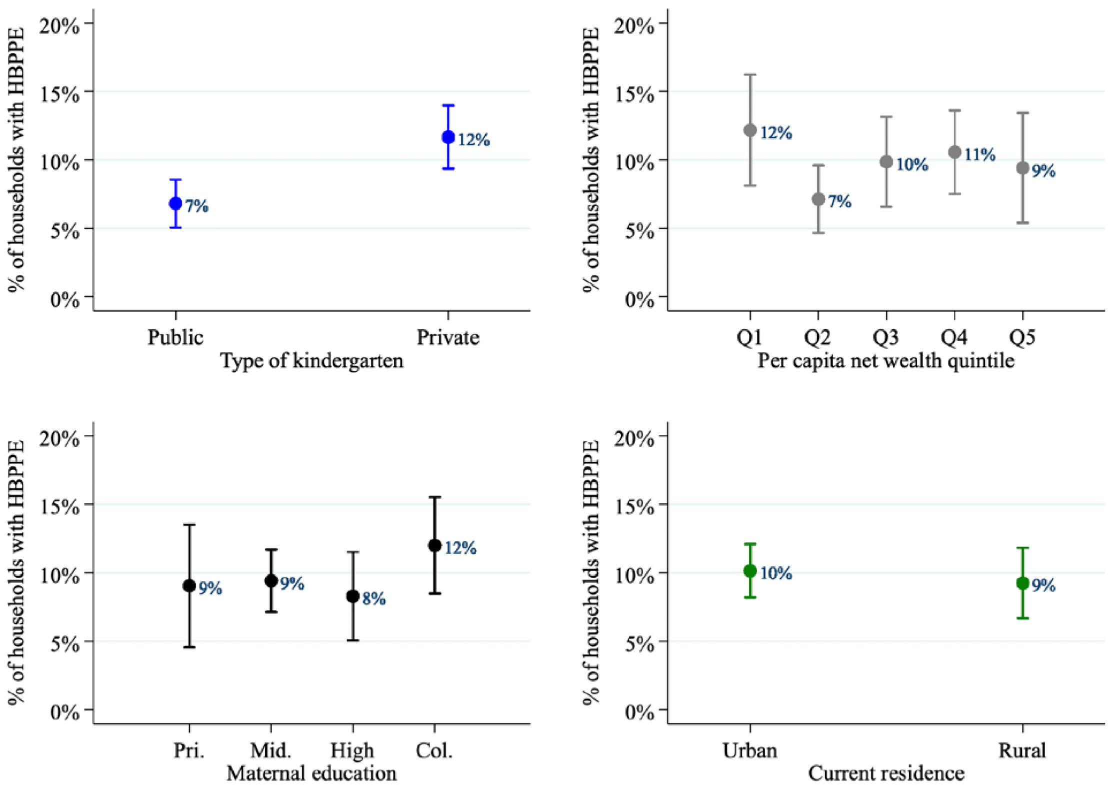

Notes: "Pri.", "Mid.", "High", and "Col." refers to maternal schooling at the primary and below, middle, high, and college and above levels, respectively.

Figure A9. Level and inequalities of % of households with HBPPE (thresehold=7%) by excluding out-school spending from PPE spending

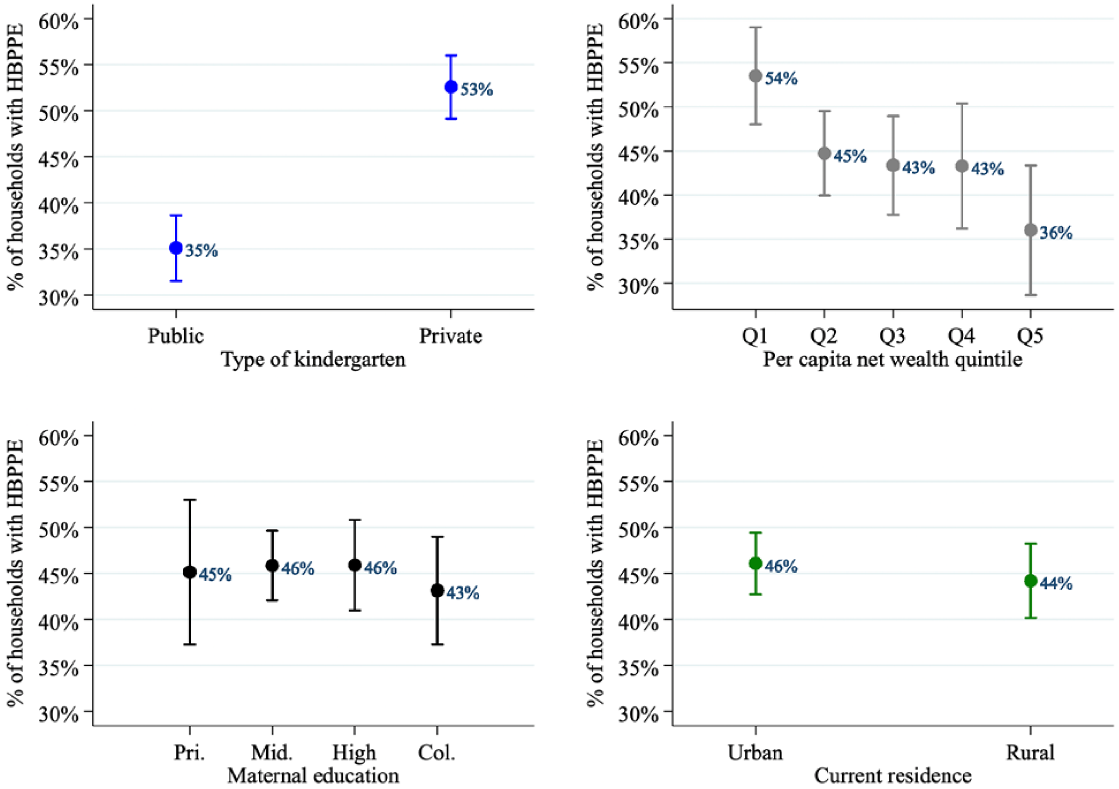

Notes: “Pri.”, “Mid.”, “High”, and “Col.” refers to maternal schooling at the primary and below, middle, high, and college and above levels, respectively.

Wei Y*, et al. BMJ Paediatrics Open* 2023; 7:e001971. doi: 10.1136/bmjpo-2023-001971

Figure A10. Level and inequalities of % of households with HBPPE (thresehold=10%) by excluding out-school spending from PPE spending

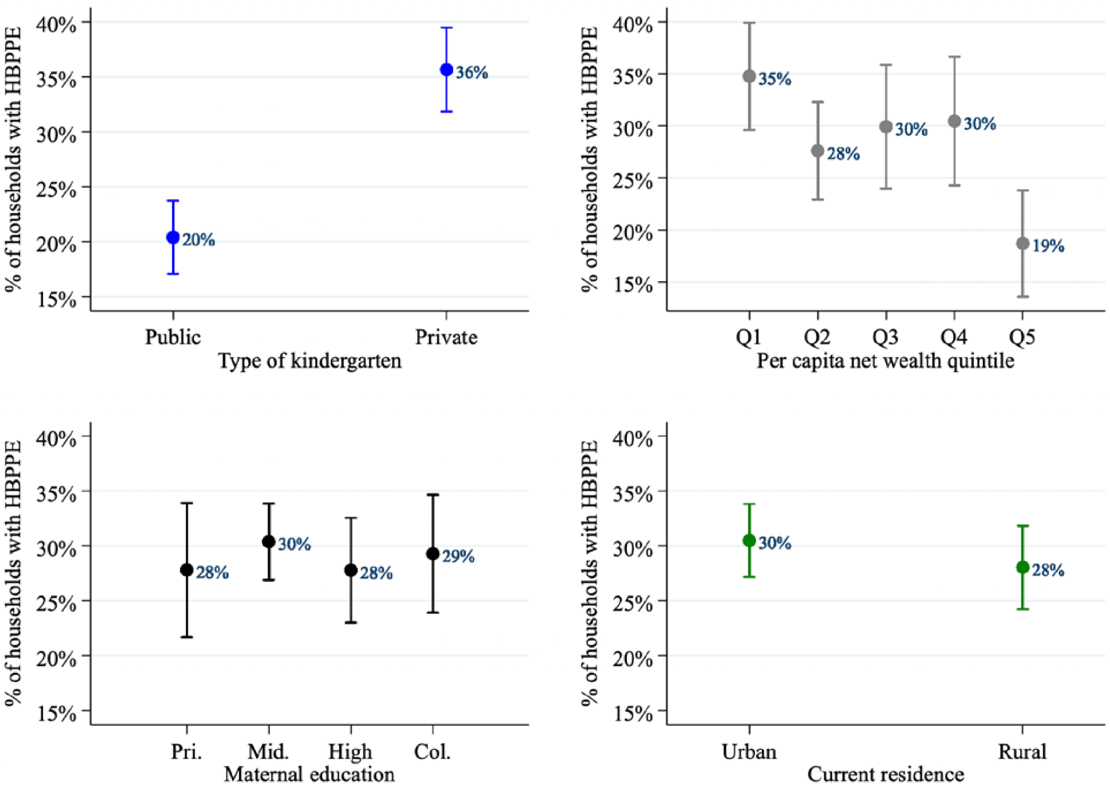

Notes: "Pri.", "Mid.", "High", and "Col." refers to maternal schooling at the primary and below, middle, high, and college and above levels, respectively.

Figure A11. Level and inequalities of % of households with HBPPE (thresehold=20%) by excluding out-school spending from PPE spending

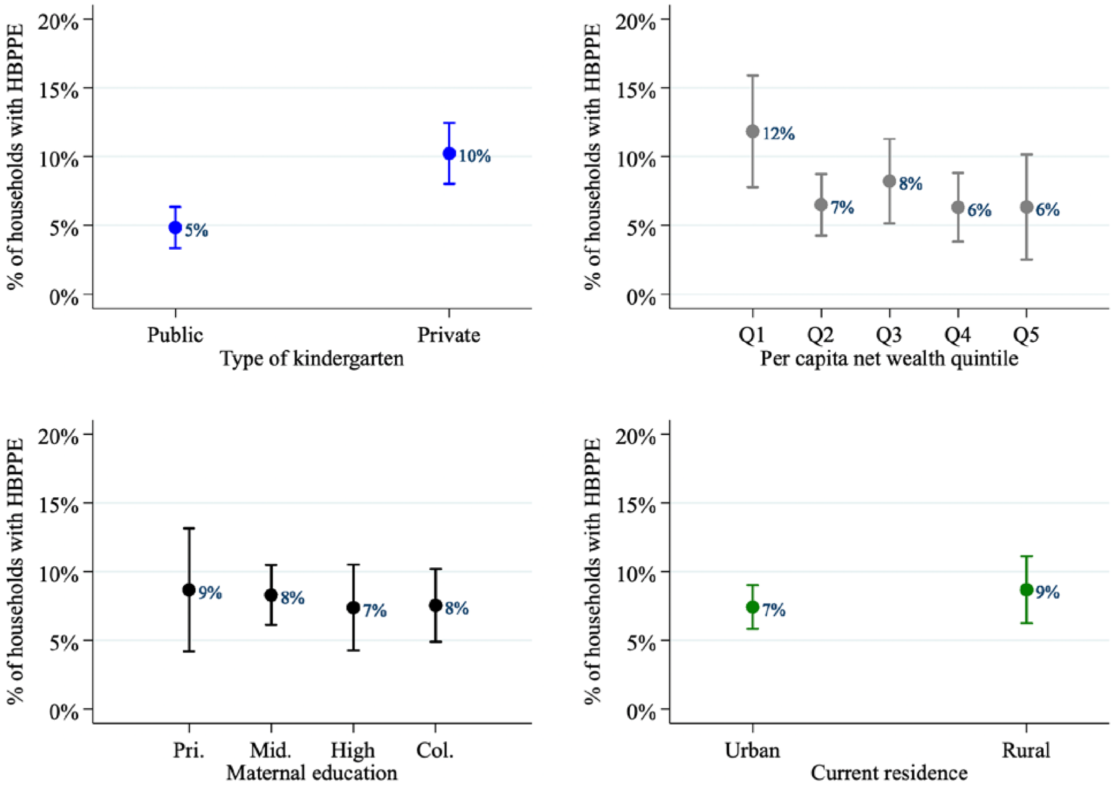

Notes: “Pri.”, “Mid.”, “High”, and “Col.” refers to maternal schooling at the primary and below, middle, high, and college and above levels, respectively.

Figure A12. Level of % of household expenditure on PPE across 29 provinces

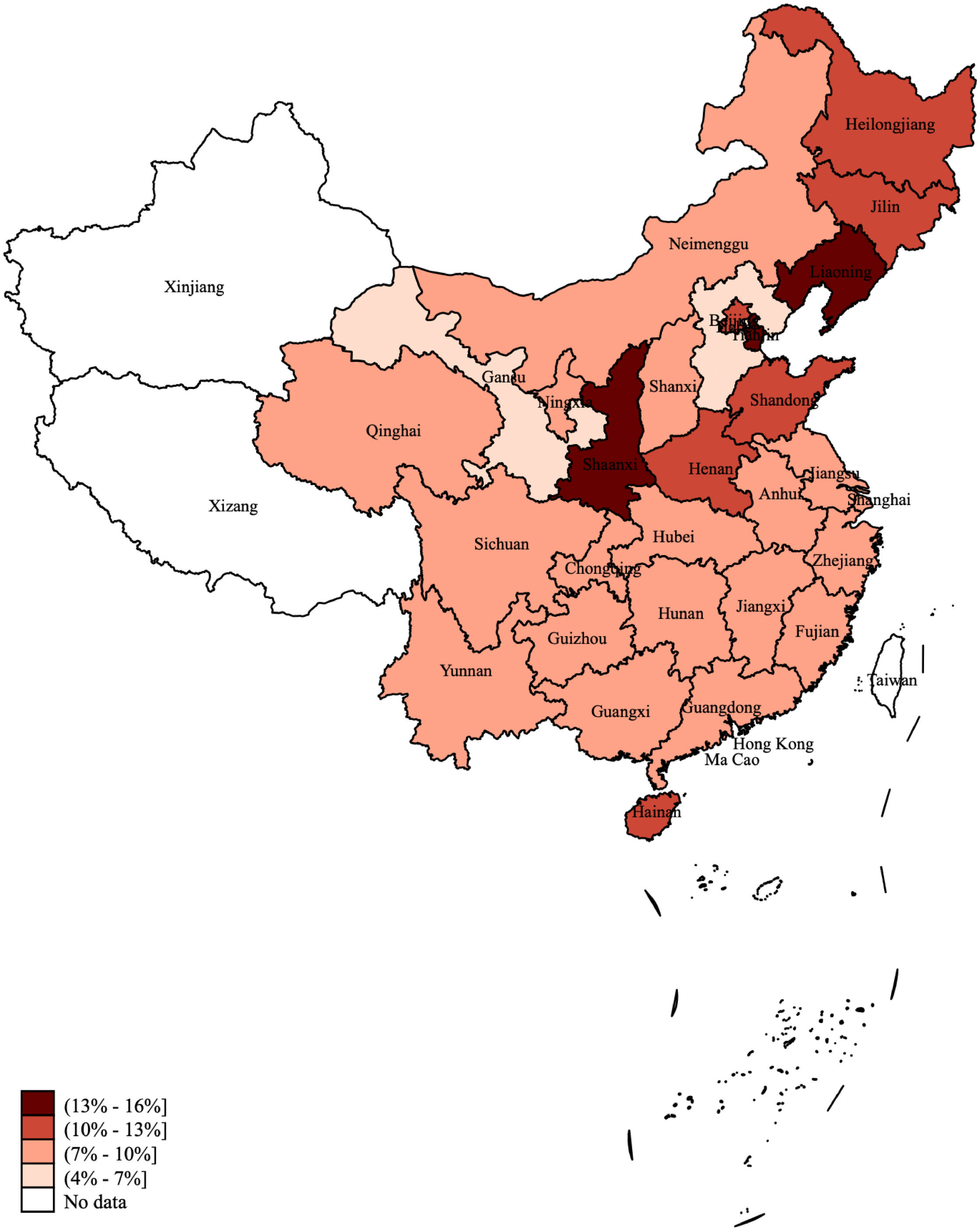

Figure A13. Level and inequalities of % of household expenditure on PPE by type of kindergarten, maternal education, household net wealth quintile, and current residential area

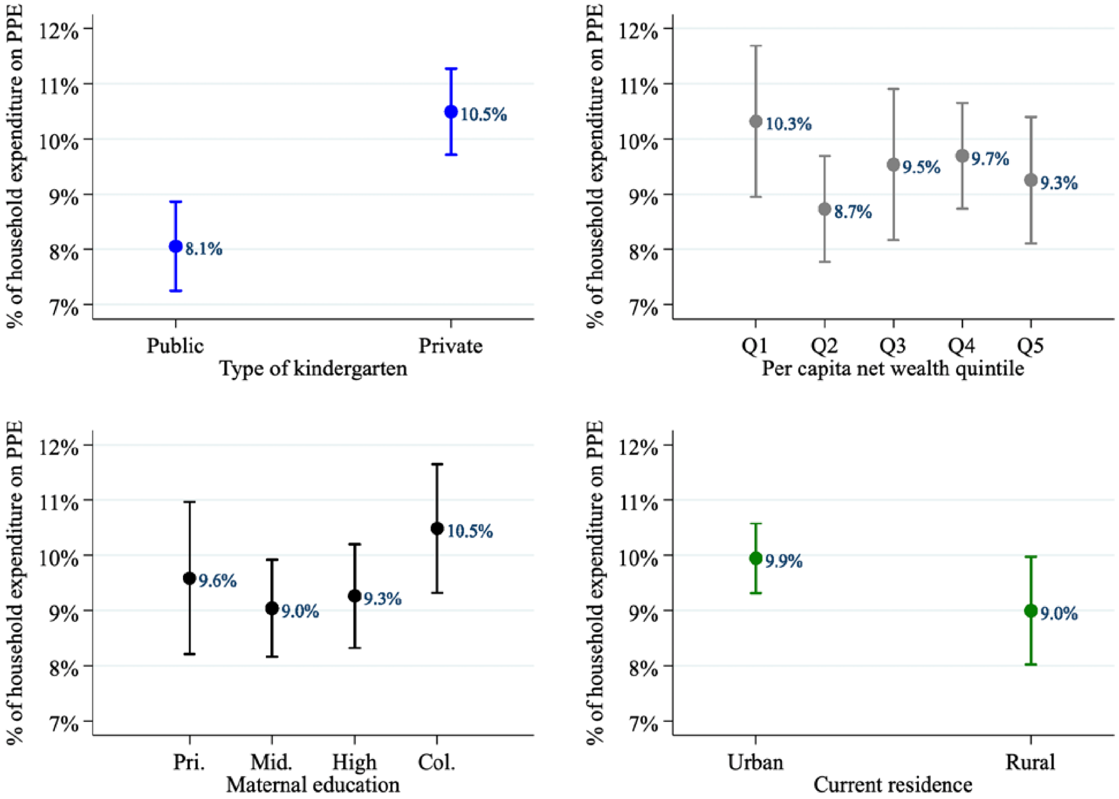

Notes: “Pri.”, “Mid.”, “High”, and “Col.” Refers to maternal schooling at the primary and below, middle, high, and college and above levels, respectively.

Figure A14. Level and inequalities of % of household expenditure on PPE by excluding out-school spending from PPE spending

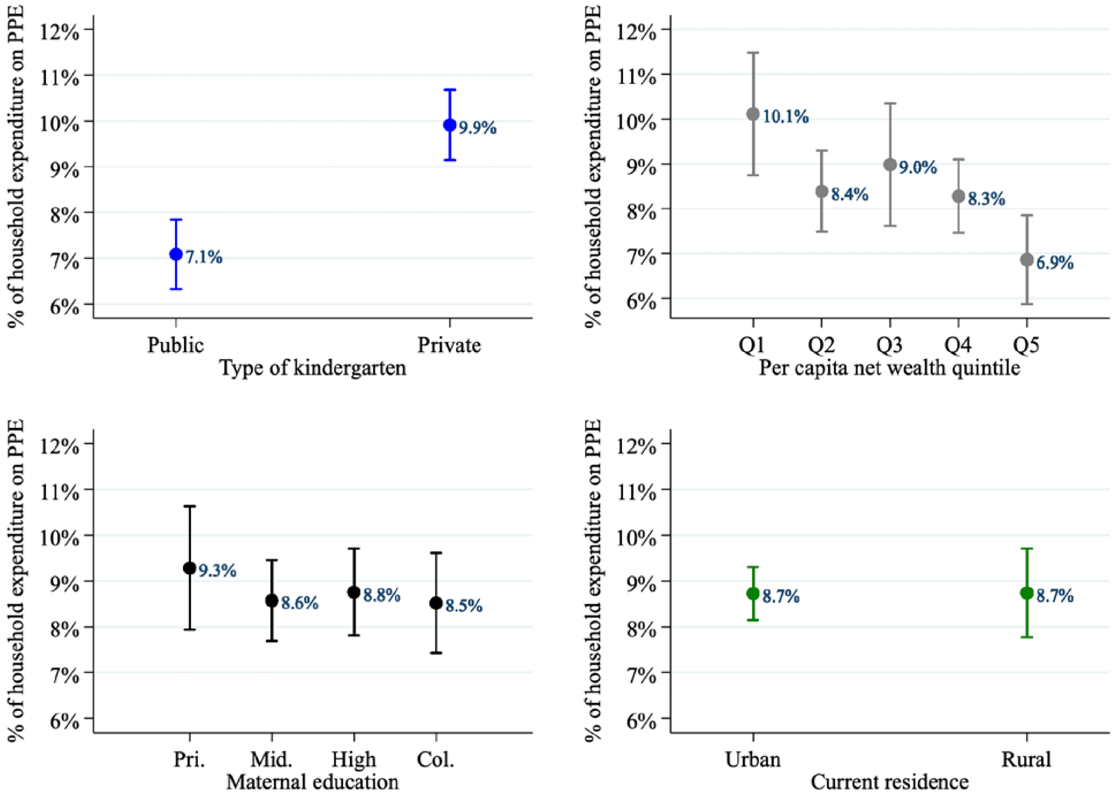

Notes: "Pri.", "Mid.", "High", and "Col." refers to maternal schooling at the primary and below, middle, high, and college and above levels, respectively.

Wei Y, *et al. BMJ Paediatrics Open* 2023; 7:e001971. doi: 10.1136/bmjpo-2023-001971

Table A1. Significance testing of inequalities in PPE spending per child using Cumming and Finch's "rule of eye"

	Mean	Lower bound	Higher bound	Margins of error	Proportion overlap			
Gender					vs female			
Male	7437.056	6631.678	8242.434	805.378	1.763			
Female	7238.254	6369.469	8107.039	868.785				
Private school					vs private			
Public	6193.239	5475.135	6911.343	718.104	**-0.244**			
Private	8075.494	7116.165	9034.824	959.330				
Maternal education					vs middle	vs high	vs college	
Primary	3756.339	3163.960	4348.719	592.379	**-0.615**	**-3.341**	**-6.995**	
Middle	5138.580	4673.767	5603.393	464.813	**.**	**-1.940**	**-6.229**	
High	8068.083	7045.833	9090.333	1022.250	**.**	**.**	**-2.314**	
College	13824.870	12178.490	15471.250	1646.381				
Per capita net wealth quintile					vs Q2	vs Q3	vs Q4	vs Q5
Q1	4182.038	3628.280	4735.797	553.759	0.683	**-1.678**	**-4.956**	**-7.985**
Q2	4813.727	4408.062	5219.393	405.666	.	**-1.117**	**-4.816**	**-8.074**
Q3	6812.270	5935.722	7688.817	876.548	.	**.**	**-1.435**	**-5.051**
Q4	10473.690	9218.501	11728.880	1255.188	.	**.**	**.**	**-1.995**
Q5	17007.120	14991.910	19022.340	2015.218				
Current residence					vs rural			
Urban	10433.780	9434.861	11432.700	998.918	**-7.059**			
Rural	4126.709	3733.122	4520.297	393.588				

Notes: we used Cumming and Finch's "rule of eye" to test the statistical significance of inequalities in PPE spending between the two sub-groups (i.e. whether significant difference existed between the two mean estimates). Details for the analytical methods were presented in **Appendix Panel A**. Numbers in bold indicates a proportion overlap that is less than 0.5; that is a significant statistical relationship at the 0.05 level (p<0.05)

Wei Y, *et al. BMJ Paediatrics Open* 2023; 7:e001971. doi: 10.1136/bmjpo-2023-001971

Table A2. Level of % of households with HBPPE (threshold=7%) across 29 provinces and weighted mean difference of the mean between the provincial level and the national level

Province	Level of % of households with HBPPE at the provincial level	Weighted mean difference
Liaoning	76.9%	0.0086
Shaanxi	67.0%	0.0049
Beijing	64.5%	0.0023
Neimenggu	62.5%	0.0024
Henan	61.2%	0.0082
Hainan	60.0%	0.0007
Tianjin	59.9%	0.0012
Hubei	58.5%	0.0039
Heilongjiang	54.4%	0.0014
Hunan	53.8%	0.0022
Jilin	52.7%	0.0007
Guangxi	52.3%	0.0011
Shandong	51.5%	0.0016
Shanxi	49.3%	0.0000
Fujian	48.7%	-0.0002
Guangdong	47.7%	-0.0013
Jiangsu	46.6%	-0.0016
Sichuan	44.4%	-0.0029
Qinghai	43.9%	-0.0002
Jiangxi	42.2%	-0.0023
Chongqing	41.1%	-0.0018
Guizhou	40.9%	-0.0022
Yunnan	40.5%	-0.0030
Ningxia	40.5%	-0.0004
Anhui	39.9%	-0.0043
Shanghai	39.4%	-0.0017
Zhejiang	36.4%	-0.0054
Hebei	35.8%	-0.0073
Gansu	33.1%	-0.0031

*: The measures took zero as the reference, with positive values indicating provincial means higher than the national average, and negative values indicating provincial means lower than the national average.

Table A3. Level of % of households with HBPPE (threshold=10%) across 29 provinces and weighted mean difference of the mean between the provincial level and the national level

Province	Level of % of households with HBPPE at the provincial level	Weighted mean difference
Liaoning	63.9%	0.0094
Tianjin	49.5%	0.0018
Beijing	49.1%	0.0024
Shaanxi	48.8%	0.0042
Jilin	47.0%	0.0026
Henan	46.1%	0.0086
Hainan	45.2%	0.0008
Shandong	42.5%	0.0064
Heilongjiang	39.2%	0.0015
Chongqing	36.5%	0.0007
Neimenggu	35.1%	0.0003
Hunan	34.2%	0.0003
Guangdong	33.4%	-0.0001
Fujian	32.0%	-0.0004
Hubei	31.9%	-0.0007
Guangxi	31.6%	-0.0007
Guizhou	30.9%	-0.0007
Shanxi	30.3%	-0.0009
Ningxia	29.7%	-0.0002
Jiangsu	29.3%	-0.0024
Yunnan	27.9%	-0.0020
Sichuan	27.6%	-0.0035
Anhui	25.9%	-0.0035
Shanghai	25.5%	-0.0014
Qinghai	23.6%	-0.0004
Zhejiang	23.1%	-0.0044
Jiangxi	22.7%	-0.0036
Hebei	19.7%	-0.0075
Gansu	19.0%	-0.0028

*: The measures took zero as the reference, with positive values indicating provincial means higher than the national average, and negative values indicating provincial means lower than the national average.

Table A4. Level of % of households with HBPPE (threshold=20%) across 29 provinces and weighted mean difference of the mean between the provincial level and the national level

Province	Level of % of households with HBPPE at the provincial level	Weighted mean difference
Liaoning	27.8%	0.0056
Tianjin	23.6%	0.0015
Shaanxi	22.9%	0.0036
Beijing	20.1%	0.0016
Shandong	18.9%	0.0066
Hainan	17.3%	0.0005
Heilongjiang	16.9%	0.0019
Shanxi	12.8%	0.0008
Henan	12.6%	0.0020
Hunan	11.1%	0.0007
Jiangsu	10.2%	0.0003
Yunnan	10.0%	0.0001
Qinghai	9.9%	0.0000
Guangdong	9.3%	-0.0004
Guizhou	8.8%	-0.0002
Jilin	8.4%	-0.0003
Zhejiang	8.3%	-0.0006
Chongqing	6.8%	-0.0006
Neimenggu	6.7%	-0.0005
Hubei	6.4%	-0.0014
Shanghai	5.9%	-0.0007
Guangxi	5.2%	-0.0016
Gansu	5.1%	-0.0009
Ningxia	4.8%	-0.0002
Fujian	4.7%	-0.0014
Sichuan	4.5%	-0.0031
Jiangxi	4.4%	-0.0018
Hebei	4.0%	-0.0031
Anhui	2.9%	-0.0031

*: The measures took zero as the reference, with positive values indicating provincial means higher than the national average, and negative values indicating provincial means lower than the national average.

Table A5. Significance testing of inequalities in HBPPE (threshold=7%) using Cumming and Finch's "rule of eye"

	Mean	Lower bound	Higher bound	Margins of error	Proportion overlap			
Private school					vs private			
Public	0.410	0.372	0.448	0.038	**-1.970**			
Private	0.554	0.520	0.588	0.034				
Maternal education					vs middle	vs high	vs college	
Primary	0.463	0.384	0.542	0.079	1.648	1.645	0.890	
Middle	0.484	0.445	0.523	0.039	.	1.952	0.864	
High	0.486	0.436	0.536	0.050	.	.	1.024	
College	0.537	0.483	0.590	0.054				
Per capita net wealth quintile					vs Q2	vs Q3	vs Q4	vs Q5
Q1	0.544	0.490	0.598	0.054	0.667	0.566	0.980	1.383
Q2	0.476	0.427	0.524	0.049	.	1.803	1.817	1.467
Q3	0.465	0.409	0.521	0.056	.	.	1.651	1.330
Q4	0.486	0.425	0.546	0.061	.	.	.	1.673
Q5	0.507	0.439	0.574	0.068				
Current residence					vs rural			
Urban	0.525	0.493	0.557	0.032	**0.168**			
Rural	0.458	0.417	0.499	0.041				

Notes: we used Cumming and Finch's "rule of eye" to test the statistical significance of inequalities in HBPPE between the two sub-groups (i.e. whether significant difference existed between the two mean estimates). Numbers in bold indicates a proportion overlap that is less than 0.5; that is a significant statistical relationship at the 0.05 level ($p<0.05$)

Wei Y, *et al. BMJ Paediatrics Open* 2023; 7:e001971. doi: 10.1136/bmjpo-2023-001971

Table A6. Significance testing of inequalities in HBPPE (threshold=10%) using Cumming and Finch's "rule of eye"

	Mean	Lower bound	Higher bound	Margins of error	Proportion overlap			
Private school					vs private			
Public	0.250	0.214	0.286	0.036	**-1.873**			
Private	0.396	0.357	0.436	0.039				
Maternal education					vs middle	vs high	vs college	
Primary	0.292	0.230	0.353	0.062	1.384	1.580	**-0.082**	
Middle	0.321	0.287	0.356	0.034	.	1.851	**-0.053**	
High	0.315	0.266	0.364	0.049	.	.	**0.122**	
College	0.412	0.358	0.467	0.054				
Per capita net wealth quintile					vs Q2	vs Q3	vs Q4	vs Q5
Q1	0.359	0.307	0.411	0.052	0.631	1.380	1.498	1.518
Q2	0.292	0.245	0.338	0.047	.	1.367	**0.288**	1.235
Q3	0.325	0.267	0.383	0.058	.	.	0.979	1.875
Q4	0.389	0.322	0.456	0.067	.	.	.	1.104
Q5	0.332	0.273	0.392	0.059				
Current residence					vs rural			
Urban	0.374	0.339	0.409	0.035	**-0.216**			
Rural	0.294	0.256	0.331	0.038				

Notes: Numbers in bold indicates a proportion overlap that is less than 0.5; that is a significant statistical relationship at the 0.05 level (p<0.05)

Table A7. Significance testing of inequalities in HBPPE (threshold=20%) using Cumming and Finch's "rule of eye"

	Mean	Lower bound	Higher bound	Margins of error	Proportion overlap			
Private school					vs private			
Public	0.068	0.051	0.086	0.018	**-0.377**			
Private	0.116	0.093	0.140	0.023				
Maternal education					vs middle	vs high	vs college	
Primary	0.090	0.046	0.135	0.045	1.888	1.806	1.261	
Middle	0.094	0.071	0.117	0.023	.	1.591	1.112	
High	0.083	0.051	0.115	0.032	.	.	0.902	
College	0.120	0.085	0.155	0.035				
Per capita net wealth quintile					vs Q2	vs Q3	vs Q4	vs Q5
Q1	0.122	0.081	0.162	0.041	**0.459**	1.368	1.547	1.318
Q2	0.071	0.047	0.096	0.025	.	1.058	0.760	1.298
Q3	0.099	0.066	0.131	0.033	.	.	1.776	1.881
Q4	0.106	0.075	0.136	0.030	.	.	.	1.676
Q5	0.094	0.054	0.134	0.040				
Current residence					vs rural			
Urban	0.101	0.082	0.121	0.019	1.604			
Rural	0.092	0.067	0.118	0.026				

Notes: Numbers in bold indicates a proportion overlap that is less than 0.5; that is a significant statistical relationship at the 0.05 level ($p<0.05$)

Wei Y, *et al. BMJ Paediatrics Open* 2023; 7:e001971. doi: 10.1136/bmjpo-2023-001971

Table A8. Level of % of household expenditure on PPE across 29 provinces and weighted mean difference of the mean between the provincial level and the national level

Province	Level of % of household expenditure on PPE at the provincial level	Weighted mean difference
Shaanxi	15.4%	0.00163
Liaoning	14.8%	0.00166
Tianjin	13.1%	0.00040
Shandong	12.9%	0.00242
Beijing	12.6%	0.00048
Hainan	11.9%	0.00016
Henan	11.8%	0.00161
Heilongjiang	11.6%	0.00056
Jilin	10.5%	0.00019
Hunan	9.9%	0.00020
Shanxi	9.6%	0.00003
Hubei	9.3%	-0.00010
Guangdong	9.2%	-0.00024
Neimenggu	9.2%	-0.00006
Jiangsu	9.0%	-0.00026
Guizhou	8.8%	-0.00019
Chongqing	8.6%	-0.00020
Guangxi	8.6%	-0.00033
Fujian	8.3%	-0.00034
Qinghai	8.2%	-0.00006
Jiangxi	8.1%	-0.00046
Sichuan	8.0%	-0.00087
Zhejiang	8.0%	-0.00064
Yunnan	7.9%	-0.00055
Shanghai	7.7%	-0.00031
Ningxia	7.3%	-0.00011
Anhui	7.2%	-0.00106
Hebei	6.4%	-0.00169
Gansu	6.0%	-0.00066

Wei Y, *et al. BMJ Paediatrics Open* 2023; 7:e001971. doi: 10.1136/bmjpo-2023-001971

Table A9. Multilevel linear regression results on factors associated with logged % of household expenditure on PPE

	Logged % of household spending on PPE	Logged % of household spending on PPE by excluding out-school spending from PPE spending
Household net wealth quintile (ref. Quintile 1)		
Quintile 2	-0.134***	-0.128**
Quintile 3	-0.204***	-0.210***
Quintile 4	-0.212***	-0.255***
Quintile 5	-0.304***	-0.479***
Mother's education (ref. Primary school and below)		
Middle school	-0.015	-0.019
High school	0.020	-0.007
College/above	0.058	-0.055
Number of children undertaking PPE	0.499***	0.517***
Household having children in private kindergarten	0.331***	0.381***
Household size	-0.099***	-0.093***
Rural	-0.028	-0.011
Constant	-2.775***	-2.901***
N	2949	2935
ICC (Province)	0.033	0.036
ICC (County | Province)	0.150	0.166

Notes: * p<0.10, ** p<0.05, *** p<0.01. We took the natural logarithm of % of household expenditure on PPE to ensure the variable close to a normal distribution. Of note, because 142 out of 3,111 observations reported zero value for this variable, taking log generated 142 missing values. We adopted a multilevel linear model for estimation.

Table A10. Beta regression results on factors associated with % of household expenditure on PPE

	% of household spending on PPE (Odds ratio)	% of household spending on PPE by excluding out-school spending from PPE spending (Odds ratio)
Household net wealth quintile (ref. Quintile 1)		
Quintile 2	0.902**	0.904**
Quintile 3	0.842***	0.840***

Quintile 4	0.862***	0.833***
Quintile 5	0.837***	0.723***
Mother's education (ref. Primary school and below)		
Middle school	1.059	1.054
High school	1.100**	1.082
College/above	1.151***	1.047
Number of children undertaking PPE	1.446***	1.473***
Household having children in private kindergarten	1.319***	1.384***
Household size	0.918***	0.925***
Rural	0.947*	0.961
N	2949	2935

Notes: * p<0.10, ** p<0.05, *** p<0.01. We adopted a beta regression for estimation, which is appropritate for modelling proportional outcome variables that are greater than 0 and less than 1. Of note, because 142 out of 3,111 observations reported zero value for this variable, performing a beta regression would exclude these observations with zero values.

Table A11. Multilevel logit regression results on factors associated with household status on HBPPE by setting household net wealth as a continuous variable

	HBPPE_7% (Odds ratio)	HBPPE_10% (Odds ratio)	HBPPE_20% (Odds ratio)
Log (Household net wealth)	0.870***	0.855***	0.873***
Mother's education (ref. Primary school and below)			
Middle school	1.084	1.012	0.733
High school	0.967	1.035	0.794
College/above	1.164	1.308	1.031
Number of children undertaking PPE	3.363***	3.433***	3.279***
Household having children in private kindergarten	1.962***	2.018***	1.900***
Household size	0.796***	0.757***	0.805***
Rural	0.824*	0.886	1.151
N	2995	2995	2995
ICC (Province)	0.022	0.033	0.062
ICC (County | Province)	0.117	0.112	0.204

Notes: * p<0.10, ** p<0.05, *** p<0.01. Of note, household net wealth was derived by calculating a household' assets minus its debts, leading to 97 household observations that had negative net wealth values. Taking logarithm of household net wealth generated 97 missing values for this variables.

Table A12. Multilevel linear regression and Beta regression results on factors associated with % of household expenditure on PPE by setting household net wealth as a continuous variable

	Logged % of household spending on PPE	% of household spending on PPE

	(Multilevel linear regression)	(Beta regression)
Log (Household net wealth)	-0.065***	-0.041***
Mother's education (ref. Primary school and below)		
Middle school	-0.019	0.041
High school	0.030	0.093*
College/above	0.074	0.146***
Number of children undertaking PPE	0.497***	0.377***
Household having children in private kindergarten	0.336***	0.274***
Household size	-0.099***	-0.085***
Rural	-0.025	-0.048
N	2861	2861

Notes: * p<0.10, ** p<0.05, *** p<0.01.
