# Supplementary material for: Assessing household financial burdens for preprimary education and associated socioeconomic inequalities: a case study in China
Source: BMJ Paediatr Open. 2023 Aug 10;7(1):e001971. doi: 10.1136/bmjpo-2023-001971 (PMC10423832; doi:10.1136/bmjpo-2023-001971)
Supplement: Author's manuscript [file bmjpo-2023-001971.draft_revisions.pdf]

BMJ Paediatrics Open

BMJ Paediatrics Open is committed to open peer review. As part of this commitment we make the peer review history of every article we publish publicly available.

When an article is published we post the peer reviewers' comments and the authors' responses online. We also post the versions of the paper that were used during peer review. These are the versions that the peer review comments apply to.

The versions of the paper that follow are the versions that were submitted during the peer review process. They are not the versions of record or the final published versions. They should not be cited or distributed as the published version of this manuscript.

BMJ Paediatrics Open is an open access journal and the full, final, typeset and author-corrected version of record of the manuscript is available on our site with no access controls, subscription charges or pay-per-view fees (http://bmjpaedsopen.bmj.com).

If you have any questions on BMJ Paediatrics Open's open peer review process please email info.bmjpo@bmj.com

BMJ Paediatrics Open

Assessing household financial burdens for preprimary education and associated socioeconomic inequalities – a case study in China

Journal:	*BMJ Paediatrics Open*
Manuscript ID	bmjpo-2023-001971
Article Type:	Original research
Date Submitted by the Author:	22-Mar-2023
Complete List of Authors:	Wei, Yi; Peking University, China Institute for Educational Finance Research<br>Liu, Kai; Renmin University of China, School of Labor and Human Resources<br>Kang, Le; Nanjing University, Institute of Education<br>Behrman, Jere; University of Pennsylvania, Department of Economics<br>Richter, Linda; University of the Witwatersrand Johannesburg, DSI-NRF Centre of Excellence in Human Development<br>Stein, Alan; Oxford University, Department of Psychiatry<br>Song, Yingquan; Peking University, China Institute for Educational Finance Research<br>Lu, Chunling; Brigham and Women's Hospital, Division of Global Health Equity; Harvard Medical School, Department of Global Health and Social Medicine
Keywords:	Health services research

SCHOLARONE™
Manuscripts

I, the Submitting Author has the right to grant and does grant on behalf of all authors of the Work (as defined in the below author licence), an exclusive licence and/or a non-exclusive licence for contributions from authors who are: i) UK Crown employees; ii) where BMJ has agreed a CC-BY licence shall apply, and/or iii) in accordance with the terms applicable for US Federal Government officers or employees acting as part of their official duties; on a worldwide, perpetual, irrevocable, royalty-free basis to BMJ Publishing Group Ltd ("BMJ") its licensees and where the relevant Journal is co-owned by BMJ to the co-owners of the Journal, to publish the Work in this journal and any other BMJ products and to exploit all rights, as set out in our licence.

The Submitting Author accepts and understands that any supply made under these terms is made by BMJ to the Submitting Author unless you are acting as an employee on behalf of your employer or a postgraduate student of an affiliated institution which is paying any applicable article publishing charge ("APC") for Open Access articles. Where the Submitting Author wishes to make the Work available on an Open Access basis (and intends to pay the relevant APC), the terms of reuse of such Open Access shall be governed by a Creative Commons licence – details of these licences and which Creative Commons licence will apply to this Work are set out in our licence referred to above.

Other than as permitted in any relevant BMJ Author's Self Archiving Policies, I confirm this Work has not been accepted for publication elsewhere, is not being considered for publication elsewhere and does not duplicate material already published. I confirm all authors consent to publication of this Work and authorise the granting of this licence.

Confidential: For Review Only

Assessing household financial burdens for preprimary education and associated socioeconomic inequalities – a case study in China

Yi Wei, Kai Liu, Le Kang, Jere R. Behrman, Linda M. Richter, Alan Stein, Yingquan Song, Chunling Lu

Abstract

Background Providing young children with universal access to preprimary education (PPE) is considered a powerful tool for human-capital development and eliminating the intergenerational transmission of poverty. To remove household financial barrier for achieving universal PPE, this study proposed a measure to identify households incurring "heavy financial burdens from paying for PPE" (HBPPE) and conducted a case study in China.

Methods Using nationally representative data in 2019, we estimated percentage of households with HBPPE (spent 7% or more of their total annual expenditure) and associated socioecnomic inequaities. We also applied a three-level logit-regression model to investigate the factors associated with the probabilities of households incurring HBPPE.

Results Half of the sampled households spent 7% or more of their expenditures on PPE. Households in the lowest wealth quintile (54%) or households with children attending private PPE (55%) had higher percentages of HBPPE than households in other wealth quintiles (e.g., 51% in the highest wealth quintile) or households with children attending public kindergartens (41%). Logit-regression analysis shows that the poorest households and households with children attending private kindergarten were more likely to incur HBPPE than their counterparts.

Conclusion To ensuring universal access to PPE in China, future policy should consider increasing the enrollment of children from low-income families in public kindergartens and increasing governmental investments in low-income households by subsidizing children attending PPE.

Keywords: Access to Kindergarten; Preprimary education, Household financial burden; Socioeconomic inequality

Confidential: For Review O

WHAT IS ALREADY KNOWN ON THIS TOPIC

- Significant socioeconomic inequalities in preprimary education (PPE) attendance were observed in many low- and middle-income countries (LMICs).
- Empirical evidence on household financial barrier to PPE is very limited in LMICs. In China, disadvantaged households (e.g., low-income, low maternal schooling) paid larger share than their counterparts for sending children to preschool. Little, however, is known on how many households suffered from heavy financial burden from paying for PPE.

WHAT THIS STUDY ADDS

- This study proposed a measure to identify households incurring “heavy financial burdens from paying for PPE” (HBPPE) and conducted a case study in China using nationally representative data.
- Findings showed that almost 50% of the sampled households incurred HBPPE (spent over 7% or more of their annual expenditures on PPE), with the poorest households and households in which children attended private kindergarten more likely to incur HBPPE than their counterparts.

HOW THIS STUDY MIGHT AFFECT RESEARCH, PRACTICE OR POLICY

- This study proposed a measure on HBPPE that could be used for similar research in understanding a country’s progress in eliminating financial barrier to universal coverage of PPE.
- Findings of this study could inform policymakers and other stakeholders in China on who suffered HBPPE (e.g., the poorest households or households with children attending private kindergarten) and what could be potentially effective policy instruments in promoting equal access to PPE.

1. Introduction

Preprimary education (PPE) has important influences on the development of cognitive and non-cognitive skills of children, especially for those from disadvantaged groups.[1] The high return on investment in PPE [2-5] makes it an important strategy for strengthening national human capital and reducing inequality and the intergenerational transmission of poverty.[6-8] Quality PPE for all girls and boys has been included in the 2030 Sustainable Development Goals.[9]

Eensuring universal access to quality PPE requires sufficient investment and effective design of cost-sharing mechanisms between governments and households. In many low- and middle-income countries (LMICs), government financial support to PPE remains rather limited,[10-12] making it difficult for the poor households accessing PPE. In Nigeria, for example, less than 10% of the poorest young children attended PPE in 2010, compared to approximately 80% of the richest young Nigerian children.[13]

China has the second-largest child population in the world and has set a policy goal of universal coverage of PPE in 2010.[14] Since then, public expenditures on PPE have increased rapidly from CNY24.44 billion (1.67% of governmental educational spending) in 2010 to CNY 200.8 billion (5.01% of governmental educational spending) in 2019 (**Figure A1 in the appendix**).[15] The gross-enrollment rate for three-year PPE also grew rapidly from 46.1% in 2000 to 83.4% in 2019 (**Figure A2 in the appendix**).[15]

PPE in China is not free. Households have to pay tuition fees that vary greatly by type of kindergarten, public or private. Public kindergartens are essentially “club goods”, which are the extension of the welfare system in the era of planned economy, as a benefit provided by organizations to their “employees”. These public kindergartens are not open to the public and have certain requirements for admission, for example, children of employees of governments, universities, or state-owned enterprises, etc. children from more-advantaged groups are more

For peer review only

likely to enter this kind of public kindergarten with relatively low fees and higher quality.[16,17] Currently, government spending on PPE mainly goes to these public kindergartens, especially the kindergartens run by educational departments and other governmental agencies.

Private kindergartens have become an important part of China's PPE system in recent years, with 56% of young children enrolled in private kindergarten in 2019.[15] The majority of private kindergartens have lower quality and are more expensive than public kindergartens. They are used by children who do not have access to public ones. The major share (85%) of the funding in private kindergartens came from tuition fees contributed by households, compared to 25% in public kindergartens.[18]

Limited empirical evidence has shown that disadvantaged households usually paid larger share of their income on PPE than their counterparts in China.[12,19, 20] However, little is known regarding how many households suffered from heavy financial burdens from paying for PPE (HBPPE). To fill in the gap of knowledge and help policy makers identifying those who need support most, this study proposed a measure to identify households incurring HBPPE and conducted a case study in China. Using the 2019 China Household Finance Survey (CHFS), we assessed the national- and provincial-level prevalence of HBPPE and associated socioeconomic and inequalities. To our knowledge, this is the first study that provides a comprehensive analysis of HBPPE in China using the newly proposed measure and the most recent data.

2. Methods

We used the CHEERSreporting guidelines in writing of this paper.[21]

Ethical statement: No applicable - we used a secondary data that has been published and publicly accessible.[25] Identifiers for individual child have been removed.

2.1. Defining households with heavy financing burdens for paying for PPE (HBPPE)

We adopted the approach used by the World Bank (WB) and the World Health Organization (WHO) in defining catastrophic household health spending: a household has catastrophic health spending if it spends a certain proportion or more of its total annual expenditures (10% for the WB[22]) or annual non-food expenditures (10-40% for the WHO[23]) on health. This study defined a household incurring HBPPE if it spends more than 7% of its total annual expenditures on PPE. The selection of "7%" was based on a federal benchmark for households receiving subsidies on PPE proposed by the Office of Child Care under the U.S. Department of Health and Human Services, stating that households receive subsidies if their out-of-pocket spending on PPE exceed 7% of households' incomes.[24] The policy suggests that PPE spending more than 7% of household income could be considered burdensome to households. To conduct sensitivity analysis, we allowed variation in this threshold among 7%, 10%, and 20%.

2.2. Data and samples

We used nationally and provincially representative data from the 2019 China Household Finance Survey (CHFS) that collects information on household assets, income, wealth, consumption, as

Confidential: For Review Only

well as socioeconomic and demographic characteristics of the sampled households and their members.[25] The CHFS employs a stratified three-stage random sample design (details in **Chapter 1 and Figure A3 of the Appendix**). The 2019 CHFS data is publicly accessible and covers 29 provinces (excluding Tibet, Xinjiang, Inner Mongolia, Hong Kong, and Macau), 345 cities/districts/counties, 34,643 households, and 107,008 individuals. Our study focused on households with children, aged 3-6 years, attending kindergarten. The final sample includes 3,371 young children attending kindergartens, from 3,111 households (9% of the total sample), who reported available PPE expenditure data.

2.3. Measuring households with HBPPE

To assess if household suffered from HBPPE, we calculated estimates of household spending on PPE and total expenditures in the previous 12 months. Caregivers reported household expenditure on kindergarten over the 12 months prior to the survey. The expenditure can be categorized into two groups:(i) fees charged by schools (in-school expenditures) including tuition fees, books and materials, uniforms, meals, transport, and accommodation; (ii) fees on out-of-school education (out-of-school expenditure) including after-school tutoring (academically focused), extracurricular activities (e.g., learning piano or sports), and home-learning materials (e.g., books, computers). To estimate the sensitivity of the findings to the definition of PPE spending, we excluded out-of-school spending from PPE spending in an alternative analysis.

The CHFS includes a series of questions on household consumption over the 12 months prior to the survey that cover spending on food, clothing, housing, daily necessities and service, health care, transportation and communication, education and entertainment, and others (e.g. legal service). We obtained the annual household expenditure by summing spending on these items. For each household, we calculated the percentage of household total expenditure devoted to PPE. Based on the cutoff percentage value (7%, 10%, or 20%), we then constructed a dichotomous variable indicating a household's status of HBPPE: if a household spent more than 7% (10% or 20%) of its total expenditure on PPE, we classified the household as experiencing HBPPE.

2.4. Measuring socioeconomic disparities in HBPPE

We assessed the disparities in the percentage of households with HBPPE by household economic status (five wealth quintiles), maternal schooling (primary school or below, middle school, high school, and college or above), residential area (rural vs. urban), and type of kindergarten (public vs. private). Disparity in this study was quantified as the absolute differences between the two sub-groups. Details on constructing these variables are presented in **Chapter 2 of the Appendix**.

2.5. Statistical Analysis

We first performed a descriptive analysis by assessing the level and socioeconomic inequalities in PPE spending per child and percentages of households with HBPPE. All estimates were adjusted for sampling weights (individual and household weights), primary sampling unit (county), and stratification variable (province). We estimated the statistical significance of inequalities in PPE spending and % of households with HBPPE between the two sub-groups (e.g.

Confidential: For Review Only

rural vs urban)[26] (details in **Chapter 3 in the Appendix**).

Considering the large variation in economic development and geographical situations across the provinces, we assessed the provincial-level disparities in the level of PPE spending per child and % of households with HBPPE across the 29 provinces, using the gap between the weighted mean of each province from the national mean (details in **Chapter 4 of the Appendix**).

To identify the factors associated with the probabilities of a household incurring HBPPE, we used a three-level logit regression model with random intercepts by province (level 3) and county (level 2), controlling for potential clustering effects at the province and county levels (Equation 1).

$$Logit\ (Y_{ijk}) = \beta_0 + \boldsymbol{\beta X_{ijk}} + u_{0k} + v_{0jk} + \varepsilon_{0ijk} \quad (1)$$

where Logit(Y_{ijk}) represents the probability of incurring HBPPE in the *i*th household who lives in county *j* and province *k*. **β** is a vector of coefficients for $\mathbf{X}_{ijk}$ which is a vector of variables with individual or household characteristics, including the number of children undertaking PPE, children attending private kindergarten, household size, maternal schooling, wealth quintile, and residential area. u_{0k} and v_{0jk} represent between-province random variations and between-county/within-province random variations, respectively. ε_{0ijk}represent random errors. Summary statistics for these variables are presented in **Table 1**.

Regression analyses were conducted using three different thresholds for HBPPE: 7%, 10%, and 20%. The STATA program (Version 16.1) was used in analyses.

3. Results

Among all the children attending kindergarten, 47.0% were girls, and 56.0% attended private kindergarten. Among the total of 3,111 households, 35.2% lived in rural areas; 11.9% or 21.0% were in the highest or lowest wealth quintile, respectively; 24.8% of the mothers held college degrees or higher, whereas over 57.2% less than a high-school diploma (**Table 1**).

3.1. Level and inequalities in household PPE spending per child

On average, PPE spending per child at the national level was CNY 8,220 (**Table 1)**, ranging from CNY4,070 in Qinghai province to CNY 23,348 in Beijing (**Appendix Figure A4 and Table A1**). Well-developed provinces had much higher PPE spending per child than the under-developed central and western provinces (**Appendix Table A1**).

For households sending children to private kindergartens, or in urban areas, or with college-educated mothers, or from the highest wealth quintile, their PPE spending per child was significantly higher than that of their counterparts (**Figure 3**). For example, PPE spending per child at the private kindergartens was approximately 30% higher higher than that at public kindergartens (CNY8,075 vs. CNY6,193). PPE spending per child in the richest households was

Confidential: For Review Only

four times more than that in the poorest households (CNY17,007 vs. CNY4,182) (statistical testing in **Appendix Table A2**).

When excluding out-of-school expenditure from PPE spending, the main results remain robust (**Appendix Figure A5)**.

3.2. Level and inequalities in the percentage of HBPPE

Approximately 49.5% of households at the national level spent more than 7% of their total annual expenditure on paying for PPE, our initial threshold for HBPPE. This percentage reduced to 34.2% or 9.8% respectively if the threshold was set at 10% or 20% (**Table 1**). Provinces in the Northeastern and Middle areas of China had higher percentages of HBPPE than other areas, with Liaoning province (76.9%) the highest (**Figure 2, Table A3**). When using alternative thresholds (10% or 20%), the percentage of households with HBPPE in each province was reduced, but the pattern across the provinces remained stable (**Appendix Figures A6 and A7 and Tables A4 and A5)**.

In terms of socioeconomic inequalities (**Figure 3**), % of HBPPE was higher among those households sending children to private kindergartens (55%) than those to public kindergartens (41%). The disparity remained significant when using alternative thresholds (**Appendix Figures A8 and A9**, **Appendix Tables A7 and A8)** or when excluding out-of-school spending from PPE spending (**Appendix Figures A10-12**). % of HBPPE among the richest households was significantly lower than that among the poorest households when using threshold of 7% or 10%, after excluding out-of-school spending from PPE spending.

3.3. Factors associated with household HBPPE

Regression results (**Table 2**) have identified the following factors associated with HBPPE, across the three thresholds: households in the higher wealth quintiles or with smaller size were less likely to incur HBPPE than their counterparts; households sending children to private kindergarten or with more children in kindergarten were more likely to incur HBPPE than their counterparts.

4. Discussion

This study proposed a measure to identify households incurring "heavy financial burdens from paying for PPE" (HBPPE) and applied the measure to the data in China. Our findings showed that, in China, approximately 50% (34%, 10%) of the households with young children attending kindergarten suffered from HBPPE, when a threshold of 7% (10%, 20%) of their annual expenditures on PPE was applied. Households from provinces in the Northeastern and Middle Regions had higher percentages of HBPPE on average than those from other areas. Holding everything else constant, households in the lowest-wealth quintile or sending children to private kindergarten incurred the higher probability of HBPPE than their counterparts.

Confidential: For Review Only

These findings are consistent with previous studies that indicate variation of PPE spending by household income levels and type of kindergartens.[12,19,20] Our study has further identified significant disparities in household with HBPPE, and found that households with children attending private kindergartens had an increase of 88%-105% (across the three thresholds) in the odds of incurring HBPPE compared to households with children attending public kindergartens. The richest households could have a reduction of 37%-50% in the odds of incurring HBPPE compared to the poorest households. The finding of regional disparities in HBPPE is consistent with empirical evidence regarding the slowdown of economic development in the Northeastern and Middle Regions[27] and their lower percentage of public spending on compulsory education than other regions[28,29] and on PPE[30] in particular.

To ensure universal PPE, the Chinese government has initiated the policy of increasing the supply of public kindergartens and regulating the tuition fees of private kindergartens. As shown in our study, even with the expansion of public kindergartens in recent years, more than half of young children still had to go to expensive private kingdergarten. To reduce the financial burden of PPE for households, the Chinese government needs to continue increasing the public kindergartens and make them accessible to everyone. Meanwhile, providing PPE subsidies to low-income households, especially those with children going to the private kindergartens, is necessary for ensuring the poor children having access to affordable PPE.

This study has several limitations. First, the study only included children enrolled in kindergarten at the time of the survey. Children who were not able to attend kindergarten were not included in the analysis sample and they were more likely to live in poor or remote rural areas with limited or even no access to kindergarten. Excluding those children could lead to an underestimation of the probability of HBPPE among the poorest or rural households. Second, the study only used cross-sectional data. With a new wave of survey data available in the future, we will be able to identify the impact of related governmental policies on HBPPE over time. Third, the study used three thresholds to measure households with HBPEE. However, there is no information from households on what percentage of their total spending on PPE would be considered affordable.

Despite the limitations, this study proposed a measure that can be used by researchers to identify households with financial barrier for accessing PPE in LMICs. Using this measure in China, the study provides solid evidence to inform policy makers on who suffered HBPPE and what could be potentially effective policy instruments in promoting equal access to PPE. Future PPE policy in China should consider establishing a financial-aid system on PPE for the children who need it most and expanding the enrollment of children from disadvantaged households in the public kindergarten system.

Confidential: For Review Only

Source of funding
This work was supported by Ministry of Educaiton in China (7122900028), UNICEF China Office (8430500872), and the UKRI GCRF Harnessing the Power of Global Data to Advance Young Children's Learning and Development (ES/T003936/1).

Conflict of interest
The authors have declared no competing interests.

Authors' contribution
YW was responsible for survey coordination , conducted data analysis, and wrote the first draft of the manuscript; LK drew the map that demonstrates the sample distribution in the survey as well as the financial burdens in PPE at provincial level. KL conducted data analysis on inequalities and regressions and took responsibility for the integrity of data analysis. YS was the PI for expenditures of PPE, survey design, initiated the study, supervised YW's work. CL conceptualized the study, designed the analysis plan, and mentored data analysis. All authors have made contributions to data interpretation and manuscript writing. All authors approve the manuscript for submission.

Data sharign statement
Data from the China Household Finance Survey (CHFS) is available at the website of the Survey and Research Center for China Household Finance, at https://chfs.swufe.edu.cn/
The analysis code is available from the corresponding author upon request.

References

1. Black, M. M., Walker, S. P., Fernald, L., Andersen, C. T., DiGirolamo, A. M., Lu, C., McCoy, D. C., Fink, G., Shawar, Y. R., Shiffman, J., Devercelli, A. E., Wodon, Q. T., Vargas-Barón, E., Grantham-McGregor, S., & Lancet Early Childhood Development Series Steering Committee. (2017). Early childhood development coming of age: science through the life course. *Lancet*, 389(10064), 77–90.
2. Chetty, R., Friedman, J. N., Hilger, N., Saez, E., Schanzenbach, D. W., & Yagan, D. (2011). How does your kindergarten classroom affect your earnings? Evidence from Project Star. *Quarterly Journal of Economics*, 126(4), 1593–1660.
3. Heckman, J., Pinto, R. & Savelyev, P. (2013). Understanding the mechanisms through which an influential early childhood program boosted adult outcomes. *American Economic Review*, 103 (6), 2052-86.
4. Holla, Alaka, Magdalena Bendini, Lelys DinarteIva, Iva Trako. (2021). Is Investment in Preprimary Education Too Low? Lessons from (Quasi) Experimental Evidence across Countries. Washington, DC: World Bank Policy Research Working Paper; No. 9723.
5. Nores, M., & Barnett, W. S. (2010). Benefits of early childhood interventions across the world: (Under) Investing in the very young. *Economics of Education Review, 29(2), 271–282.*
6. Berlinski, S., & Schady, N. (2015). The Early Years: Child Well-being and the Role of Public Policy. MacMillan Press, New York.

Confidential: For Review Only

7. Engle, P. L., Fernald, L. C., Alderman, H., Behrman, J., O'Gara, C., Yousafzai, A., Cabral de Mello, M., Hidrobo, M., Ulkuer, N., Ertem, I., Iltus, S., & the Global Child Development Steering Group. (2011). Strategies for reducing inequalities and improving developmental outcomes for young children in low-income and middle-income countries. *Lancet,* 378(9799), 1339–1353.
8. OECD. (2006). Starting Strong II: Early Childhood Education and Care. OECD Publishing, Paris.
9. Sustainable Development Goals, 2030. Retrieved from: https://www.undp.org/sustainable-development-goals

10. UNESCO. Institute for Statistics. http://uis.unesco.org/en/files/who-pays-what-education-national-revealed-through-accounts-2016-en-pdf

11. Gil, J.D., Ewerling, F., Ferreira, L. Z., & Barros, A.J. (2020). Early childhood suspected developmental delay in 63 low- and middle-income countries: Large within- and between-country inequalities documented using national health surveys. *Journal of global health*,10(1), 010427.

12. Su, Y. & Rao, N. (2020). Relationship between family's socioeconomic status and parental investments in early learning: Evidence from the China Family Panel Studies. *Peking University Education Review*, 18(3), 86-103. In Chinese
13. Lu, C., Cuartas J, Fink G, et al. (2020). Inequalities in early childhood care and development in low/middle-income countries: 2010–2018. *BMJ Global Health* 2020;5:e002314.
14. Ministry of Education of the People's Republic of China. http://www.moe.gov.cn/publicfiles/business/htmlfiles/moe/moe_2792/index.html

15. China Educational Finance Statistical Yearbook (2020).
16. Song, Y. (2019). Principal contradictions and reformation of public finance in early childhood education and care in China. *China Economics of Education Review*, 4(3), 19-48. In Chinese
17. Yuan, L., & Zhao, J. (2019). Issues in the fiscal policy for preschool education. *China Economics of Education Review*, 2019,4(03), 49-63. In Chinese
18. Ministry of Education of the People's Republic of China. (2020). Educational statistics in 2019. Retrieved from: http://www.moe.gov.cn/jyb_sjzl/moe_560/jytjsj_2019/qg/
19. Tian, Z., Zhang, X., & Yuan, L. (2011). The disparities of preschool educational resources for children with different household registration: A survey in Beijing. *Renmin University of China Education Journal*, (03), 165-180. In Chinese
20. Liu, Y., & Song, Y. (2013). Survey on the family expenditure for pre-school education of 3-to-6-year-old children in urban families. *Journal of Central China Normal University (Humanities and Social Sciences)*, 52(01), 155-160. In Chinese
21. Husereau D, Drummond M, Petrou S, Carswell C, Moher D, Greenberg D, Augustovski F, Briggs AH, Mauskopf J, Loder E. Consolidated Health Economic Evaluation Reporting Standards (CHEERS) statement.
22. Wagstaff, A., Glores G, Hsu J. et al. Progress on catastrophic health spending in 133 countries: a retrospective observational study. *Lancet Global Health* 2018;6:e169-79.

Confidential: For Review Only

23. Xu, K., Evans, D. B., Kawabata, K., Zeramdini, R., Klavus, J., & Murray, C. J. (2003). Household catastrophic health expenditure: a multicountry analysis. *Lancet*, 362(9378), 111–117.
24. Office of Child Care (OCC), Administration for Children and Families (ACF), Department of Health and Human Services (HHS). (2016). Child Care and Development Fund (CCDF): Final rule. Retrieved from https://www.federalregister.gov/documents/2016/09/30/2016-22986/child-careand-development-fund-ccdf-program
25. Survey and Research Center for China Household Finance. https://chfs.swufe.edu.cn/
26. Cumming, G., & Finch, S. (2005). Inference by eye: Confidence intervals and how to read pictures of data. *American Psychologist*, 60(2),170-180.
27. Wang, J., Yuan, C.,& Ma, N. (2022). Government competition, factor flow and regional collapse. *Journal of Xi'an Jiaotong University (Social Science)*, 42(2), 40-52. In Chinese
28. Shang, W., Lu, S., & Li, T. (2020). The collapse of central regions in the development of compulsory education in China: Representation, influencing factors, and policy rationales. *Peking University Education Review*, 18(2), 172-192. In Chinese
29. Lei, W., Qian, J.,& Ma, H. (2014). Studies on compulsory education investment collapse in central China. *Economics and Education*, 2014(6), 1-9. In Chinese
30. Li, Z., Gong, X., & Qian, J. (2018). Studies on preschool investment sinking in central China. *China Economics of Education Review*, 3(4), 78-90. In Chinese

Confidential: For Review Only

Table 1. Summary statistics of variables used in the analysis

Variable	Observation	Mean	SD	Min	Max
Child-level variables					

PPE expenditure per child	3,371	8,220	10,088	0.000	204,000
Female	3,371	0.470	0.499	0.000	1.000
Private kindergarten	3,371	0.560	0.496	0.000	1.000
Household-level variables					
PPE expenditure per household (CNY)	3,111	8,907	10,824	0.000	204,000
% of household expenditure on PPE	3,111	0.094	0.096	0.000	0.887
HBPPE (7%)	3,111	0.495	0.500	0.000	1.000
HBPPE (10%)	3,111	0.342	0.475	0.000	1.000
HBPPE (20%)	3,111	0.098	0.297	0.000	1.000
Number of children undertaking PPE	3,111	1.084	0.294	1.000	4.000
Children in private kindergarten	3,091	0.557	0.497	0.000	1.000
Household size	3,111	5.131	1.576	2.000	15.000
Per capita household net wealth (CNY)	3,111	241,004	562,588	-1,974,739	17,020,206
Household net wealth quintile	3,111				
Quintile 1	3,111	0.210	0.407	0.000	1.000
Quintile 2	3,111	0.252	0.434	0.000	1.000
Quintile 3	3,111	0.232	0.422	0.000	1.000
Quintile 4	3,111	0.187	0.390	0.000	1.000
Quintile 5	3,111	0.119	0.324	0.000	1.000
Maternal schooling					
Primary school and below	3,111	0.136	0.343	0.000	1.000
Middle school	3,111	0.436	0.496	0.000	1.000
High school	3,111	0.180	0.384	0.000	1.000
College or above	3,111	0.248	0.432	0.000	1.000
Rural residential area	3,111	0.352	0.478	0.000	1.000

Notes: The number of children undertaking PPE indicates the number of children who attended kindergarten in a household. Household size is defined as the total number of household members in a household. A household unit is defined as all members of a housing unit related by blood, marriage, or some other legal arrangement; or two or more persons use incomes to make joint expenditures; or a single person living with others but is financially independent. Thus a household may include grandparents, especially in rural area. When analyzing households that have children in private kindergarten, we excluded the observations of 20 households that had children attending both public and private kindergarten. CNY: Chinese Yuan.

Confidential: For Review Only

Table 2. Multilevel logit regression results on factors associated with household status on HBPPE defined with various thresholds

	HBPPE_7% (Odds ratio)	HBPPE_10% (Odds ratio)	HBPPE_20% (Odds ratio)
Household net wealth quintile (ref. Quintile 1)			

Quintile 2	0.833*	0.756**	0.665**
Quintile 3	0.560***	0.584***	0.581***
Quintile 4	0.598***	0.697***	0.595**
Quintile 5	0.634***	0.512***	0.503***
Mother's education (ref. Primary school and below)			
Middle school	1.192	1.200	1.095
High school	1.004	1.127	1.146
College/above	1.208	1.391*	1.428
Number of children undertaking PPE	3.451***	3.375***	3.233***
Household having children in private kindergarten	1.973***	2.050***	1.880***
Household size	0.799***	0.766***	0.789***
Rural	0.820**	0.892	1.225
Constant	0.744	0.407***	0.061***
N	3091	3091	3091
ICC (Province)	0.018	0.029	0.048
ICC (County | Province)	0.110	0.108	0.187

Notes: * p<0.10, ** p<0.05, *** p<0.01. ICC refers to intraclass correlations; ICC (Province) denotes the intraclass correlations at the province level, and ICC (County | Province) denotes the intraclass correlations at the county and province level. When we define the HBPPE measure with the threshold of 7%, for example, we estimated that province random effects composed 1.8% of the total residual variance of the HBPPE measure, while county and province random effects composed 11.0% of the total residual variance of the HBPPE measure.

Confidential: For Review Only

Figure 1. Level and inequalities of PPE spending per child by gender, type of kindergarten, maternal education, household net wealth quintile, and current residential area

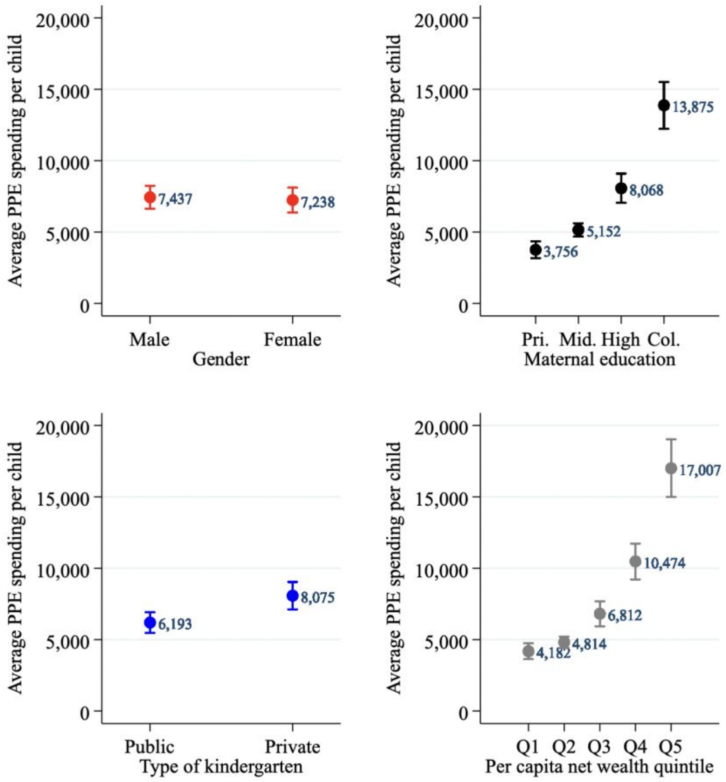

Notes: “Pri.”, “Mid.”, “High”, and “Col.” refers to maternal schooling at the primary and below, middle, high, and college and above levels, respectively.

For Peer Review Only

Figure 2. Level of % of households with HBPPE (threshold=7%) across 29 provinces

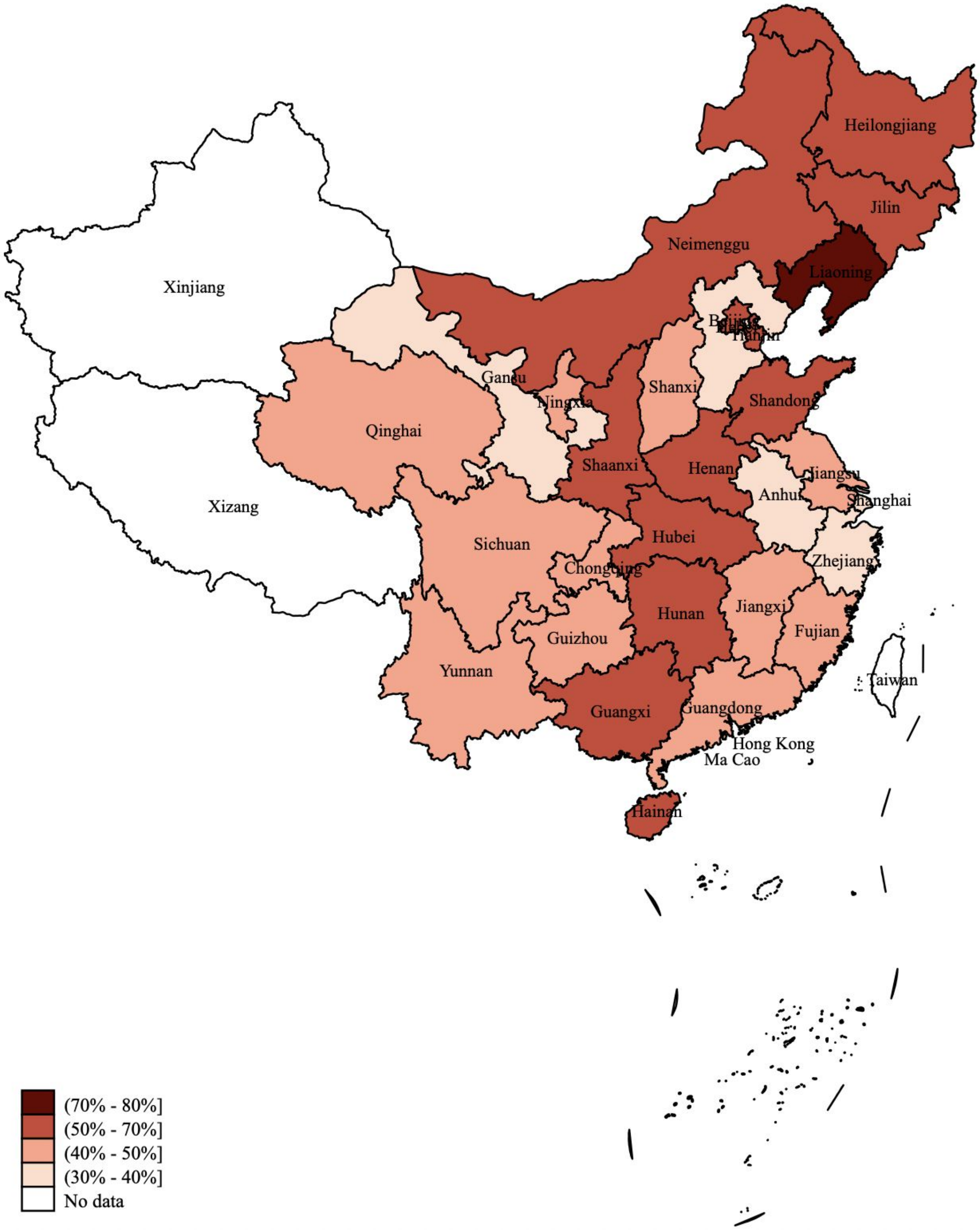

Figure 3. Level and inequalities of % of households with HBPPE (thresehold=7%) by type of kindergarten, maternal education, household net wealth quintile, and current residential area

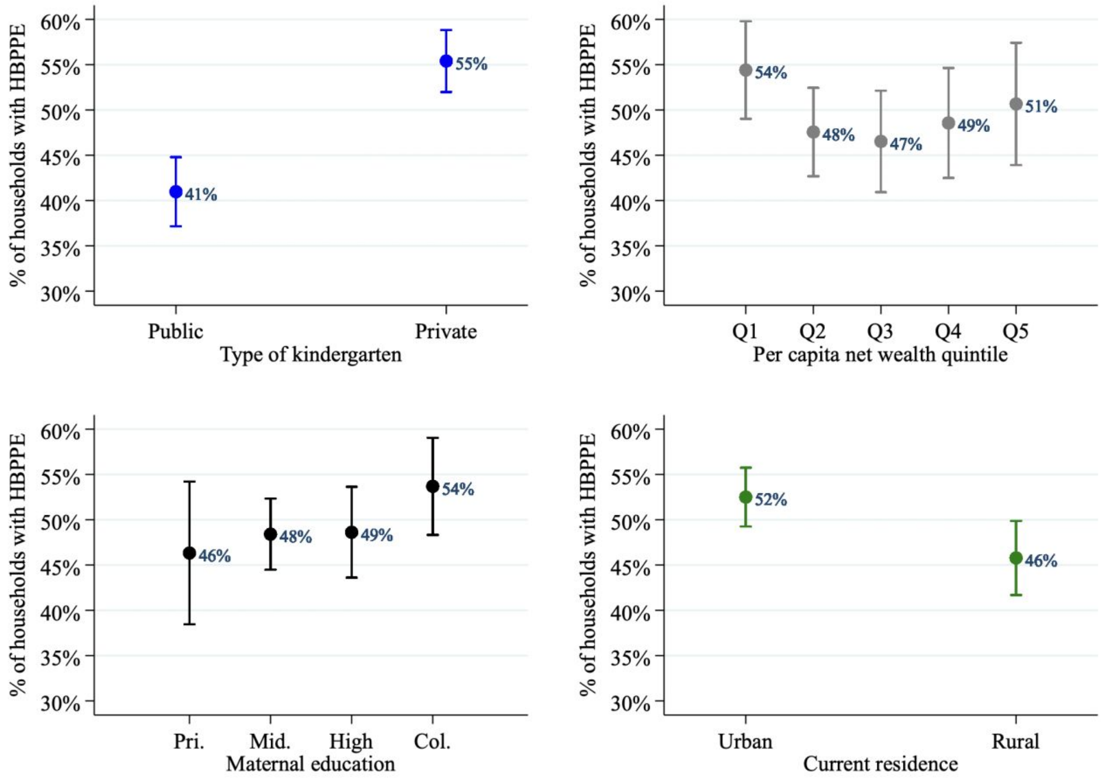

Notes: "Pri.", "Mid.", "High", and "Col." refers to maternal schooling at the primary and below, middle, high, and college and above levels, respectively.

For Review Only

Appendix

Chapter 1 Sampling Process of the China Household Finance Survey (CHFS)
Chapter 2 Constructing variables on socioecnomic charisteristics
Chapter 3 Testing the statistical significance of inequalities in PPE spending and HBPPE using Cumming and Finch's "rule of eye"
Chapter 4. Calculating provincial-level disparities in PPE spending and % of households with HBPPE
Figure A1. Government education spending on preschool education
Figure A2. Preschool enrollment and gross enrollment rate
Figure A3. Geographical distribution of the sampled counties
Figure A4. Average level of PPE spending per child across 29 provinces
Figure A5. Level and inequalities of PPE spending per child excluding out-school expenditure
Figure A6. Level of % of households with HBPPE (threshold=10%) across 29 provinces
Figure A7. Level of % of households with HBPPE (threshold=20%) across 29 provinces
Figure A8. Level and inequalities of % of households with HBPPE (thresehold=10%) by type of kindergarten, maternal education, household net wealth quintile, and current residential area
Figure A9. Level and inequalities of % of households with HBPPE (thresehold=20%) by type of kindergarten, maternal education, household net wealth quintile, and current residential area
Figure A10. Level and inequalities of % of households with HBPPE (threshold=7%) by excluding out-school spending from PPE spending
Figure A11. Level and inequalities of % of households with HBPPE (threshold=10%) by excluding out-school spending from PPE spending
Figure A12. Level and inequalities of % of households with HBPPE (threshold=20%) by excluding out-school spending from PPE spending
Table A1. Average level of PPE spending per child across 29 provinces and weighted mean difference of the mean between the provincial level and the national level
Table A2. Significance testing of inequalities in PPE spending per child using Cumming and Finch's "rule of eye"
Table A3. Level of % of households with HBPPE (threshold=7%) across 29 provinces and weighted mean difference of the mean between the provincial level and the national level
Table A4. Level of % of households with HBPPE (threshold=10%) across 29 provinces and weighted mean difference of the mean between the provincial level and the national level
Table A5. Level of % of households with HBPPE (threshold=20%) across 29 provinces and weighted mean difference of the mean between the provincial level and the national level
Table A6. Significance testing of inequalities in HBPPE (threshold=7%) using Cumming and Finch's "rule of eye"
Table A7. Significance testing of inequalities in HBPPE (threshold=10%) using Cumming and Finch's "rule of eye"
Table A8. Significance testing of inequalities in HBPPE (threshold=20%) using Cumming and Finch's "rule of eye"

Confidential: For Review Only

Chapter 1 Sampling Process of the China Household Finance Survey (CHFS)

The CHFS survey employs a stratified three-stage probability proportion-to-size random sample design. The primary sampling units include 2,585 counties/provincial cities in China. Tibet, Xinjiang, Hong Kong, Macao, and Chinese Taipei are not included in the primary sampling stage. The second stage of sampling involves selecting residential committees/villages from the counties/cities. The last stage involves selecting households from the residential committees/villages. A household unit is defined as all members of a housing unit related by blood, marriage, or some other legal arrangement; or two or more persons use incomes to make joint expenditures, or a single person living with others but is financially independent.

The CHFS is nationally and provincially representative survey conducted by the Southwestern University of Finance and Economics in China.[1] The CHFS collects rich information on household assets, income, wealth, and consumption. It also collects socioeconomic and demographic information on the sampled households and their members, including residential areas, household members' ages, gender, education levels, marital status, etc. The CHFS data are publicly available and have been widely used in the fields of economics, sociology, and education. By the end of November 2021, more than 35,000 researchers and 3,000 research institutions had registered and applied for the dataset, and the related studies have been published in top peer-reviewed journals.[2] Since 2017, the CHFS survey collaborated with the China Institute for Educational Finance Research and included a module on household spending on young children's care and education. The module includes questions on young children's schooling status, types of schooling, and household spending on various items that are related to preprimary attendance.

The CHFS 2019 covers 29 provinces (excluding Tibet, Xinjiang, Inner Mongolia, Hong Kong, and Macau), 345 cities/districts/counties, 34,643 households, and 107,008 individuals. Our focus was on household spending related to attending kindergarten. In China, kindergarten is usually offered to children 3-6 years old, but children under age 3 and over age 6 may also attend. In this study, we focused on children attending kindergarten, including those under age 3 or over age 6 who still attended kindergarten (18.2%). The 2019 data includes 4,085 young children aged 3-6 years old and 2,797 of these children attended kindergartens, yielding a 68.5% net enrollment rate.[3] The number of children attending kindergarten in all age groups is 3,415, yielding 83.6% of the gross enrollment rate, very close to the governmental estimate (83.4%).[4] Among households in this dataset, 42 (0.18%) didn't report spending information on PPE, and two reported PPE spending exceeding total household expenditures, both of which groups were excluded. The final sample includes 3,371 young children attending kindergartens, from 3,111 households (9% of the total sample), who reported available PPE expenditure data.

References

1. Gan, L., Yin, Z., Jia, N., Xu, S., & Ma, S. (2013). Data you need to know about China, Springer.
2. Survey and Research Center for China Household Finance, at https://chfs.swufe.edu.cn/

Confidential: For Review Only

3. The net enrolment rate (NER) is the number of students of the age of preprimary education (age 3-6) that are enrolled in that level of education, expressed as a percentage of the total population in that age group.
4. The gross enrollment rate (GER) is the total enrollment in preprimary education, regardless of age, expressed as a percentage of the population in the official age group (age 3-6) corresponding to this level of education. The GER can exceed 100% because of early or late entry and/or grade repetition.

Chapter 2 Constructing variables on socioecnomic charisteristics

Household economic status. In this study, we used per capita household net wealth to measure household economic status. Household net wealth was derived by calculating the difference between a household's assets and its debts. Household total assets include financial assets (e.g., cash, deposit balances, social security, stock/bond value, etc.) and non-financial assets (e.g., agricultural capital, industrial capital, commercial capital, housing, vehicles, and durable goods). Total household debts include financial asset debts, agricultural debts, business debts, housing debts, vehicle debts, other non-financial asset debts, educational debts, credit card debts, medical-care debts, and other debts. In this study, we divided households into five quintiles according to their per capita net wealth. We examined the difference in % of households with HBPPE across the five wealth quintiles.

Maternal schooling. We divided maternal schooling into four categories: primary school or below, middle school, high school, and college or above. About 12% of households in the sample of the study did not report the mothers' education; we replaced the missing data with the predicted values of mother's education by regressing mothers' education on fathers' education (ordered logit regression coefficient = 1.217, $p < 0.001$; correlation coefficient = 0.746) or household heads' education (ordered logit regression coefficient = 1.393, $p < 0.001$; correlation coefficient = 0.632).

Residential area. Because the urban-rural divide is still one of the main structural problems facing the schooling system in China, we also examined the disparities between urban and rural residential areas.

Type of kindergarten. Kindergartens in China can be categorized into public and private types, as discussed above. Households need to pay higher tuition fees to attend private kindergartens than public kindergartens. We assessed the disparities in the percentages of households with HBPPE by kindergarten type.

Chapter 3 Testing the statistical significance of inequalities in PPE spending and HBPPE using Cumming and Finch's "rule of eye"

To test whether inequalities in PPE spending or HBPPE significantly existed between the two sub-groups (e.g. male vs female), we compared the 95% confidence intervals (CIs) of the two estimates using Cumming and Finch's "rule of eye" for comparing two independent means.[1] We calculated three components that were required to implement the "rule of thumb": marginal

Confidential: For Review Only

errors, overlap of the 95% CIs of the two estimates, and proportion overlap. (1) Marginal error refers to the absolute difference between the mean and its lower or higher bound of a 95% CI. (2) An overlap of CIs refers to the difference between one estimate's higher bound and the other estimate's lower bound. (3) Proportion overlap is then calculated as the overlap of the 95% CIs between the two estimates divided by the average marginal errors between the two estimates.

Using Cumming and Finch's "rule of eye", we obtained proportion overlap using Equation PA1 or PA2 as follows, depending on the size of mean estimates for the two sub-groups. For example, when comparing the mean estimates for gender, if the mean estimate for male is lower than that for female, then

$$Proportion\ overlap = \frac{95\%\ CI\ upper\ bound_{male} - 95\%\ CI\ lower\ bound_{female}}{(Margin\ of\ error_{male} + Margin\ of\ error_{female})/2} \qquad \textbf{(PA1)}$$

Or, if the mean estimate for male is higher than that for female, then

$$Proportion\ overlap = \frac{95\%\ CI\ upper\ bound_{female} - 95\%\ CI\ lower\ bound_{male}}{(Margin\ of\ error_{male} + Margin\ of\ error_{female})/2} \qquad \textbf{(PA2)}$$

According to the "rule of eye", when sample sizes of two samples are both at least 10, and the margins of error between the two estimates do not differ by more than a factor of two, a proportion overlap less than 0.5 indicates a significant statistical relationship at the 0.05 level (i.e. $p<0.05$).

References

1. Cumming, G., & Finch, S. (2005). Inference by eye: Confidence intervals and how to read pictures of data. *American Psychologist*, 60(2),170-180.

Chapter 4. Calculating provincial-level disparities in PPE spending and % of households wth HBPPE

Considering the large variation in economic development and geographical situations across the provinces, we further assessed the provincial-level disparities in the level of PPE spending per child and % of households with HBPPE across the 29 provinces. The information will help the central government to identify the provinces with high percentages of households with HBPPE. Provincial-level inequalities were estimated with the weighted mean from the national mean, which was calculated as the values of the mean difference between each province and the national average, weighted by each province's population share ($=\frac{pop_i \times (r_i - r)}{pop}$), where *pop* is the national population size, pop_i is the population size of province *i*, *r* is the national mean, r_i is the mean of province *i*.). The measures took zero as the reference, with positive values indicating provincial means higher than the national average, and negative values indicating provincial means lower than the national average.

Figure A1. Government education spending on preschool education (Source: China Educational

Confidential: For Review Only

Finance Statistical Yearbook)

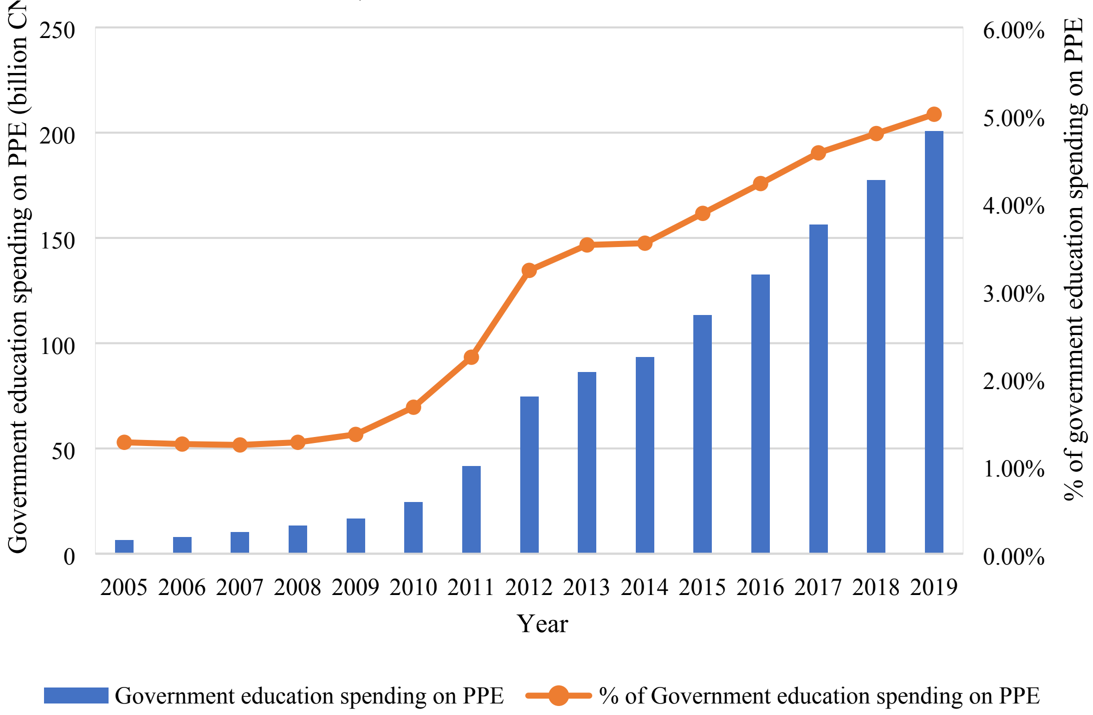

Figure A2. Preschool enrollment and gross enrollment rate (Source: China Education Statistical Yearbook)

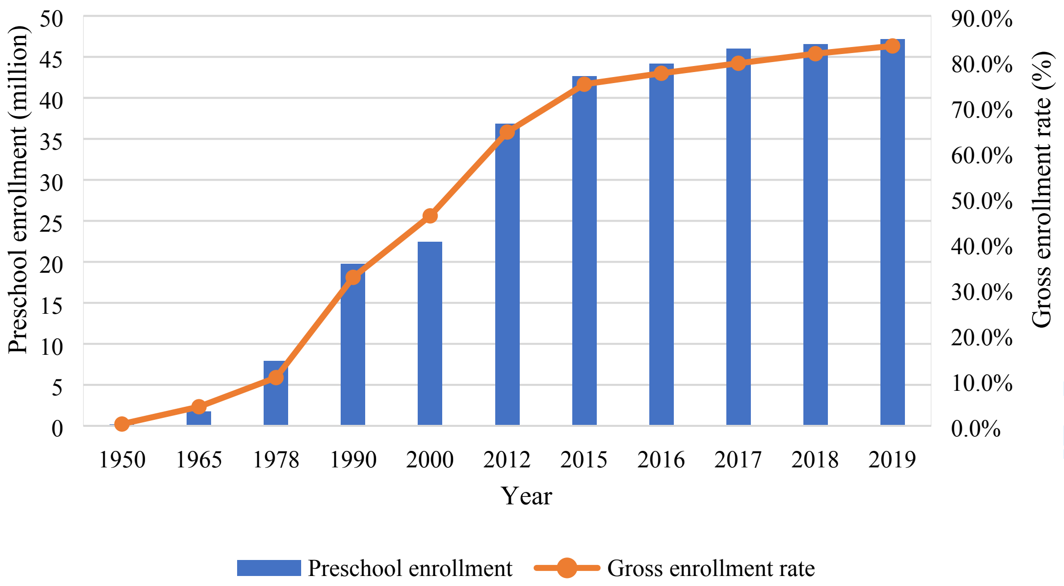

Figure A3. Geographical distribution of the sampled counties

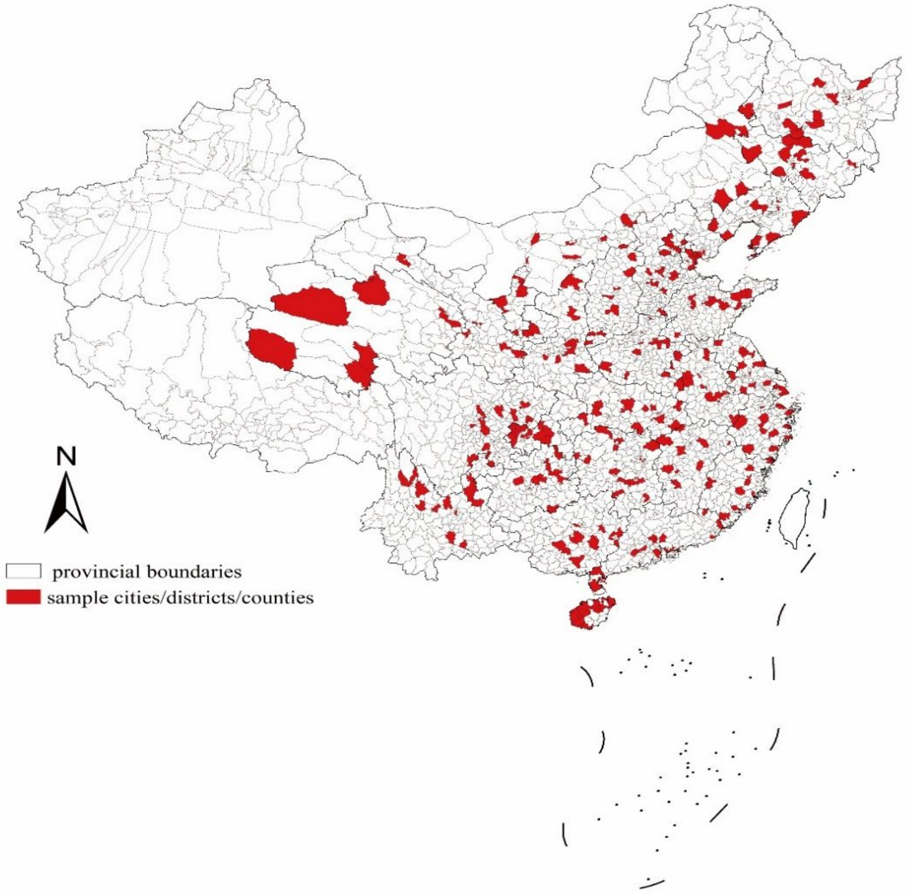

For peer review only

Figure A4. Average level of PPE spending per child across 29 provinces

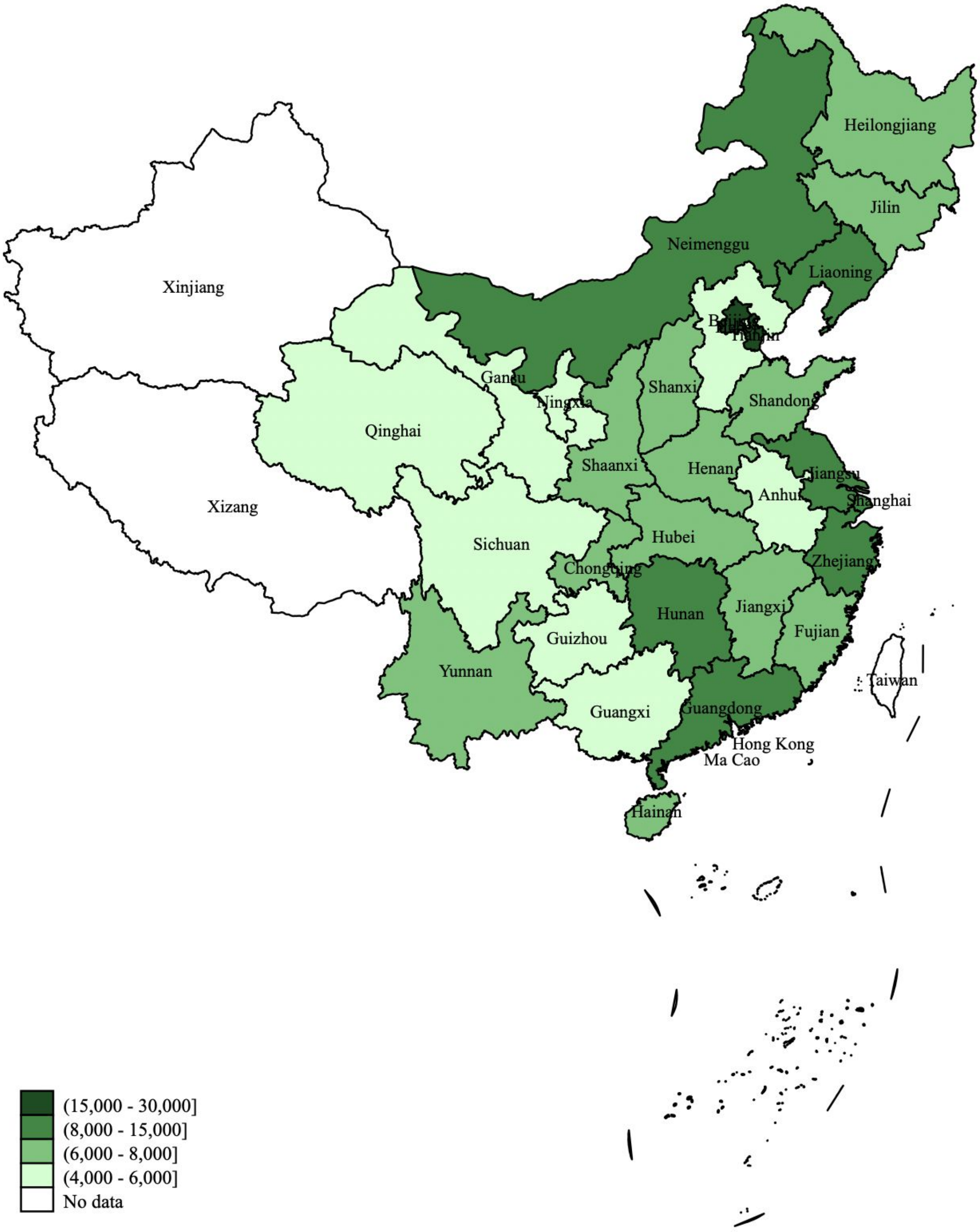

Figure A5. Level and inequalities of PPE spending per child excluding out-school expenditure

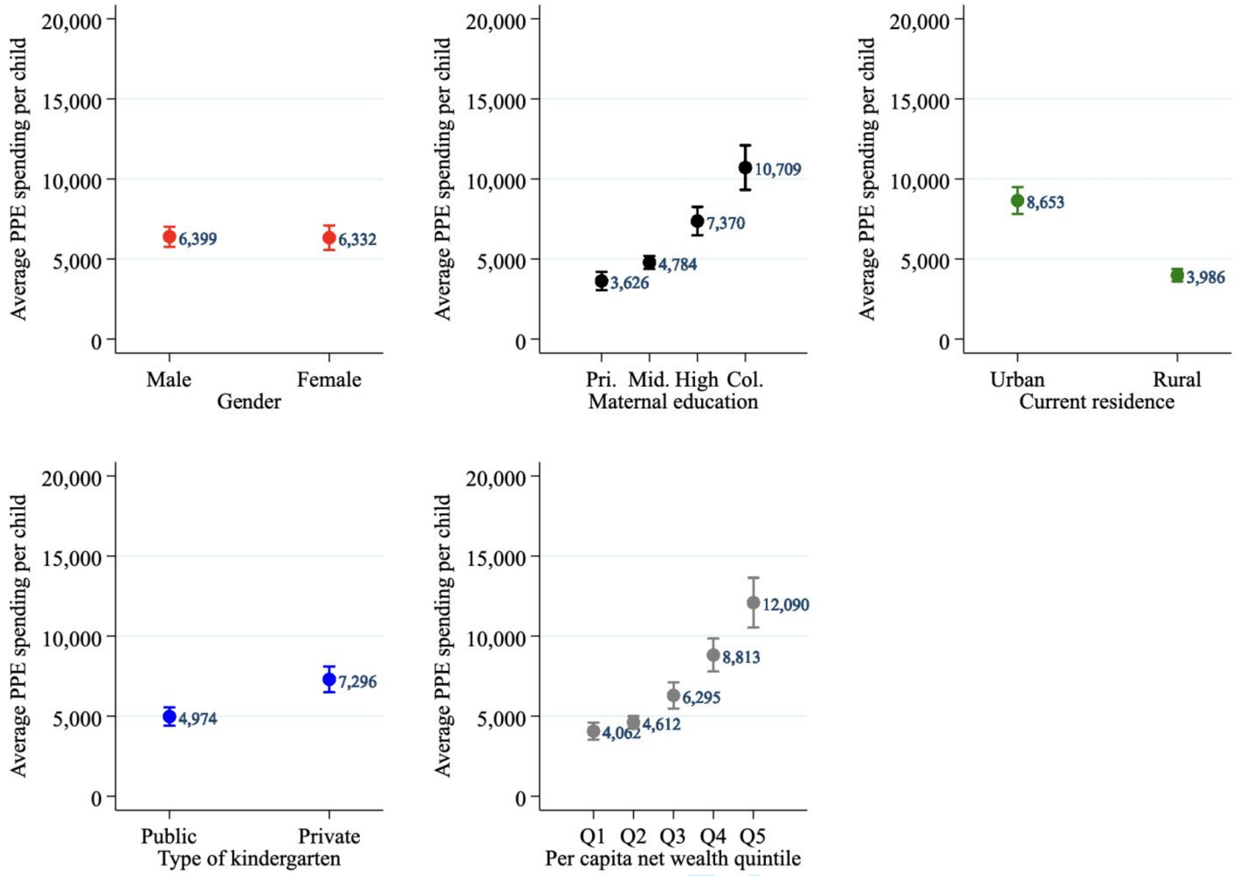

Notes: “Pri.”, “Mid.”, “High”, and “Col.” refers to maternal schooling at the primary and below, middle, high, and college and above levels, respectively.

Review Only

Figure A6. Level of % of households with HBPPE (threshold=10%) across 29 provinces

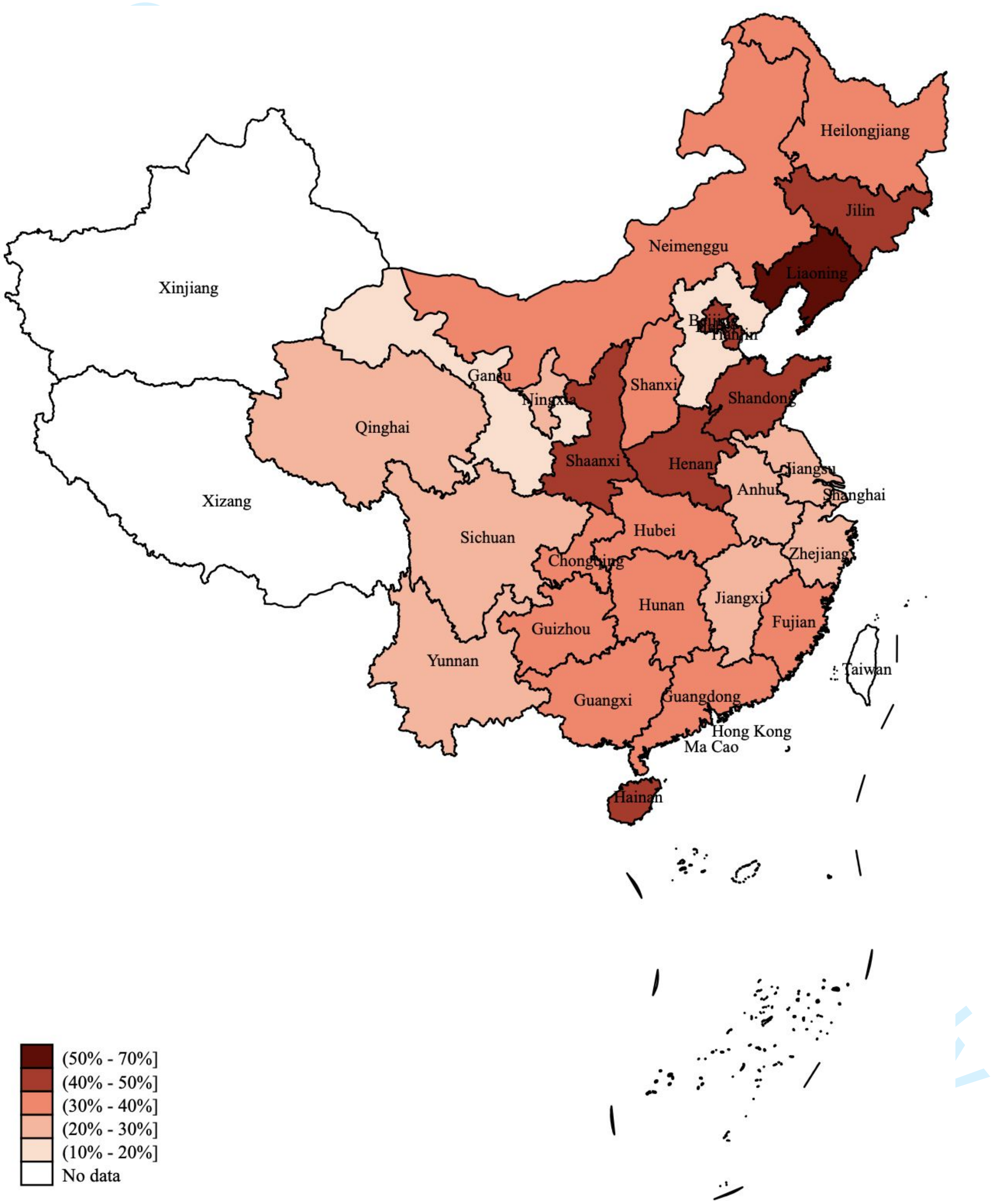

Figure A7. Level of % of households with HBPPE (threshold=20%) across 29 provinces

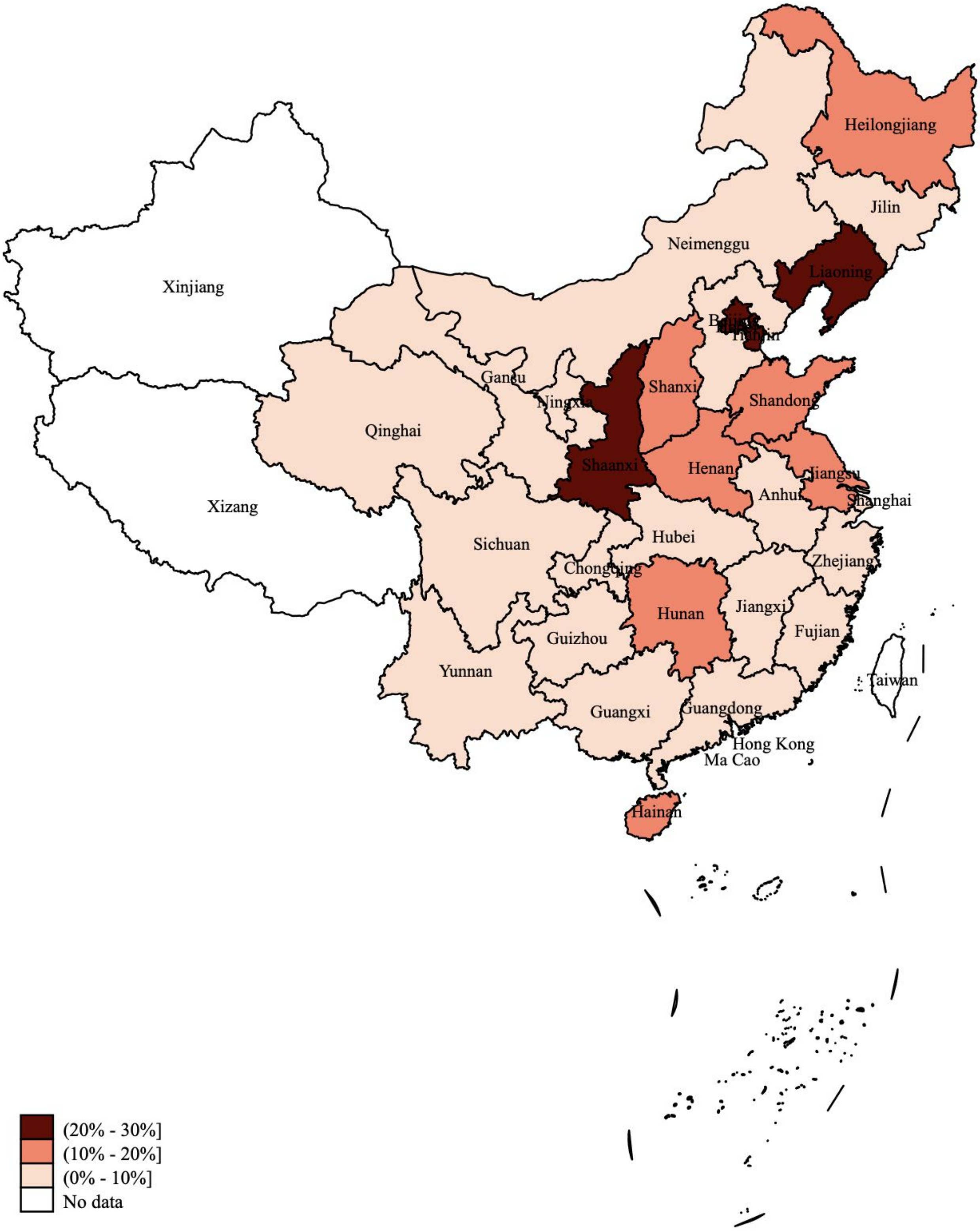

Figure A8. Level and inequalities of % of households with HBPPE (thresehold=10%) by type of kindergarten, maternal education, household net wealth quintile, and current residential area

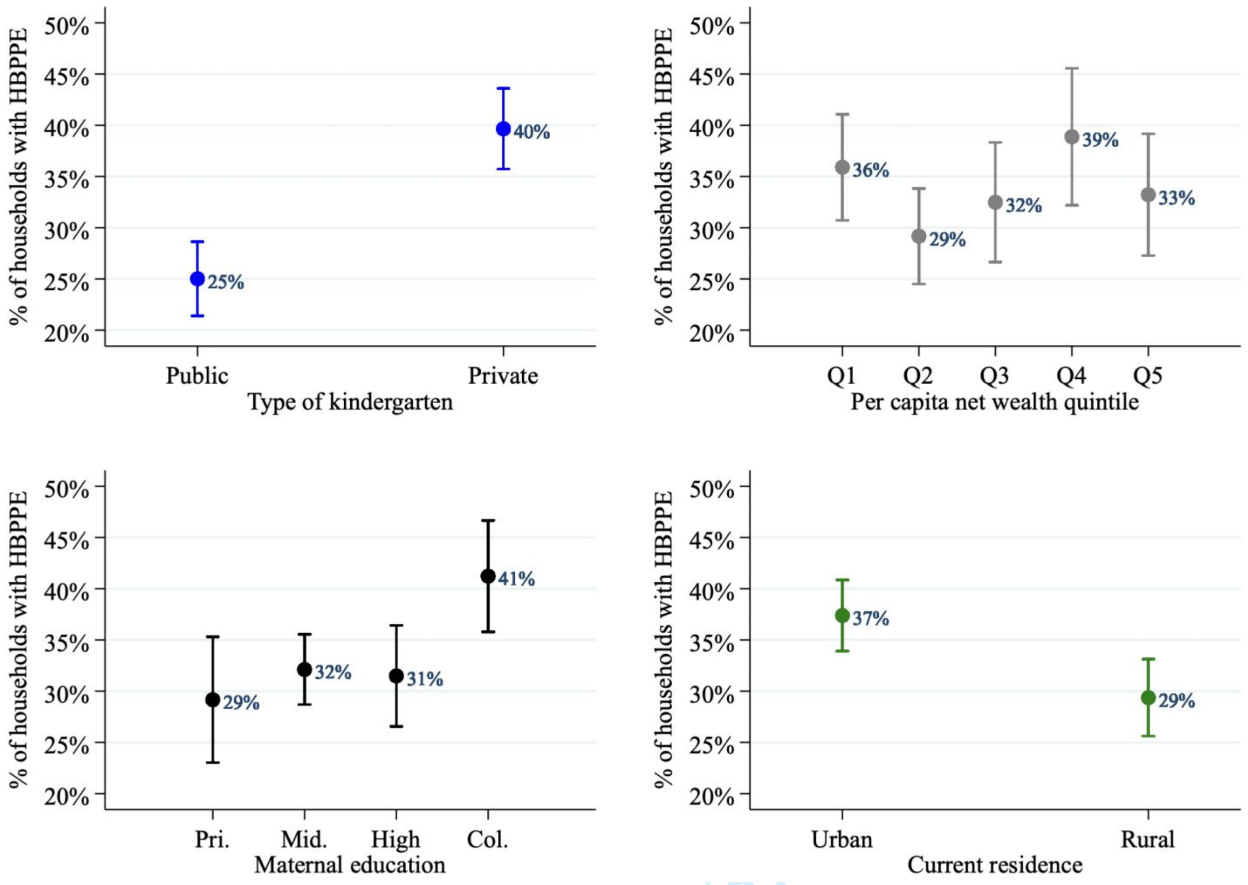

Notes: "Pri.", "Mid.", "High", and "Col." refers to maternal schooling at the primary and below, middle, high, and college and above levels, respectively.

Figure A9. Level and inequalities of % of households with HBPPE (thresehold=20%) by type of kindergarten, maternal education, household net wealth quintile, and current residential area

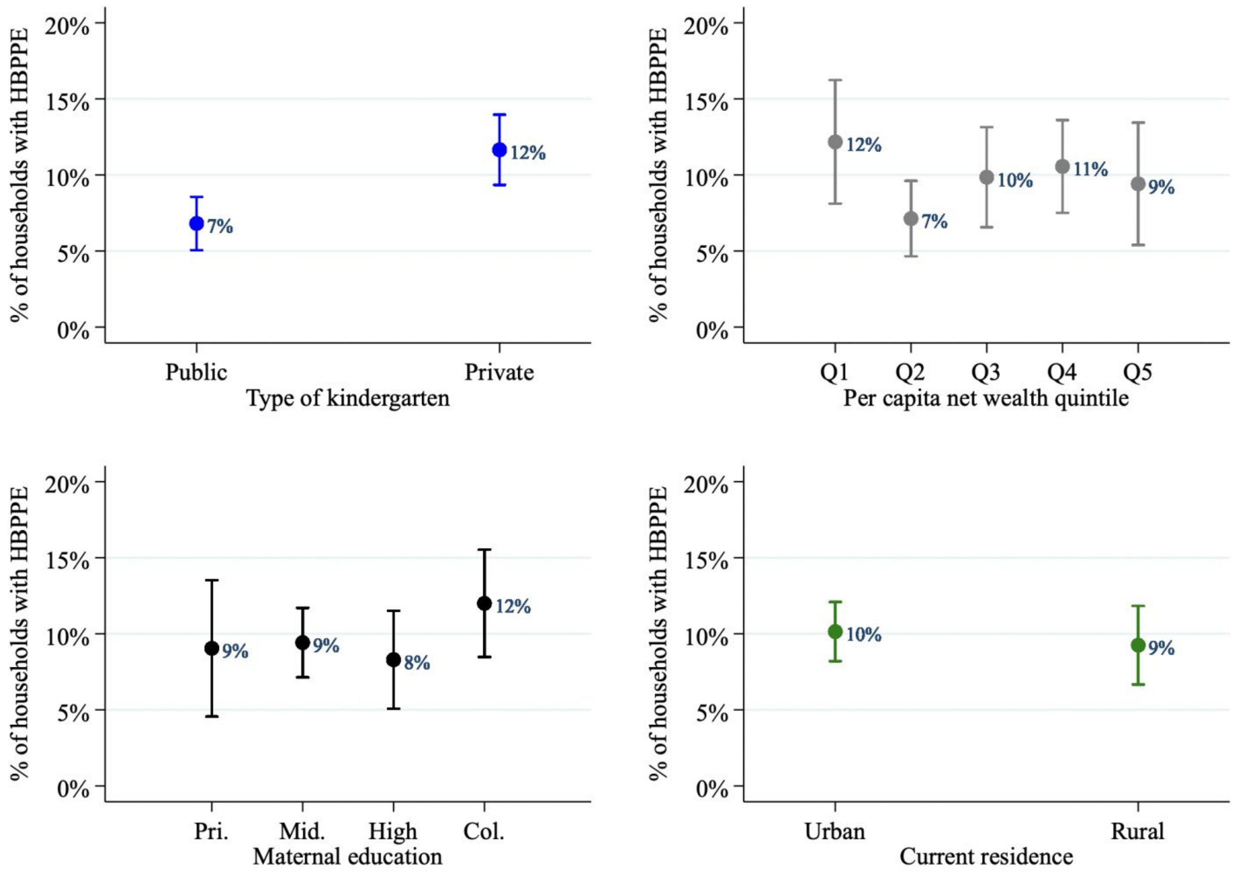

Notes: "Pri.", "Mid.", "High", and "Col." refers to maternal schooling at the primary and below, middle, high, and college and above levels, respectively.

For peer review Only

Figure A10. Level and inequalities of % of households with HBPPE (thresehold=7%) by excluding out-school spending from PPE spending

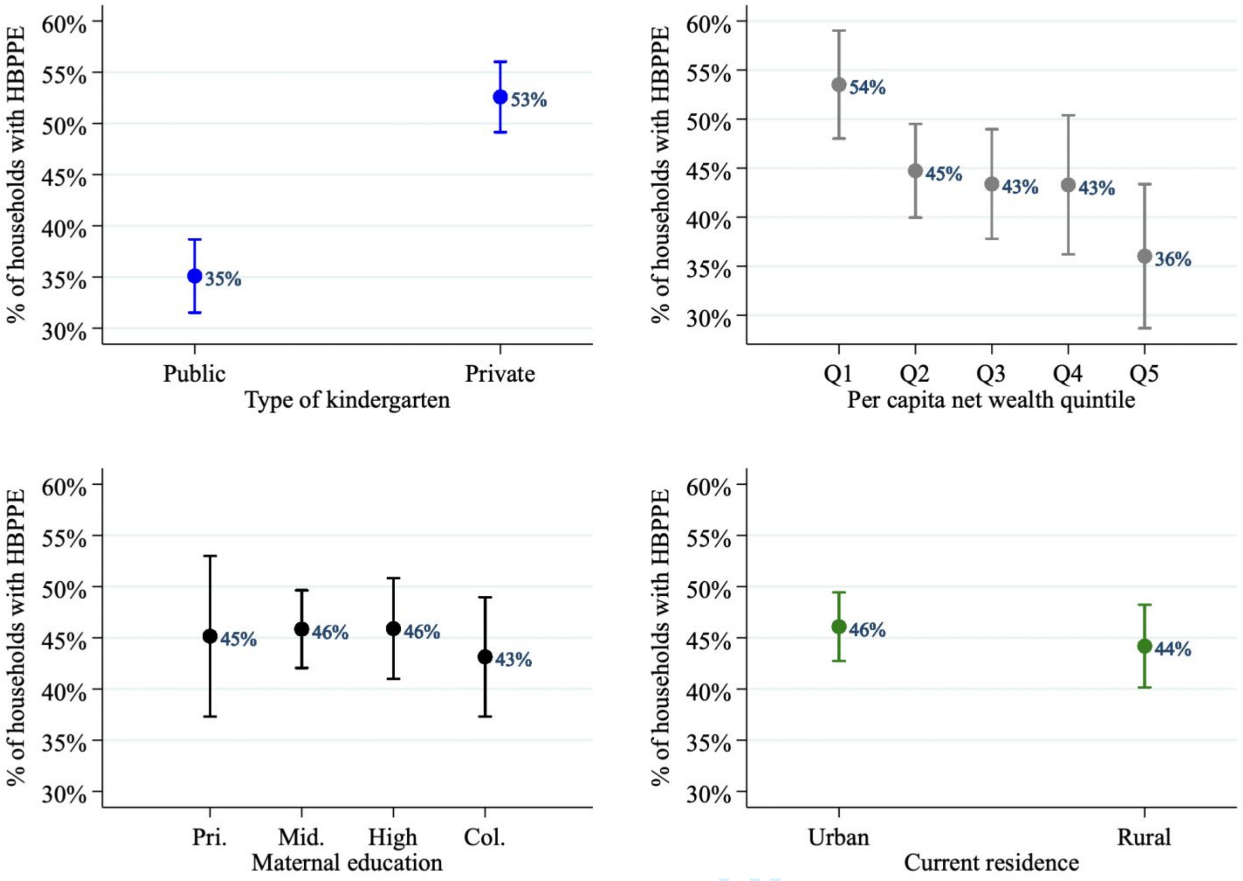

Notes: "Pri.", "Mid.", "High", and "Col." refers to maternal schooling at the primary and below, middle, high, and college and above levels, respectively.

Figure A11. Level and inequalities of % of households with HBPPE (thresehold=10%) by excluding out-school spending from PPE spending

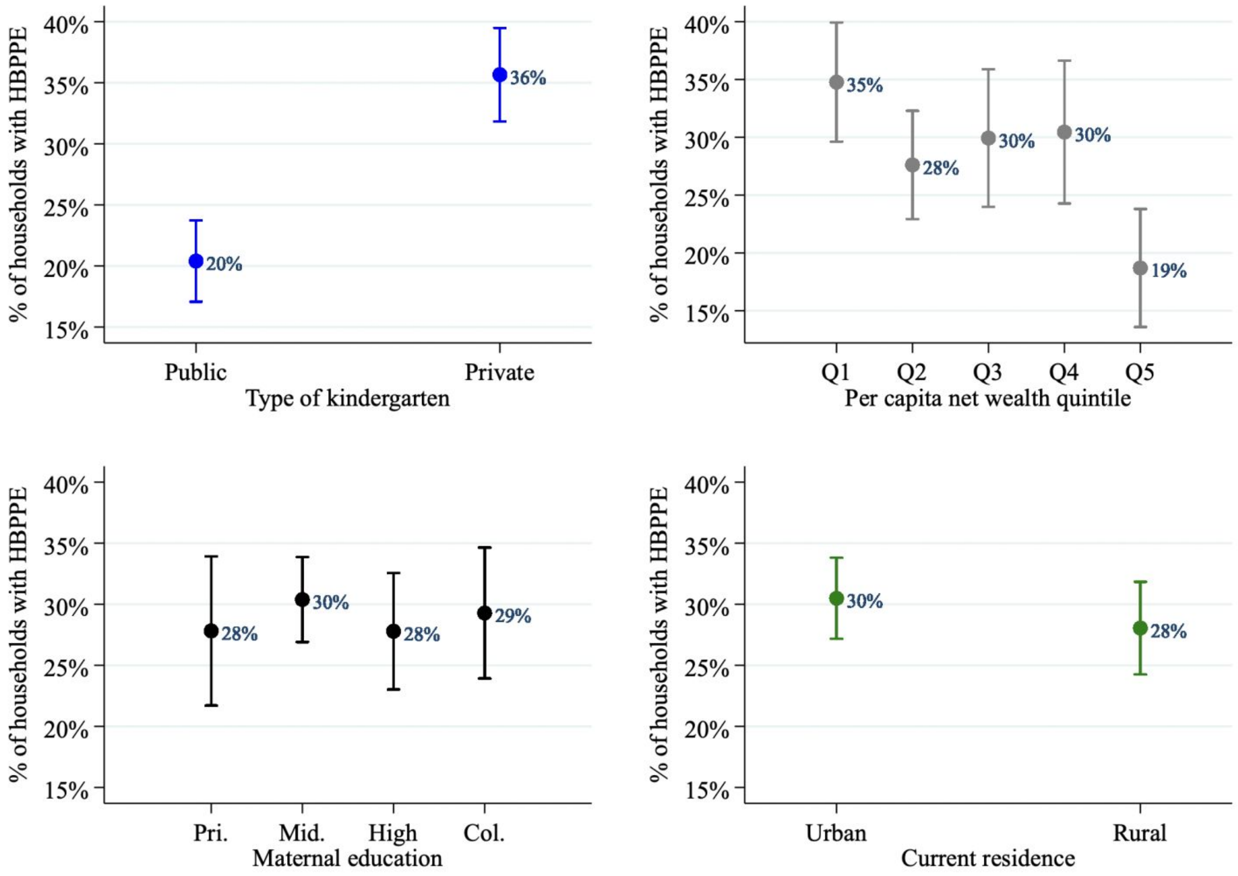

Notes: “Pri.”, “Mid.”, “High”, and “Col.” refers to maternal schooling at the primary and below, middle, high, and college and above levels, respectively.

review Only

Figure A12. Level and inequalities of % of households with HBPPE (thresehold=20%) by excluding out-school spending from PPE spending

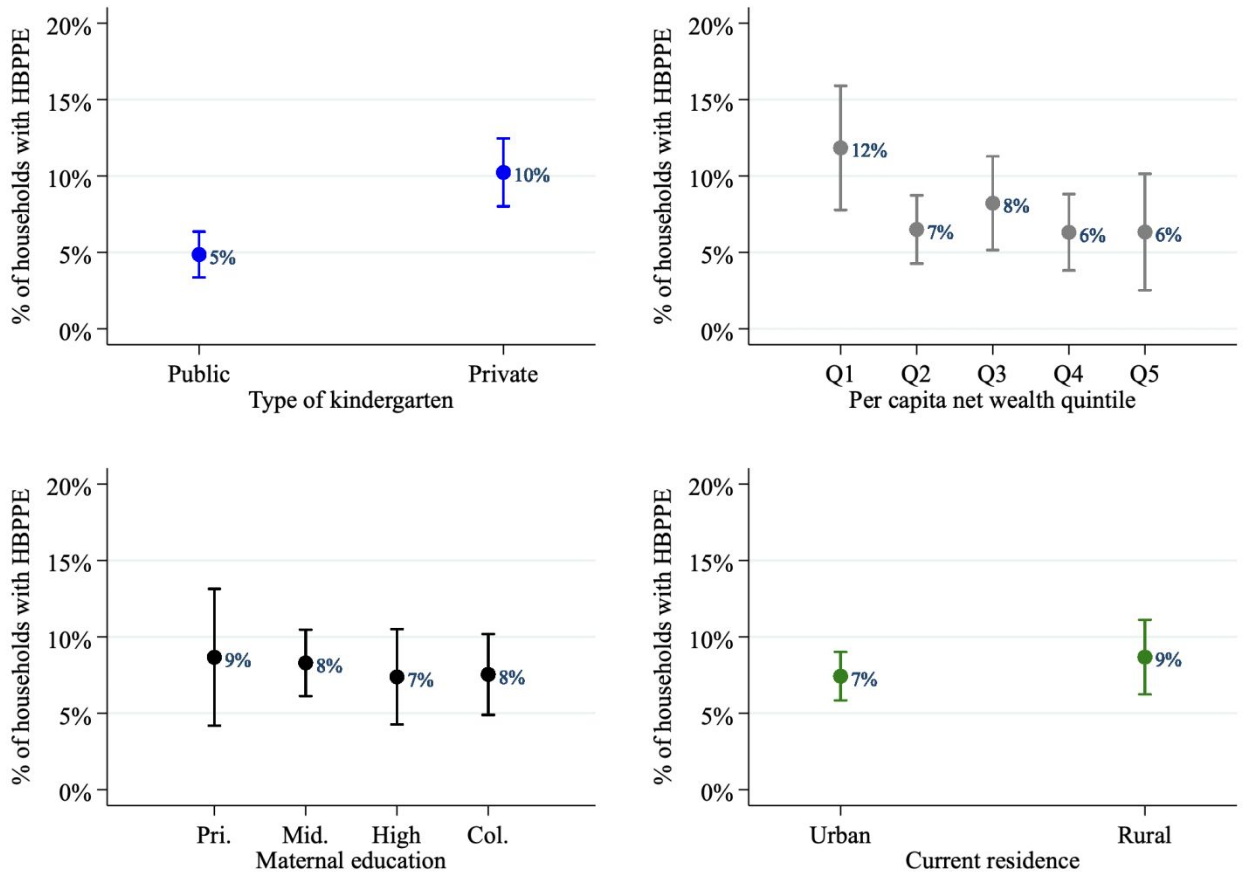

Notes: “Pri.”, “Mid.”, “High”, and “Col.” refers to maternal schooling at the primary and below, middle, high, and college and above levels, respectively.

eview Only

Table A1. Average level of PPE spending per child across 29 provinces and weighted mean difference of the mean between the provincial level and the national level

Province	PPE spending at the provincial level	Weighted mean difference*
Beijing	23,348	246
Tianjin	19,487	135
Liaoning	13,417	189
Zhejiang	11,057	155
Shanghai	10,279	51
Neimenggu	10,095	50
Guangdong	9,309	162
Jiangsu	8,163	47
Hunan	8,030	34
Henan	7,942	41
Fujian	7,877	15
Shaanxi	7,740	11
Chongqing	7,062	-6
Heilongjiang	6,980	-10
Hubei	6,964	-16
Shandong	6,852	-35
Jilin	6,850	-10
Hainan	6,614	-5
Jiangxi	6,202	-38
Shanxi	6,090	-33
Yunnan	6,004	-47
Ningxia	5,496	-9
Sichuan	5,456	-113
Anhui	4,869	-113
Guizhou	4,674	-69
Guangxi	4,536	-100
Gansu	4,506	-54
Hebei	4,246	-168
Qinghai	4,070	-14

*: The measures took zero as the reference, with positive values indicating provincial means higher than the national average, and negative values indicating provincial means lower than the national average.

Confidential: For Review Only

Table A2. Significance testing of inequalities in PPE spending per child using Cumming and Finch's "rule of eye"

	Mean	Lower bound	Higher bound	Margins of error	Proportion overlap			
Gender					vs female			
Male	7437.056	6631.678	8242.434	805.378	1.763			
Female	7238.254	6369.469	8107.039	868.785				
Private school					vs private			
Public	6193.239	5475.135	6911.343	718.104	**-0.244**			
Private	8075.494	7116.165	9034.824	959.330				
Maternal education					vs middle	vs high	vs college	
Primary	3756.339	3163.960	4348.719	592.379	**-0.615**	**-3.341**	**-6.995**	
Middle	5138.580	4673.767	5603.393	464.813	.	**-1.940**	**-6.229**	
High	8068.083	7045.833	9090.333	1022.250	.	.	**-2.314**	
College	13824.870	12178.490	15471.250	1646.381				
Per capita net wealth quintile					vs Q2	vs Q3	vs Q4	vs Q5
Q1	4182.038	3628.280	4735.797	553.759	0.683	**-1.678**	**-4.956**	**-7.985**
Q2	4813.727	4408.062	5219.393	405.666	.	**-1.117**	**-4.816**	**-8.074**
Q3	6812.270	5935.722	7688.817	876.548	.	.	**-1.435**	**-5.051**
Q4	10473.690	9218.501	11728.880	1255.188	.	.	.	**-1.995**
Q5	17007.120	14991.910	19022.340	2015.218				
Current residence					vs rural			
Urban	10433.780	9434.861	11432.700	998.918	**-7.059**			
Rural	4126.709	3733.122	4520.297	393.588				

Notes: we used Cumming and Finch's "rule of eye" to test the statistical significance of inequalities in PPE spending between the two sub-groups (i.e. whether significant difference existed between the two mean estimates). Details for the analytical methods were presented in **Appendix Panel A**. Numbers in bold indicates a proportion overlap that is less than 0.5; that is a significant statistical relationship at the 0.05 level (p<0.05)

Confidential: For Review Only

Table A3. Level of % of households with HBPPE (threshold=7%) across 29 provinces and weighted mean difference of the mean between the provincial level and the national level

Province	Level of % of households with HBPPE at the provincial level	Weighted mean difference
Liaoning	76.9%	0.0086
Shaanxi	67.0%	0.0049
Beijing	64.5%	0.0023
Neimenggu	62.5%	0.0024
Henan	61.2%	0.0082
Hainan	60.0%	0.0007
Tianjin	59.9%	0.0012
Hubei	58.5%	0.0039
Heilongjiang	54.4%	0.0014
Hunan	53.8%	0.0022
Jilin	52.7%	0.0007
Guangxi	52.3%	0.0011
Shandong	51.5%	0.0016
Shanxi	49.3%	0.0000
Fujian	48.7%	-0.0002
Guangdong	47.7%	-0.0013
Jiangsu	46.6%	-0.0016
Sichuan	44.4%	-0.0029
Qinghai	43.9%	-0.0002
Jiangxi	42.2%	-0.0023
Chongqing	41.1%	-0.0018
Guizhou	40.9%	-0.0022
Yunnan	40.5%	-0.0030
Ningxia	40.5%	-0.0004
Anhui	39.9%	-0.0043
Shanghai	39.4%	-0.0017
Zhejiang	36.4%	-0.0054
Hebei	35.8%	-0.0073
Gansu	33.1%	-0.0031

*: The measures took zero as the reference, with positive values indicating provincial means higher than the national average, and negative values indicating provincial means lower than the national average.

Confidential: For Review Only

Table A4. Level of % of households with HBPPE (threshold=10%) across 29 provinces and weighted mean difference of the mean between the provincial level and the national level

Province	Level of % of households with HBPPE at the provincial level	Weighted mean difference
Liaoning	63.9%	0.0094
Tianjin	49.5%	0.0018
Beijing	49.1%	0.0024
Shaanxi	48.8%	0.0042
Jilin	47.0%	0.0026
Henan	46.1%	0.0086
Hainan	45.2%	0.0008
Shandong	42.5%	0.0064
Heilongjiang	39.2%	0.0015
Chongqing	36.5%	0.0007
Neimenggu	35.1%	0.0003
Hunan	34.2%	0.0003
Guangdong	33.4%	-0.0001
Fujian	32.0%	-0.0004
Hubei	31.9%	-0.0007
Guangxi	31.6%	-0.0007
Guizhou	30.9%	-0.0007
Shanxi	30.3%	-0.0009
Ningxia	29.7%	-0.0002
Jiangsu	29.3%	-0.0024
Yunnan	27.9%	-0.0020
Sichuan	27.6%	-0.0035
Anhui	25.9%	-0.0035
Shanghai	25.5%	-0.0014
Qinghai	23.6%	-0.0004
Zhejiang	23.1%	-0.0044
Jiangxi	22.7%	-0.0036
Hebei	19.7%	-0.0075
Gansu	19.0%	-0.0028

*: The measures took zero as the reference, with positive values indicating provincial means higher than the national average, and negative values indicating provincial means lower than the national average.

Confidential: For Review Only

Table A5. Level of % of households with HBPPE (threshold=20%) across 29 provinces and weighted mean difference of the mean between the provincial level and the national level

Province	Level of % of households with HBPPE at the provincial level	Weighted mean difference
Liaoning	27.8%	0.0056
Tianjin	23.6%	0.0015
Shaanxi	22.9%	0.0036
Beijing	20.1%	0.0016
Shandong	18.9%	0.0066
Hainan	17.3%	0.0005
Heilongjiang	16.9%	0.0019
Shanxi	12.8%	0.0008
Henan	12.6%	0.0020
Hunan	11.1%	0.0007
Jiangsu	10.2%	0.0003
Yunnan	10.0%	0.0001
Qinghai	9.9%	0.0000
Guangdong	9.3%	-0.0004
Guizhou	8.8%	-0.0002
Jilin	8.4%	-0.0003
Zhejiang	8.3%	-0.0006
Chongqing	6.8%	-0.0006
Neimenggu	6.7%	-0.0005
Hubei	6.4%	-0.0014
Shanghai	5.9%	-0.0007
Guangxi	5.2%	-0.0016
Gansu	5.1%	-0.0009
Ningxia	4.8%	-0.0002
Fujian	4.7%	-0.0014
Sichuan	4.5%	-0.0031
Jiangxi	4.4%	-0.0018
Hebei	4.0%	-0.0031
Anhui	2.9%	-0.0031

*: The measures took zero as the reference, with positive values indicating provincial means higher than the national average, and negative values indicating provincial means lower than the national average.

Confidential: For Review Only

Table A6. Significance testing of inequalities in HBPPE (threshold=7%) using Cumming and Finch's "rule of eye"

	Mean	Lower bound	Higher bound	Margins of error	Proportion overlap			
Private school					vs private			
Public	0.410	0.372	0.448	0.038	**-1.970**			
Private	0.554	0.520	0.588	0.034				
Maternal education					vs middle	vs high	vs college	
Primary	0.463	0.384	0.542	0.079	1.648	1.645	0.890	
Middle	0.484	0.445	0.523	0.039	.	1.952	0.864	
High	0.486	0.436	0.536	0.050	.	.	1.024	
College	0.537	0.483	0.590	0.054				
Per capita net wealth quintile					vs Q2	vs Q3	vs Q4	vs Q5
Q1	0.544	0.490	0.598	0.054	0.667	0.566	0.980	1.383
Q2	0.476	0.427	0.524	0.049	.	1.803	1.817	1.467
Q3	0.465	0.409	0.521	0.056	.	.	1.651	1.330
Q4	0.486	0.425	0.546	0.061	.	.	.	1.673
Q5	0.507	0.439	0.574	0.068				
Current residence					vs rural			
Urban	0.525	0.493	0.557	0.032	**0.168**			
Rural	0.458	0.417	0.499	0.041				

Notes: we used Cumming and Finch's "rule of eye" to test the statistical significance of inequalities in HBPPE between the two sub-groups (i.e. whether significant difference existed between the two mean estimates). Numbers in bold indicates a proportion overlap that is less than 0.5; that is a significant statistical relationship at the 0.05 level (p<0.05)

Confidential: For Review Only

Table A7. Significance testing of inequalities in HBPPE (threshold=10%) using Cumming and Finch's "rule of eye"

	Mean	Lower bound	Higher bound	Margins of error	Proportion overlap			
Private school					vs private			
Public	0.250	0.214	0.286	0.036	**-1.873**			
Private	0.396	0.357	0.436	0.039				
Maternal education					vs middle	vs high	vs college	
Primary	0.292	0.230	0.353	0.062	1.384	1.580	**-0.082**	
Middle	0.321	0.287	0.356	0.034	.	1.851	**-0.053**	
High	0.315	0.266	0.364	0.049	.	.	**0.122**	
College	0.412	0.358	0.467	0.054				
Per capita net wealth quintile					vs Q2	vs Q3	vs Q4	vs Q5
Q1	0.359	0.307	0.411	0.052	0.631	1.380	1.498	1.518
Q2	0.292	0.245	0.338	0.047	.	1.367	**0.288**	1.235
Q3	0.325	0.267	0.383	0.058	.	.	0.979	1.875
Q4	0.389	0.322	0.456	0.067	.	.	.	1.104
Q5	0.332	0.273	0.392	0.059				
Current residence					vs rural			
Urban	0.374	0.339	0.409	0.035	**-0.216**			
Rural	0.294	0.256	0.331	0.038				

Notes: Numbers in bold indicates a proportion overlap that is less than 0.5; that is a significant statistical relationship at the 0.05 level (p<0.05)

Confidential: For Review Only

Table A8. Significance testing of inequalities in HBPPE (threshold=20%) using Cumming and Finch's "rule of eye"

	Mean	Lower bound	Higher bound	Margins of error	Proportion overlap			
Private school					vs private			
Public	0.068	0.051	0.086	0.018	**-0.377**			
Private	0.116	0.093	0.140	0.023				
Maternal education					vs middle	vs high	vs college	
Primary	0.090	0.046	0.135	0.045	1.888	1.806	1.261	
Middle	0.094	0.071	0.117	0.023	.	1.591	1.112	
High	0.083	0.051	0.115	0.032	.	.	0.902	
College	0.120	0.085	0.155	0.035				
Per capita net wealth quintile					vs Q2	vs Q3	vs Q4	vs Q5
Q1	0.122	0.081	0.162	0.041	**0.459**	1.368	1.547	1.318
Q2	0.071	0.047	0.096	0.025	.	1.058	0.760	1.298
Q3	0.099	0.066	0.131	0.033	.	.	1.776	1.881
Q4	0.106	0.075	0.136	0.030	.	.	.	1.676
Q5	0.094	0.054	0.134	0.040				
Current residence					vs rural			
Urban	0.101	0.082	0.121	0.019	1.604			
Rural	0.092	0.067	0.118	0.026				

Notes: Numbers in bold indicates a proportion overlap that is less than 0.5; that is a significant statistical relationship at the 0.05 level ($p<0.05$)

Confidential: For Review Only

Confidential: For Review Only

24

BMJ Paediatrics Open

Assessing household financial burdens for preprimary education and associated socioeconomic inequalities – a case study in China

Journal:	*BMJ Paediatrics Open*
Manuscript ID	bmjpo-2023-001971.R1
Article Type:	Original research
Date Submitted by the Author:	31-May-2023
Complete List of Authors:	Wei, Yi; Peking University, China Institute for Educational Finance Research<br>Liu, Kai; Renmin University of China, School of Labor and Human Resources<br>Kang, Le; Nanjing University, Institute of Education<br>Behrman, Jere; University of Pennsylvania, Department of Economics<br>Richter, Linda; University of the Witwatersrand Johannesburg, DSI-NRF Centre of Excellence in Human Development<br>Stein, Alan; Oxford University, Department of Psychiatry<br>Song, Yingquan; Peking University, China Institute for Educational Finance Research<br>Lu, Chunling; Brigham and Women's Hospital, Division of Global Health Equity; Harvard Medical School, Department of Global Health and Social Medicine
Keywords:	Growth

SCHOLARONE™
Manuscripts

I, the Submitting Author has the right to grant and does grant on behalf of all authors of the Work (as defined in the below author licence), an exclusive licence and/or a non-exclusive licence for contributions from authors who are: i) UK Crown employees; ii) where BMJ has agreed a CC-BY licence shall apply, and/or iii) in accordance with the terms applicable for US Federal Government officers or employees acting as part of their official duties; on a worldwide, perpetual, irrevocable, royalty-free basis to BMJ Publishing Group Ltd ("BMJ") its licensees and where the relevant Journal is co-owned by BMJ to the co-owners of the Journal, to publish the Work in this journal and any other BMJ products and to exploit all rights, as set out in our licence.

The Submitting Author accepts and understands that any supply made under these terms is made by BMJ to the Submitting Author unless you are acting as an employee on behalf of your employer or a postgraduate student of an affiliated institution which is paying any applicable article publishing charge ("APC") for Open Access articles. Where the Submitting Author wishes to make the Work available on an Open Access basis (and intends to pay the relevant APC), the terms of reuse of such Open Access shall be governed by a Creative Commons licence – details of these licences and which Creative Commons licence will apply to this Work are set out in our licence referred to above.

Other than as permitted in any relevant BMJ Author's Self Archiving Policies, I confirm this Work has not been accepted for publication elsewhere, is not being considered for publication elsewhere and does not duplicate material already published. I confirm all authors consent to publication of this Work and authorise the granting of this licence.

Confidential: For Review Only

Confidential: For Review Only

Assessing household financial burdens for preprimary education and associated socioeconomic inequalities – a case study in China

Yi Wei, Kai Liu, Le Kang, Jere R. Behrman, Linda M. Richter, Alan Stein, Yingquan Song, Chunling Lu

Abstract

Background Providing young children with universal access to preprimary education (PPE) is considered a powerful tool for human-capital development and eliminating the intergenerational transmission of poverty. To remove household financial barrier for achieving universal PPE, this study proposed a measure to identify households incurring "heavy financial burdens from paying for PPE" (HBPPE), illustrated by a case study in China.

Methods Using nationally representative data in 2019, we estimated the percentage of households with HBPPE (spent 7% or more of their total annual expenditure) and associated socioecnomic inequaities. We also applied a three-level logit-regression model to investigate the factors associated with the probabilities of households incurring HBPPE.

Results Half of the sampled households spent 7% or more of their expenditures on PPE. Households in the lowest wealth quintile (54%) or households with children attending private PPE (55%) had higher percentages of HBPPE than households in other wealth quintiles (e.g., 51% in the highest wealth quintile) or households with children attending public kindergartens (41%). Logit-regression analysis shows that the poorest households and households with children attending private kindergarten were more likely to incur HBPPE than their counterparts.

Conclusion To ensure universal access to PPE in China, future policy should consider increasing the enrollment of children from low-income families in public kindergartens and increasing governmental investments in low-income households by subsidizing children attending PPE.

Keywords: Access to kindergarten; Preprimary education, Household financial burden; Socioeconomic inequality

WHAT IS ALREADY KNOWN ON THIS TOPIC

- Significant socioeconomic inequalities in preprimary education (PPE) attendance have been observed in many low- and middle-income countries (LMICs).
- Empirical evidence on household financial barriers to PPE in LMICs is very limited. In China, disadvantaged households (e.g., low-income, low maternal schooling) paid a larger share than their counterparts for sending children to preschool. Little, however, is known about how many households suffered from heavy financial burden from paying for PPE.

WHAT THIS STUDY ADDS

- This study proposed a measure to identify households incurring "heavy financial burdens from paying for PPE" (HBPPE), illustrated by a case study in China using nationally representative data.
- Findings showed that almost 50% of the sampled households incurred HBPPE (spent over 7% or more of their annual expenditures on PPE), with the poorest households and households in which children attended private kindergarten more likely to incur HBPPE than their counterparts.

HOW THIS STUDY MIGHT AFFECT RESEARCH, PRACTICE OR POLICY

- This study proposed a measure of HBPPE that could be used for similar research in understanding country progress in eliminating financial barrier to universal coverage of PPE.
- Findings of this study could inform policymakers and other stakeholders in China, specifically, on who suffered HBPPE (e.g., the poorest households or households with children attending private kindergarten) and what could be potentially effective policy instruments in promoting equal access to PPE.

1. Introduction

Preprimary education (PPE) has important influences on the development of cognitive and non-cognitive skills of children, especially for those from disadvantaged groups.[1] The high return on investment in PPE [2-5] makes it an important strategy for strengthening national human capital, as well as reducing inequality and the intergenerational transmission of poverty.[6-8] Quality PPE for all girls and boys has been included in the 2030 Sustainable Development Goals.[9]

Ensuring universal access to quality PPE requires sufficient investment and effective design of cost-sharing mechanisms between governments and households. In many low- and middle-income countries (LMICs), government financial support to PPE remains rather limited,[10-12] making it difficult for poor households to access PPE. In Nigeria, for example, less than 10% of the poorest young children attended PPE in 2010, compared to approximately 80% of the richest young Nigerian children.[13]

China has the second-largest child population in the world and has set a policy goal of universal coverage of PPE in 2010.[14] Since then, public expenditures on PPE have increased rapidly from CNY24.44 billion (1.67% of governmental educational spending) in 2010 to CNY 200.8 billion (5.01% of governmental educational spending) in 2019 (**Figure A1 in the appendix**).[15] The gross-enrollment rate for three-year PPE also grew rapidly from 46.1% in 2000 to 83.4% in 2019 (**Figure A2 in the appendix**).[15]

PPE in China is not free. Households have to pay tuition fees that vary greatly by type of kindergarten, public or private. Public kindergartens are essentially "club goods", which are the extension of the welfare system in the era of the planned economy, as a benefit provided by organizations to their "employees". These public kindergartens are not open to the public and have certain requirements for admission; for example, children of employees of governments, universities, or state-owned enterprises, etc. Children from more-advantaged groups are more likely to enter this kind of public kindergarten with relatively low fees and higher quality.[16,17]

Confidential: For Review Only

Currently, government spending on PPE mainly goes to these public kindergartens, especially the kindergartens run by educational departments and other governmental agencies.

Private kindergartens have become an important part of China's PPE system in recent years, with 56% of young children enrolled in private kindergarten in 2019.[15] The majority of private kindergartens have lower quality and are more expensive than public kindergartens. They are used by children who do not have access to public ones. The major share (85%) of the funding in private kindergartens came from tuition fees contributed by households, compared to 25% in public kindergartens.[18]

Limited empirical evidence has shown that disadvantaged households have usually paid a larger share of their income on PPE than their counterparts in China.[12,19, 20] However, little is known about how many households suffered from heavy financial burdens from paying for PPE (HBPPE). To fill in this gap in knowledge and help policy makers identify those who need support most, this study proposed a measure to identify households incurring HBPPE and conducted a case study in China. Using the 2019 China Household Finance Survey (CHFS), we assessed the national- and provincial-level prevalence of HBPPE and associated socioeconomic and other inequalities.

2. Methods

We used the CHEERS reporting guidelines in writing this paper.[21]

Ethical statement: We used secondary data that have been published and are publicly accessible.[25] Identifiers for individuals and families have been removed for public use. Users cannot identify individuals and households from this survey dataset.

This study is an observational study based on a public dataset and not a clinical study. Therefore, a patient and public involvement (PPI) statement is not applicable to this study.

2.1. Defining households with heavy financing burdens for paying for PPE (HBPPE)

We adopted the approach used by the World Bank (WB) and the World Health Organization (WHO) in defining catastrophic household health spending: a household has catastrophic health spending if it spends a certain proportion or more of its total annual expenditures (10% for the WB[22]) or annual non-food expenditures (10-40% for the WHO[23]) on health. This study defined a household incurring HBPPE if it spends more than 7% of its total annual expenditures on PPE. The selection of "7%" was based on a federal benchmark for households receiving subsidies on PPE proposed by the Office of Child Care under the U.S. Department of Health and Human Services, stating that households receive subsidies if their out-of-pocket spending on PPE exceeds 7% of household income.[24] The policy suggests that PPE spending of more than 7% of household income could be considered burdensome to households. To conduct sensitivity analysis, we allowed variation in this threshold among 7%, 10%, and 20%.

2.2. Data and samples

Confidential: For Review Only

We used nationally and provincially representative data from the 2019 China Household Finance Survey (CHFS) that collects information on household assets, income, wealth, consumption, as well as socioeconomic and demographic characteristics of sampled households and their members.[25] The CHFS employs a stratified three-stage random sample design (details in **Chapter 1 and Figure A3 of the Appendix**). The 2019 CHFS data is publicly accessible and covers 29 provinces (excluding Tibet, Xinjiang, Hong Kong, and Macau), 345 cities/districts/counties, 34,643 households, and 107,008 individuals. Our study focused on households with children, aged 3-6 years, attending kindergarten. In addition, we also included children under age of three or over age of six who attended the kindergarten (18.2%). The final sample includes 3,371 young children attending kindergartens, from 3,111 households (9% of the total sample), who reported available PPE expenditure data.

2.3. Measuring households with HBPPE

To assess if household suffered from HBPPE, we calculated estimates of household spending on PPE and total expenditures during the previous 12 months. Caregivers reported household expenditure on kindergarten over the 12 months prior to the survey. Expenditure can be categorized into two groups: (i) fees charged by schools (in-school expenditures) including tuition fees, books and materials, uniforms, meals, transport, and accommodation; (ii) fees spent on out-of-school education (out-of-school expenditure) including after-school tutoring (academically focused), extracurricular activities (e.g., learning piano or sports), and home-learning materials (e.g., books, computers). To estimate the sensitivity of the findings to the definition of PPE spending, we excluded out-of-school spending from PPE spending in an alternative analysis.

The CHFS includes a series of questions on household consumption over the 12 months prior to the survey that cover spending on food, clothing, housing, daily necessities and service, health care, transportation and communication, education and entertainment, and others (e.g. legal service). We obtained the annual household expenditure by summing spending on these items. For each household, we calculated the percentage of household total expenditure devoted to PPE. Based on the cutoff percentage value (7%, 10%, or 20%), we then constructed a dichotomous variable indicating a household's status of HBPPE: if a household spent more than 7% (10% or 20%) of its total expenditure on PPE, we classified the household as experiencing HBPPE.

2.4. Measuring socioeconomic disparities in HBPPE

We assessed disparities in the percentage of households with HBPPE by household economic status (five wealth quintiles), maternal schooling (primary school or below, middle school, high school, and college or above), residential area (rural vs. urban), and type of kindergarten (public vs. private). Disparity in this study was quantified as the absolute differences between the two sub-groups. Details on constructing these variables are presented in **Chapter 2 of the Appendix**.

2.5. Statistical Analysis

We first performed a descriptive analysis by assessing the level and socioeconomic inequalities in PPE spending per child and percentages of households with HBPPE. All estimates were

Confidential: For Review Only

adjusted for sampling weights (individual and household weights), primary sampling unit (county), and stratification variable (province). We estimated the statistical significance of inequalities in PPE spending and % of households with HBPPE between the two sub-groups (e.g. rural vs urban)[26] (details in **Chapter 3 in the Appendix**).

Considering the large variation in economic development and geographical situations across the provinces, we assessed the provincial-level disparities in the level of PPE spending per child and % of households with HBPPE across the 29 provinces, using the gap between the weighted mean of each province from the national mean (details in **Chapter 4 of the Appendix**).

To identify the factors associated with the probabilities of a household incurring HBPPE, we used a three-level logit regression model with random intercepts by province (level 3) and county (level 2), controlling for potential clustering effects at the province and county levels (Equation 1).

$$Logit\ (Y_{ijk}) = \beta_0 + \boldsymbol{\beta X_{ijk}} + u_{0k} + v_{0jk} + \varepsilon_{0ijk} \quad (1)$$

where Logit(Y_{ijk}) represents the probability of incurring HBPPE in the ith household who lives in county j and province k. **β** is a vector of coefficients for $\mathbf{X}_{ijk}$ which is a vector of variables with individual or household characteristics, including the number of children undertaking PPE, children attending private kindergarten, household size, maternal schooling, wealth quintile, and residential area. u_{0k} and v_{0jk} represent between-province random variations and between-county/within-province random variations, respectively. ε_{0ijk}represent random errors. Summary statistics for these variables are presented in **Table 1**.

Regression analyses were conducted using three different thresholds for HBPPE: 7%, 10%, and 20%. We also tested the robustness of the results including children aged 3-6 years only. To further test the sensitivity of the findings, we used proportion of household consumption on PPE as an outcome variable and conducted descriptive and regression analysis, as shown in **Chapter 5 of the Appendix**. In addition, we also used continuous household net wealth instead of net wealth quintiles as a predictor and conducted regression analysis. The STATA program (Version 16.1) was used in analyses.

3. Results

Among all the children attending kindergarten, 47.0% were girls, and 56.0% attended private kindergarten. Among the total of 3,111 households, 35.2% lived in rural areas; 11.9% or 21.0% were in the highest or lowest wealth quintile, respectively; 24.8% of the mothers held college degrees or higher, whereas over 57.2% less than a high-school diploma (**Table 1**).

3.1. Level and inequalities in household PPE spending per child

On average, PPE spending per child at the national level was CNY 8,220 (**Table 1)**, ranging from CNY4,070 in Qinghai province to CNY 23,348 in Beijing (**Figure 1 and Table 2**). Well-

Confidential: For Review Only

developed provinces had much higher PPE spending per child than the under-developed central and western provinces (**Table 2**).

For households sending children to private kindergartens, or in urban areas, or with college-educated mothers, or from the highest wealth quintile, their PPE spending per child was significantly higher than that of their counterparts (**Figure 2**). For example, PPE spending per child at the private kindergartens was approximately 30% higher higher than that at public kindergartens (CNY8,075 vs. CNY6,193). PPE spending per child in the richest households was four times more than in the poorest households (CNY17,007 vs. CNY4,182) (statistical testing in **Appendix Table A1**).

When excluding out-of-school expenditure from PPE spending, the main results remain robust (**Appendix Figure A4)**.

3.2. Level and inequalities in the percentage of HBPPE

Approximately 49.5% of households at the national level spent more than 7% of their total annual expenditure on paying for PPE, our initial threshold for HBPPE. This percentage reduced to 34.2% or 9.8% respectively if the threshold was set at 10% or 20% (**Table 1**). Provinces in the Northeastern and Middle areas of China had higher percentages of HBPPE than other areas, with Liaoning province (76.9%) the highest (**Figure 3, Table A2**). When using alternative thresholds (10% or 20%), the percentage of households with HBPPE in each province was reduced, but the pattern across the provinces remained stable (**Appendix Figures A5 and A6 and Tables A3 and A4)**.

In terms of socioeconomic inequalities (**Figure 4**), % of HBPPE was higher among those households sending children to private kindergartens (55%) than those to public kindergartens (41%). The disparity remained significant when using alternative thresholds **(Appendix Figures A7 and A8**, **Appendix Tables A5 -A7)** or when excluding out-of-school spending from PPE spending (**Appendix Figures A9-11**). % of HBPPE among the richest households was significantly lower than that among the poorest households when using threshold of 7% or 10%, after excluding out-of-school spending from PPE spending.

3.3. Factors associated with household HBPPE

Regression results (**Table 3**) identified the following factors associated with HBPPE across the three thresholds: households in the higher wealth quintiles or with smaller sizes were less likely to incur HBPPE than their counterparts; households sending children to private kindergarten or with more children in kindergarten were more likely to incur HBPPE than their counterparts.

Results from the sensitivity analyses using proportion of household spending on PPE as an outcome variable are presented in the Appendix **Figures A12-A14**, **Tables A8-A10**, continuous household net wealth as a predictor in the Appendix **Tables A11 and A12**, sample including children aged 3-6 years only in the Appendix **Tables A13.** The results are consistent with the main regression results.

Confidential: For Review Only

4. Discussion

This study proposed a measure to identify households incurring "heavy financial burdens from paying for PPE" (HBPPE) and applied the measure to data in China. Our findings showed that, in China, approximately 50% (34%, 10%) of households with young children attending kindergarten suffered from HBPPE, when a threshold of 7% (10%, 20%) of their annual expenditures on PPE was applied. Households from provinces in the Northeastern and Middle Regions had higher percentages of HBPPE on average than those from other areas. Holding everything else constant, households in the lowest-wealth quintile or those sending children to private kindergarten incurred a higher probability of HBPPE than their counterparts.

These findings are consistent with previous studies that indicate variation of PPE spending by household income levels and type of kindergartens.[12,19,20] Our study further identified significant disparities in household with HBPPE, and found that households with children attending private kindergartens had an increase of 88%-105% (across the three thresholds) in the odds of incurring HBPPE compared to households with children attending public kindergartens. The richest households could have a reduction of 37%-50% in the odds of incurring HBPPE compared to the poorest households. The finding of regional disparities in HBPPE is consistent with empirical evidence regarding the slowdown of economic development in the Northeastern and Middle Regions[27] and their lower percentage of public spending on compulsory education than other regions[28,29] and on PPE[30] in particular.

To ensure universal PPE, the Chinese government has initiated the policy of increasing the supply of public kindergartens and regulating tuition fees of private kindergartens. As shown in our study, even with the expansion of public kindergartens in recent years, more than half of young children still had to go to expensive private kingdergarten. To reduce the financial burden of PPE for households, the Chinese government needs to continue increasing the public kindergartens and make them accessible to everyone. Meanwhile, providing PPE subsidies to low-income households, especially those with children going to private kindergartens, is necessary for ensuring that poor children have access to affordable PPE.

This study has several limitations. First, the study only included children enrolled in kindergarten at the time of the survey. Children who were not able to attend kindergarten were not included in the analysis sample and they were more likely to live in poor or remote rural areas with limited or even no access to kindergarten. Excluding those children could lead to an underestimation of the probability of HBPPE among the poorest or rural households. Second, the study only used cross-sectional data. With a new wave of survey data available in the future, we will be able to identify the impact of related governmental policies on HBPPE over time. Third, the study used three threshold cut-offs to measure households with HBPEE. However, there is no information from households on what percentage of their total spending on PPE would be considered affordable. Fourth, applying a threshold of HBPPE to households with different income levels may underestimate the burden of PPE borne by low-income households because they have smaller proportions of discretionary income after paying for basic necessities compared to those in the middle or upper quintiles.

https://mc.manuscriptcentral.com/bmjpo

Confidential: For Review Only

Despite these limitations, this study proposed a measure that can be used by researchers to identify households with financial barriers to access PPE in LMICs. Using this measure in China, the study provides solid evidence to inform policy makers on which households suffered HBPPE and what could be potentially effective policy instruments to promote equal access to PPE. Future PPE policy in China should consider establishing a financial-aid system on PPE for children who need it most and expanding the enrollment of children from disadvantaged households in the public kindergarten system.

Source of funding

This work was supported by Ministry of Educaiton in China (7122900028), UNICEF China Office (8430500872), and the UKRI GCRF Harnessing the Power of Global Data to Advance Young Children's Learning and Development (ES/T003936/1).

Conflict of interest

The authors have declared no competing interests.

Authors' contribution

YW was responsible for survey coordination, conducted data analysis, and wrote the first draft of the manuscript. KL conducted data analysis on inequalities and regressions and took responsibility for the integrity of data analysis. LK participated data analysis and drew some figures. YS was the PI for expenditures of PPE, survey design, initiated the study, supervised YW's work. CL conceptualized the study, designed the analysis plan, and mentored data analysis. All authors have made contributions to data interpretation and manuscript writing. All authors approve the manuscript for submission

Data sharign statement

Data from the China Household Finance Survey (CHFS) is available at the website of the Survey and Research Center for China Household Finance, at https://chfs.swufe.edu.cn/

The analysis code is available from the corresponding author upon request.

References

1. Black, M. M., Walker, S. P., Fernald, L., Andersen, C. T., DiGirolamo, A. M., Lu, C., McCoy, D. C., Fink, G., Shawar, Y. R., Shiffman, J., Devercelli, A. E., Wodon, Q. T., Vargas-Barón, E., Grantham-McGregor, S., & Lancet Early Childhood Development Series Steering Committee. (2017). Early childhood development coming of age: science through the life course. *Lancet*, 389(10064), 77–90.
2. Chetty, R., Friedman, J. N., Hilger, N., Saez, E., Schanzenbach, D. W., & Yagan, D. (2011). How does your kindergarten classroom affect your earnings? Evidence from

Confidential: For Review Only

Project Star. *Quarterly Journal of Economics*, 126(4), 1593–1660.
3. Heckman, J., Pinto, R. & Savelyev, P. (2013). Understanding the mechanisms through which an influential early childhood program boosted adult outcomes. *American Economic Review*, 103 (6), 2052-86.
4. Holla, Alaka, Magdalena Bendini, Lelys DinarteIva, Iva Trako. (2021). Is Investment in Preprimary Education Too Low? Lessons from (Quasi) Experimental Evidence across Countries. Washington, DC: World Bank Policy Research Working Paper; No. 9723.
5. Nores, M., & Barnett, W. S. (2010). Benefits of early childhood interventions across the world: (Under) Investing in the very young. *Economics of Education Review, 29(2), 271–282.*
6. Berlinski, S., & Schady, N. (2015). The Early Years: Child Well-being and the Role of Public Policy. MacMillan Press, New York.
7. Engle, P. L., Fernald, L. C., Alderman, H., Behrman, J., O'Gara, C., Yousafzai, A., Cabral de Mello, M., Hidrobo, M., Ulkuer, N., Ertem, I., Iltus, S., & the Global Child Development Steering Group. (2011). Strategies for reducing inequalities and improving developmental outcomes for young children in low-income and middle-income countries. *Lancet,* 378(9799), 1339–1353.
8. OECD. (2006). Starting Strong II: Early Childhood Education and Care. OECD Publishing, Paris.
9. Sustainable Development Goals, 2030. Retrieved from: https://www.undp.org/sustainable-development-goals

10. UNESCO. Institute for Statistics. http://uis.unesco.org/en/files/who-pays-what-education-national-revealed-through-accounts-2016-en-pdf

11. Gil, J.D., Ewerling, F., Ferreira, L. Z., & Barros, A.J. (2020). Early childhood suspected developmental delay in 63 low- and middle-income countries: Large within- and be-tween-country inequalities documented using national health surveys. *Journal of global health*,10(1), 010427.

12. Su, Y. & Rao, N. (2020). Relationship between family's socioeconomic status and parental investments in early learning: Evidence from the China Family Panel Studies. *Peking University Education Review*, 18(3), 86-103. In Chinese
13. Lu, C., Cuartas J, Fink G, et al. (2020). Inequalities in early childhood care and development in low/middle-income countries: 2010–2018. *BMJ Global Health* 2020;5:e002314.
14. Ministry of Education of the People's Republic of China. http://www.moe.gov.cn/publicfiles/business/htmlfiles/moe/moe_2792/index.html

15. China Educational Finance Statistical Yearbook (2020).
16. Song, Y. (2019). Principal contradictions and reformation of public finance in early childhood education and care in China. *China Economics of Education Review*, 4(3), 19-48. In Chinese
17. Yuan, L., & Zhao, J. (2019). Issues in the fiscal policy for preschool education. *China Economics of Education Review*, 2019,4(03), 49-63. In Chinese

Confidential: For Review Only

18. Ministry of Education of the People's Republic of China. (2020). Educational statistics in 2019. Retrieved from: http://www.moe.gov.cn/jyb_sjzl/moe_560/jytjsj_2019/qg/
19. Tian, Z., Zhang, X., & Yuan, L. (2011). The disparities of preschool educational resources for children with different household registration: A survey in Beijing. *Renmin University of China Education Journal*, (03), 165-180. In Chinese
20. Liu, Y., & Song, Y. (2013). Survey on the family expenditure for pre-school education of 3-to-6-year-old children in urban families. *Journal of Central China Normal University (Humanities and Social Sciences)*, 52(01), 155-160. In Chinese
21. Husereau D, Drummond M, Petrou S, Carswell C, Moher D, Greenberg D, Augustovski F, Briggs AH, Mauskopf J, Loder E. Consolidated Health Economic Evaluation Reporting Standards (CHEERS) statement.
22. Wagstaff, A., Glores G, Hsu J. et al. Progress on catastrophic health spending in 133 countries: a retrospective observational study. *Lancet Global Health* 2018;6:e169-79.
23. Xu, K., Evans, D. B., Kawabata, K., Zeramdini, R., Klavus, J., & Murray, C. J. (2003). Household catastrophic health expenditure: a multicountry analysis. *Lancet*, 362(9378), 111–117.
24. Office of Child Care (OCC), Administration for Children and Families (ACF), Department of Health and Human Services (HHS). (2016). Child Care and Development Fund (CCDF): Final rule. Retrieved from https://www.federalregister.gov/documents/2016/09/30/2016-22986/child-careand-development-fund-ccdf-program
25. Survey and Research Center for China Household Finance. https://chfs.swufe.edu.cn/
26. Cumming, G., & Finch, S. (2005). Inference by eye: Confidence intervals and how to read pictures of data. *American Psychologist*, 60(2),170-180.
27. Wang, J., Yuan, C.,& Ma, N. (2022). Government competition, factor flow and regional collapse. *Journal of Xi'an Jiaotong University (Social Science)*, 42(2), 40-52. In Chinese
28. Shang, W., Lu, S., & Li, T. (2020). The collapse of central regions in the development of compulsory education in China: Representation, influencing factors, and policy rationales. *Peking University Education Review*, 18(2), 172-192. In Chinese
29. Lei, W., Qian, J.,& Ma, H. (2014). Studies on compulsory education investment collapse in central China. *Economics and Education*, 2014(6), 1-9. In Chinese
30. Li, Z., Gong, X., & Qian, J. (2018). Studies on preschool investment sinking in central China. *China Economics of Education Review*, 3(4), 78-90. In Chinese

Confidential: For Review Only

Table 1. Summary statistics of variables used in the analysis

Variable	Observation	Mean	SD	Min	Max
Child-level variables					
PPE expenditure per child	3,371	8,220	10,088	0.000	204,000
Female	3,371	0.470	0.499	0.000	1.000
Private kindergarten	3,371	0.560	0.496	0.000	1.000
Household-level variables					
PPE expenditure per household (CNY)	3,111	8,907	10,824	0.000	204,000
% of household expenditure on PPE	3,111	0.094	0.096	0.000	0.887
HBPPE (7%)	3,111	0.495	0.500	0.000	1.000
HBPPE (10%)	3,111	0.342	0.475	0.000	1.000
HBPPE (20%)	3,111	0.098	0.297	0.000	1.000
Number of children undertaking PPE	3,111	1.084	0.294	1.000	4.000
Children in private kindergarten	3,091	0.557	0.497	0.000	1.000
Household size	3,111	5.131	1.576	2.000	15.000
Per capita household net wealth (CNY)	3,111	241,004	562,588	-1,974,739	17,020,206
Household net wealth quintile	3,111				
Quintile 1	3,111	0.210	0.407	0.000	1.000
Quintile 2	3,111	0.252	0.434	0.000	1.000
Quintile 3	3,111	0.232	0.422	0.000	1.000
Quintile 4	3,111	0.187	0.390	0.000	1.000
Quintile 5	3,111	0.119	0.324	0.000	1.000
Maternal schooling					
Primary school and below	3,111	0.136	0.343	0.000	1.000
Middle school	3,111	0.436	0.496	0.000	1.000
High school	3,111	0.180	0.384	0.000	1.000
College or above	3,111	0.248	0.432	0.000	1.000
Rural residential area	3,111	0.352	0.478	0.000	1.000

Notes: The number of children undertaking PPE indicates the number of children who attended kindergarten in a household. Household size is defined as the total number of household members in a household. A household unit is defined as all members of a housing unit related by blood, marriage, or some other legal arrangement; or two or more persons use incomes to make joint expenditures; or a single person living with others but is financially

Confidential: For Review Only

independent. Thus a household may include grandparents, especially in rural area. When analyzing households that have children in private kindergarten, we excluded the observations of 20 households that had children attending both public and private kindergarten. CNY: Chinese Yuan.

Table 2. Average level of PPE spending per child across 29 provinces and weighted mean difference of the mean between the provincial level and the national level

Province	PPE spending at the provincial level	Weighted mean difference*
Beijing	23,348	246
Tianjin	19,487	135
Liaoning	13,417	189
Zhejiang	11,057	155
Shanghai	10,279	51
Neimenggu	10,095	50
Guangdong	9,309	162
Jiangsu	8,163	47
Hunan	8,030	34
Henan	7,942	41
Fujian	7,877	15
Shaanxi	7,740	11
Chongqing	7,062	-6
Heilongjiang	6,980	-10
Hubei	6,964	-16
Shandong	6,852	-35
Jilin	6,850	-10
Hainan	6,614	-5
Jiangxi	6,202	-38
Shanxi	6,090	-33
Yunnan	6,004	-47
Ningxia	5,496	-9
Sichuan	5,456	-113
Anhui	4,869	-113
Guizhou	4,674	-69
Guangxi	4,536	-100
Gansu	4,506	-54
Hebei	4,246	-168
Qinghai	4,070	-14

*: The measures took zero as the reference, with positive values indicating provincial means higher than the national

Confidential: For Review Only

average, and negative values indicating provincial means lower than the national average.

Table 3. Multilevel logit regression results on factors associated with household status on HBPPE defined with various thresholds

	HBPPE_7% (Odds ratio)	HBPPE_10% (Odds ratio)	HBPPE_20% (Odds ratio)
Household net wealth quintile (ref. Quintile 1)			
Quintile 2	0.833*	0.756**	0.665**
Quintile 3	0.560***	0.584***	0.581***
Quintile 4	0.598***	0.697***	0.595**
Quintile 5	0.634***	0.512***	0.503***
Mother's education (ref. Primary school and below)			
Middle school	1.192	1.200	1.095
High school	1.004	1.127	1.146
College/above	1.208	1.391*	1.428
Number of children undertaking PPE	3.451***	3.375***	3.233***
Household having children in private kindergarten	1.973***	2.050***	1.880***
Household size	0.799***	0.766***	0.789***
Rural	0.820**	0.892	1.225
Constant	0.744	0.407***	0.061***
N	3091	3091	3091
ICC (Province)	0.018	0.029	0.048
ICC (County | Province)	0.110	0.108	0.187

Notes: * p<0.10, ** p<0.05, *** p<0.01. ICC refers to intraclass correlations; ICC (Province) denotes the intraclass correlations at the province level, and ICC (County | Province) denotes the intraclass correlations at the county and province level. When we define the HBPPE measure with the threshold of 7%, for example, we estimated that province random effects composed 1.8% of the total residual variance of the HBPPE measure, while county and province random effects composed 11.0% of the total residual variance of the HBPPE measure.

Confidential: For Review Only

Figure 1. Average level of PPE spending per child across 29 provinces

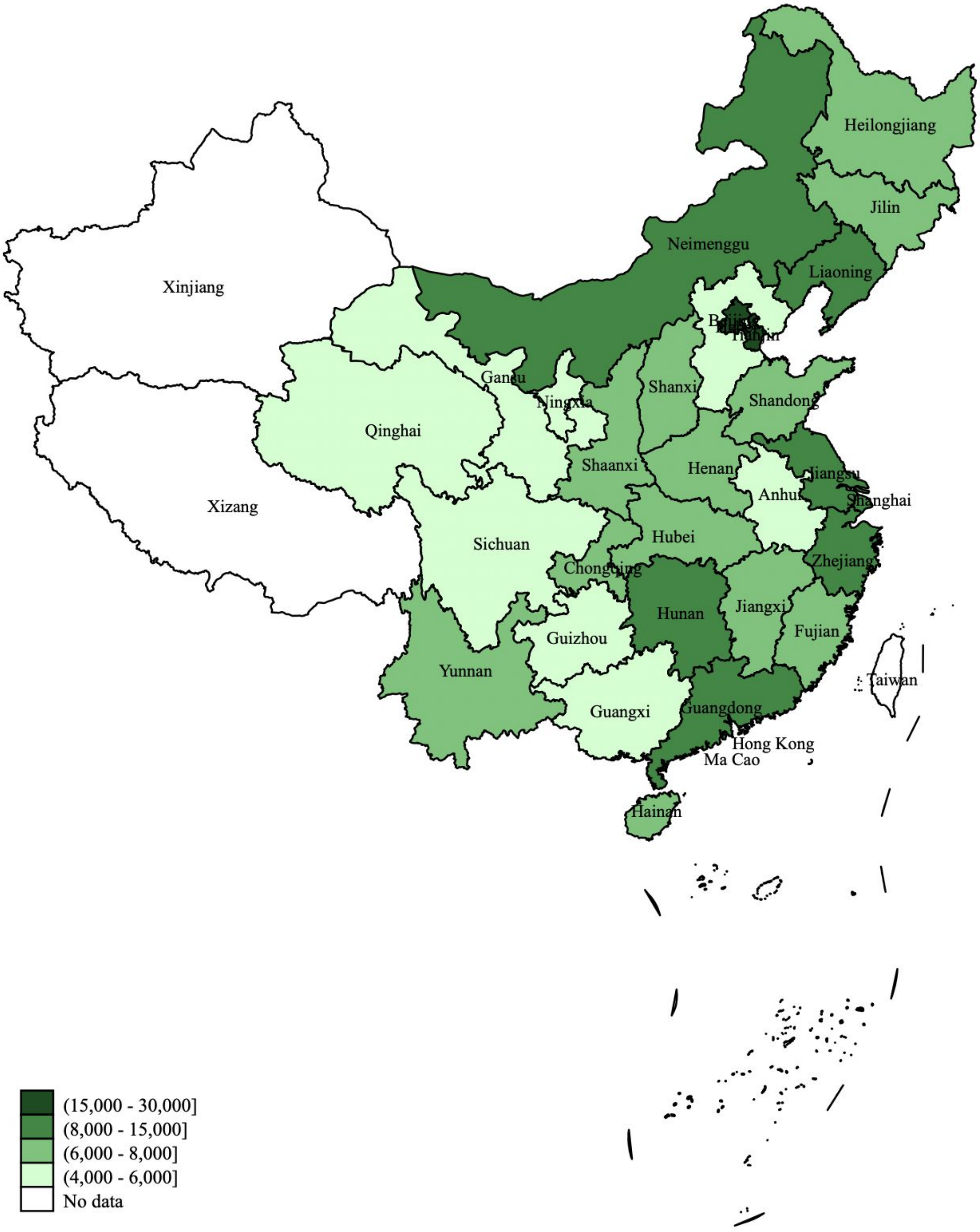

Figure 2. Level and inequalities of PPE spending per child by gender, type of kindergarten, maternal education, household net wealth quintile, and current residential area

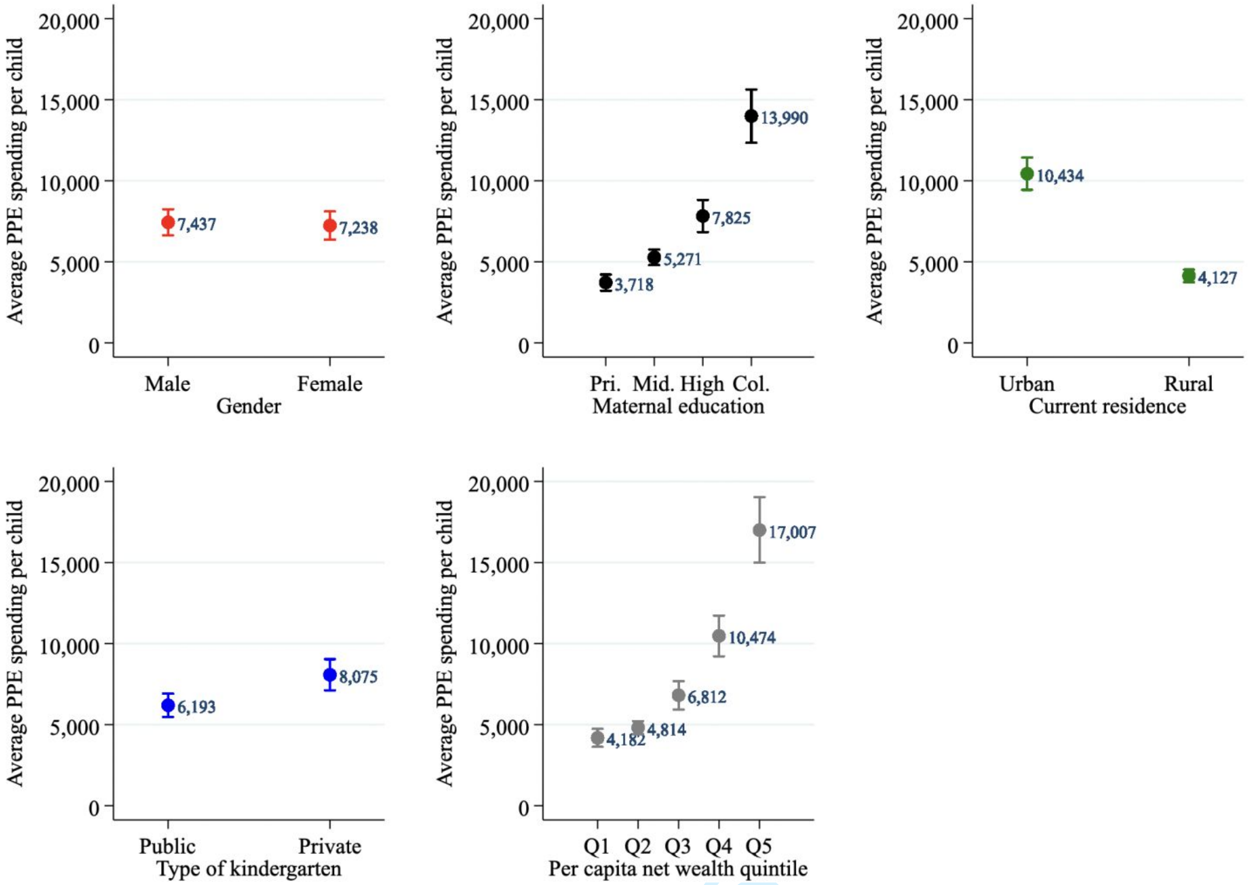

Notes: “Pri.”, “Mid.”, “High”, and “Col.” refers to maternal schooling at the primary and below, middle, high, and college and above levels, respectively.

For peer review Only

Figure 3. Level of % of households with HBPPE (threshold=7%) across 29 provinces

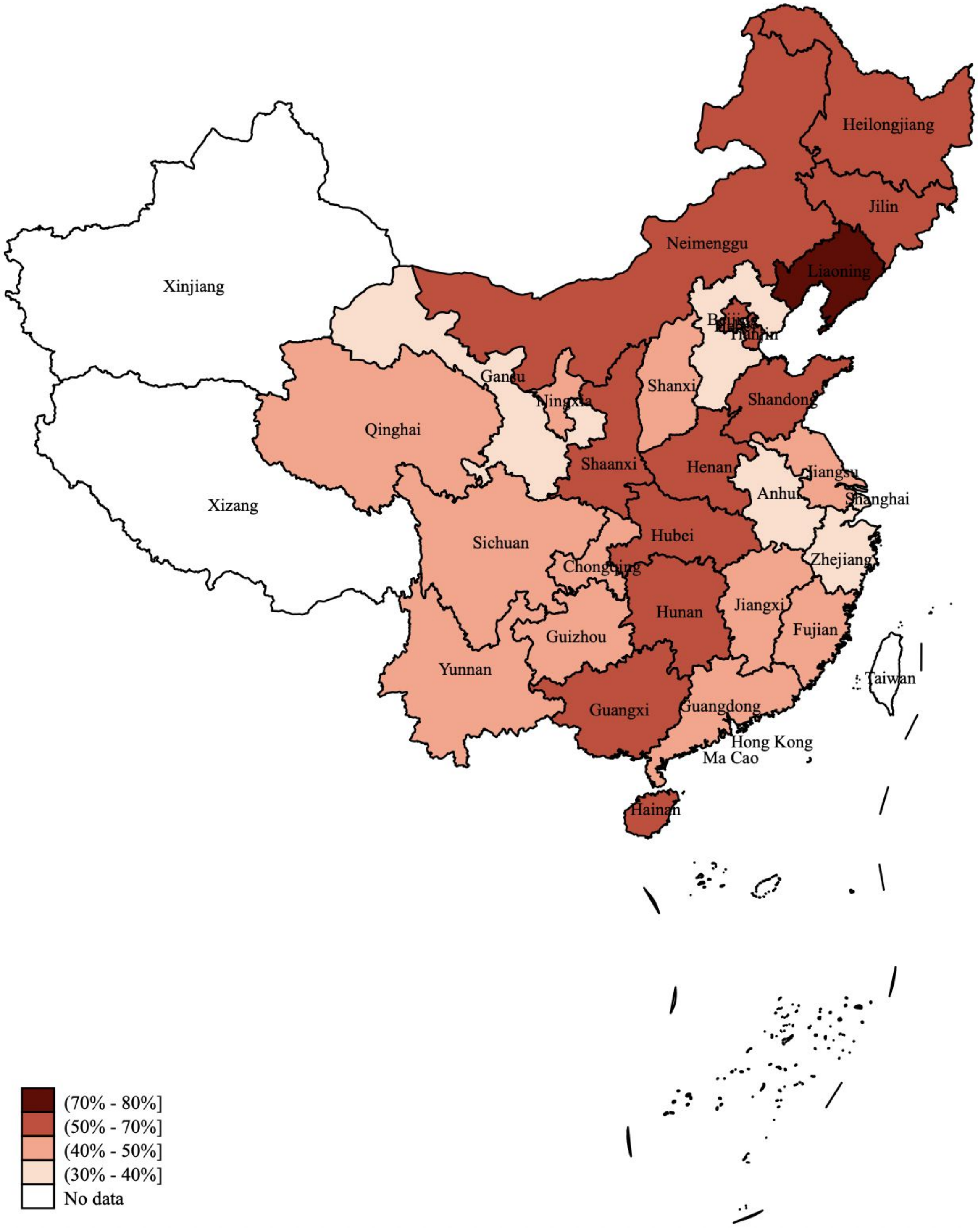

Figure 4. Level and inequalities of % of households with HBPPE (thresehold=7%) by type of kindergarten, maternal education, household net wealth quintile, and current residential area

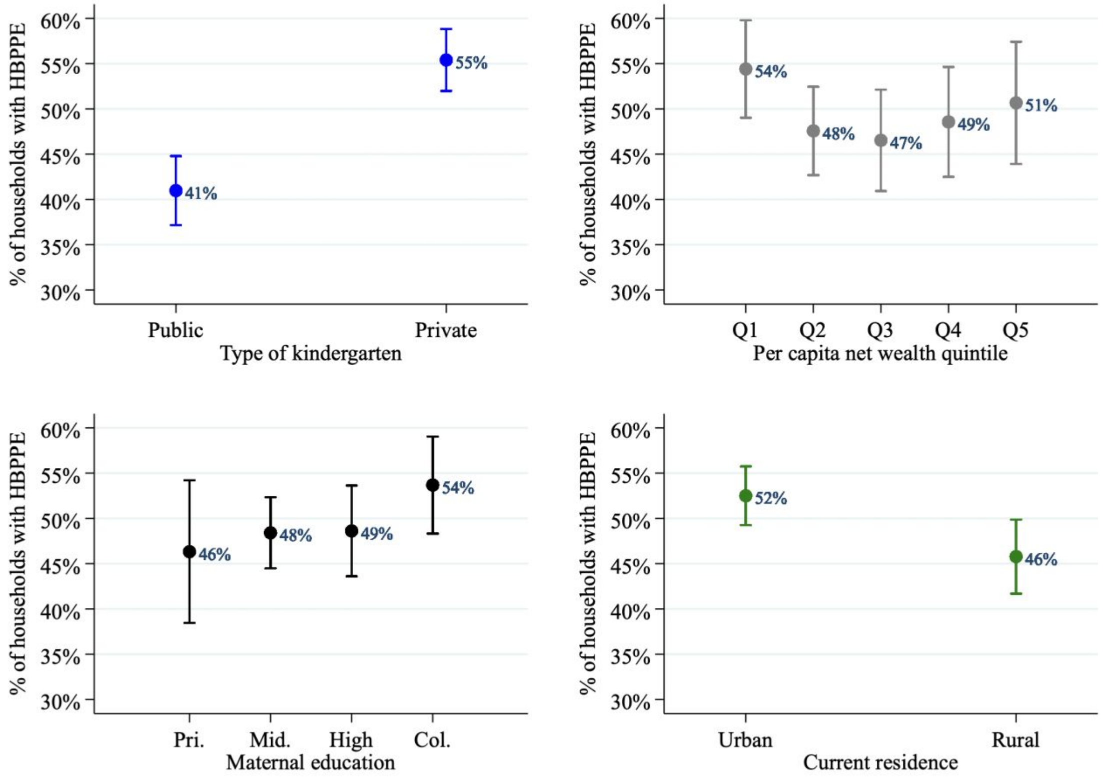

Notes: "Pri.", "Mid.", "High", and "Col." refers to maternal schooling at the primary and below, middle, high, and college and above levels, respectively.

Appendix

Chapter 1 Sampling Process of the China Household Finance Survey (CHFS)
Chapter 2 Constructing variables on socioecnomic charisteristics
Chapter 3 Testing the statistical significance of inequalities in PPE spending and HBPPE using Cumming and Finch's "rule of eye"
Chapter 4. Calculating provincial-level disparities in PPE spending and % of households with HBPPE
Chapter 5. Sensitivity tests using proportion of households spending on PPE as outcome variable and using continuous household net wealth as a predictor
Figure A1. Government education spending on preschool education
Figure A2. Preschool enrollment and gross enrollment rate
Figure A3. Geographical distribution of the sampled counties
Figure A4. Level and inequalities of PPE spending per child excluding out-school expenditure
Figure A5. Level of % of households with HBPPE (threshold=10%) across 29 provinces
Figure A6. Level of % of households with HBPPE (threshold=20%) across 29 provinces
Figure A7. Level and inequalities of % of households with HBPPE (thresehold=10%) by type of kindergarten, maternal education, household net wealth quintile, and current residential area
Figure A8. Level and inequalities of % of households with HBPPE (thresehold=20%) by type of kindergarten, maternal education, household net wealth quintile, and current residential area
Figure A9. Level and inequalities of % of households with HBPPE (threshold=7%) by excluding out-school spending from PPE spending
Figure A10. Level and inequalities of % of households with HBPPE (threshold=10%) by excluding out-school spending from PPE spending
Figure A11. Level and inequalities of % of households with HBPPE (threshold=20%) by excluding out-school spending from PPE spending
Figure A12. Level of % of household expenditure on PPE across 29 provinces
Figure A13. Level and inequalities of % of household expenditure on PPE by type of kindergarten, maternal education, household net wealth quintile, and current residential area
Figure A14. Level and inequalities of % of household expenditure on PPE by excluding out-of-school spending from PPE spending

Table A1. Significance testing of inequalities in PPE spending per child using Cumming and Finch's "rule of eye"
Table A2. Level of % of households with HBPPE (threshold=7%) across 29 provinces and weighted mean difference of the mean between the provincial level and the national level
Table A3. Level of % of households with HBPPE (threshold=10%) across 29 provinces and weighted mean difference of the mean between the provincial level and the national level
Table A4. Level of % of households with HBPPE (threshold=20%) across 29 provinces and weighted mean difference of the mean between the provincial level and the national level
Table A5. Significance testing of inequalities in HBPPE (threshold=7%) using Cumming and Finch's "rule of eye"

Confidential: For Review Only

Table A6. Significance testing of inequalities in HBPPE (threshold=10%) using Cumming and Finch's "rule of eye"

Table A7. Significance testing of inequalities in HBPPE (threshold=20%) using Cumming and Finch's "rule of eye"

Table A8. Level of % of household expenditures on PPE across 29 provinces and weighted mean difference of the mean between the provincial level and the national level

Table A9. Multilevel linear regression results on factors associated with logged % of household expenditure on PPE

Table A10. Beta regression results on factors associated with % of household expenditure on PPE

Table A11. Multilevel logit regression results on factors associated with household status on HBPPE by setting household net wealth as a continuous variable

Table A12. Multilevel linear regression and Beta regression results on factors associated with % of household expenditure on PPE by setting household net wealth as a continuous variable

Table A13. Multilevel logit regression results on factors associated with household status on HBPPE defined with various thresholds, children aged 3-6 years only

Chapter 1 Sampling Process of the China Household Finance Survey (CHFS)

The CHFS survey employs a stratified three-stage probability proportion-to-size random sample design. The primary sampling units include 2,585 counties/provincial cities in China. Tibet, Xinjiang, Hong Kong, Macao, and Chinese Taipei are not included in the primary sampling stage. The second stage of sampling involves selecting residential committees/villages from the counties/cities. The last stage involves selecting households from the residential committees/villages. A household unit is defined as all members of a housing unit related by blood, marriage, or some other legal arrangement; or two or more persons use incomes to make joint expenditures, or a single person living with others but is financially independent.

The CHFS is nationally and provincially representative survey conducted by the Southwestern University of Finance and Economics in China.[1] The CHFS collects rich information on household assets, income, wealth, and consumption. It also collects socioeconomic and demographic information on the sampled households and their members, including residential areas, household members' ages, gender, education levels, marital status, etc. The CHFS data are publicly available and have been widely used in the fields of economics, sociology, and education. By the end of November 2021, more than 35,000 researchers and 3,000 research institutions had registered and applied for the dataset, and the related studies have been published in top peer-reviewed journals.[2] Since 2017, the CHFS survey collaborated with the China Institute for Educational Finance Research and included a module on household spending on young children's care and education. The module includes questions on young children's schooling status, types of schooling, and household spending on various items that are related to preprimary attendance.

The CHFS 2019 covers 29 provinces (excluding Tibet, Xinjiang, Inner Mongolia, Hong Kong, and Macau), 345 cities/districts/counties, 34,643 households, and 107,008 individuals. Our focus was on household spending related to attending kindergarten. In China, kindergarten is usually

Confidential: For Review Only

offered to children 3-6 years old, but children under age 3 and over age 6 may also attend. In this study, we focused on children attending kindergarten, including those under age 3 or over age 6 who still attended kindergarten (18.2%). The 2019 data includes 4,085 young children aged 3-6 years old and 2,797 of these children attended kindergartens, yielding a 68.5% net enrollment rate.[3] The number of children attending kindergarten in all age groups is 3,415, yielding 83.6% of the gross enrollment rate, very close to the governmental estimate (83.4%).[4] Among households in this dataset, 42 (0.18%) didn't report spending information on PPE, and two reported PPE spending exceeding total household expenditures, both of which groups were excluded. The final sample includes 3,371 young children attending kindergartens, from 3,111 households (9% of the total sample), who reported available PPE expenditure data.

References

1. Gan, L., Yin, Z., Jia, N., Xu, S., & Ma, S. (2013). Data you need to know about China, Springer.
2. Survey and Research Center for China Household Finance, at https://chfs.swufe.edu.cn/
3. The net enrolment rate (NER) is the number of students of the age of preprimary education (age 3-6) that are enrolled in that level of education, expressed as a percentage of the total population in that age group.
4. The gross enrollment rate (GER) is the total enrollment in preprimary education, regardless of age, expressed as a percentage of the population in the official age group (age 3-6) corresponding to this level of education. The GER can exceed 100% because of early or late entry and/or grade repetition.

Chapter 2 Constructing variables on socioecnomic charisteristics

Household economic status. In this study, we used per capita household net wealth to measure household economic status. Household net wealth was derived by calculating the difference between a household's assets and its debts. Household total assets include financial assets (e.g., cash, deposit balances, social security, stock/bond value, etc.) and non-financial assets (e.g., agricultural capital, industrial capital, commercial capital, housing, vehicles, and durable goods). Total household debts include financial asset debts, agricultural debts, business debts, housing debts, vehicle debts, other non-financial asset debts, educational debts, credit card debts, medical-care debts, and other debts. In this study, we divided households into five quintiles according to their per capita net wealth. We examined the difference in % of households with HBPPE across the five wealth quintiles.

Maternal schooling. We divided maternal schooling into four categories: primary school or below, middle school, high school, and college or above. About 12% of households in the sample of the study did not report the mothers' education; we replaced the missing data with the predicted values of mother's education by regressing mothers' education on fathers' education (ordered logit regression coefficient = 1.217, $p < 0.001$; correlation coefficient = 0.746) or household heads' education (ordered logit regression coefficient = 1.393, $p < 0.001$; correlation coefficient = 0.632).

Residential area. Because the urban-rural divide is still one of the main structural problems

Confidential: For Review Only

facing the schooling system in China, we also examined the disparities between urban and rural residential areas.

Type of kindergarten. Kindergartens in China can be categorized into public and private types, as discussed above. Households need to pay higher tuition fees to attend private kindergartens than public kindergartens. We assessed the disparities in the percentages of households with HBPPE by kindergarten type.

Chapter 3 Testing the statistical significance of inequalities in PPE spending and HBPPE using Cumming and Finch's "rule of eye"

To test whether inequalities in PPE spending or HBPPE significantly existed between the two sub-groups (e.g. male vs female), we compared the 95% confidence intervals (CIs) of the two estimates using Cumming and Finch's "rule of eye" for comparing two independent means.[1] We calculated three components that were required to implement the "rule of thumb": marginal errors, overlap of the 95% CIs of the two estimates, and proportion overlap. (1) Marginal error refers to the absolute difference between the mean and its lower or higher bound of a 95% CI. (2) An overlap of CIs refers to the difference between one estimate's higher bound and the other estimate's lower bound. (3) Proportion overlap is then calculated as the overlap of the 95% CIs between the two estimates divided by the average marginal errors between the two estimates.

Using Cumming and Finch's "rule of eye", we obtained proportion overlap using Equation PA1 or PA2 as follows, depending on the size of mean estimates for the two sub-groups. For example, when comparing the mean estimates for gender, if the mean estimate for male is lower than that for female, then

$$Proportion\ overlap = \frac{95\%\ CI\ upper\ bound_{male} - 95\%\ CI\ lower\ bound_{female}}{(Margin\ of\ error_{male} + Margin\ of\ error_{female})/2} \qquad \textbf{(PA1)}$$

Or, if the mean estimate for male is higher than that for female, then

$$Proportion\ overlap = \frac{95\%\ CI\ upper\ bound_{female} - 95\%\ CI\ lower\ bound_{male}}{(Margin\ of\ error_{male} + Margin\ of\ error_{female})/2} \qquad \textbf{(PA2)}$$

According to the "rule of eye", when sample sizes of two samples are both at least 10, and the margins of error between the two estimates do not differ by more than a factor of two, a proportion overlap less than 0.5 indicates a significant statistical relationship at the 0.05 level (i.e. p<0.05).

References

1. Cumming, G., & Finch, S. (2005). Inference by eye: Confidence intervals and how to read pictures of data. *American Psychologist*, 60(2),170-180.

Chapter 4. Calculating provincial-level disparities in PPE spending and % of households wth HBPPE

Considering the large variation in economic development and geographical situations across the provinces, we further assessed the provincial-level disparities in the level of PPE spending per

Confidential: For Review Only

child and % of households with HBPPE across the 29 provinces. The information will help the central government to identify the provinces with high percentages of households with HBPPE. Provincial-level inequalities were estimated with the weighted mean from the national mean, which was calculated as the values of the mean difference between each province and the national average, weighted by each province's population share (= $\frac{pop_i \times (r_i - r)}{pop}$), where *pop* is the national population size, pop_i is the population size of province *i*, *r* is the national mean, r_i is the mean of province *i*.). The measures took zero as the reference, with positive values indicating provincial means higher than the national average, and negative values indicating provincial means lower than the national average.

Chapter 5. Sensitivity tests using proportion of households spending on PPE as the outcome variable and using continuous household net wealth as a predictor

We first calculated the provincial-level disparities in the % of household expenditure on PPE across the 29 provinces. On average, households in the Northeastern and middle areas spend higher % of household expenditure on PPE, with Shaanxi (15.4%) and Liaoning (14.8%) showing the highest levels. Hebei (6.4%) and Gansu (6.0%) had the lowest levels (**Appendix Figure A12 and Table A8)**. The pattern across the provinces is similar to the % of households with HBPPE.

In terms of socioeconomic inequalities (**Figure A13**), the % of expenditure on PPE was higher among those households sending children to private kindergartens (10.5%) than to public kindergartens (8.1%). Statistically significant differences were not observed across urban and rural households, households with different levels of maternal education, or households in different wealth quintiles. The results are consistent with the ones in the text using dichotomous HBPPE.

Figure A14 shows results excluding out-of-school spending from the PPE measure. The difference between private and public kindergartens increases. Also the difference between the highest wealth quintile and the lowest one is statistically significant.

Tables A9 and A10 present the regression results using % of household total expenditure on PPE as the outcome variable. We used both a multilevel linear regression and a beta regression for estimation. The results are consistent with the main regression results in the text.

Figure A12. Level of % of household expenditure on PPE across 29 provinces

Tables A11 and A12 present the regression results using continuous household instead of net wealth quintiles as the predictor. We used a multilevel logit regression, multilevel linear regression and a beta regression for estimation. The results are consistent with the main regression results.

Confidential: For Review Only

Figure A1. Government education spending on preschool education (Source: China Educational Finance Statistical Yearbook)

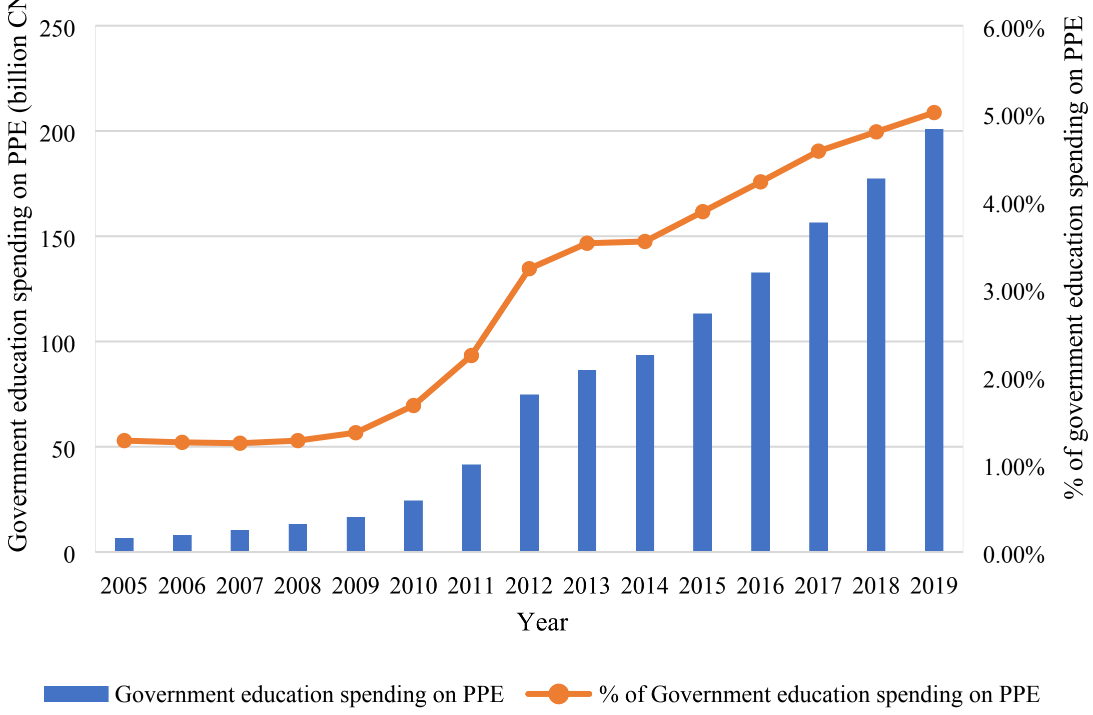

Figure A2. Preschool enrollment and gross enrollment rate (Source: China Education Statistical Yearbook)

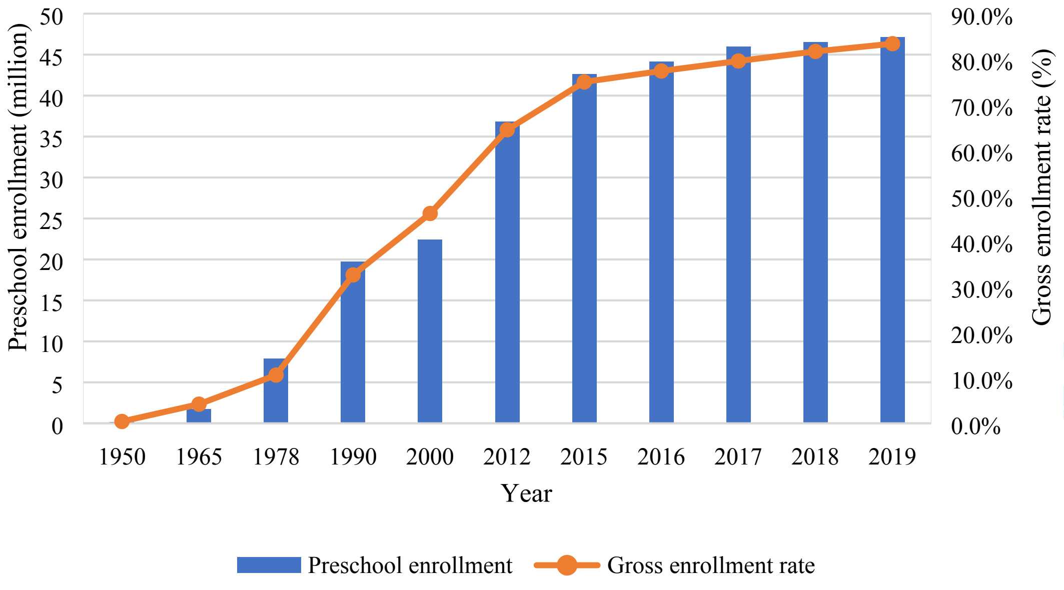

Figure A3. Geographical distribution of the sampled counties

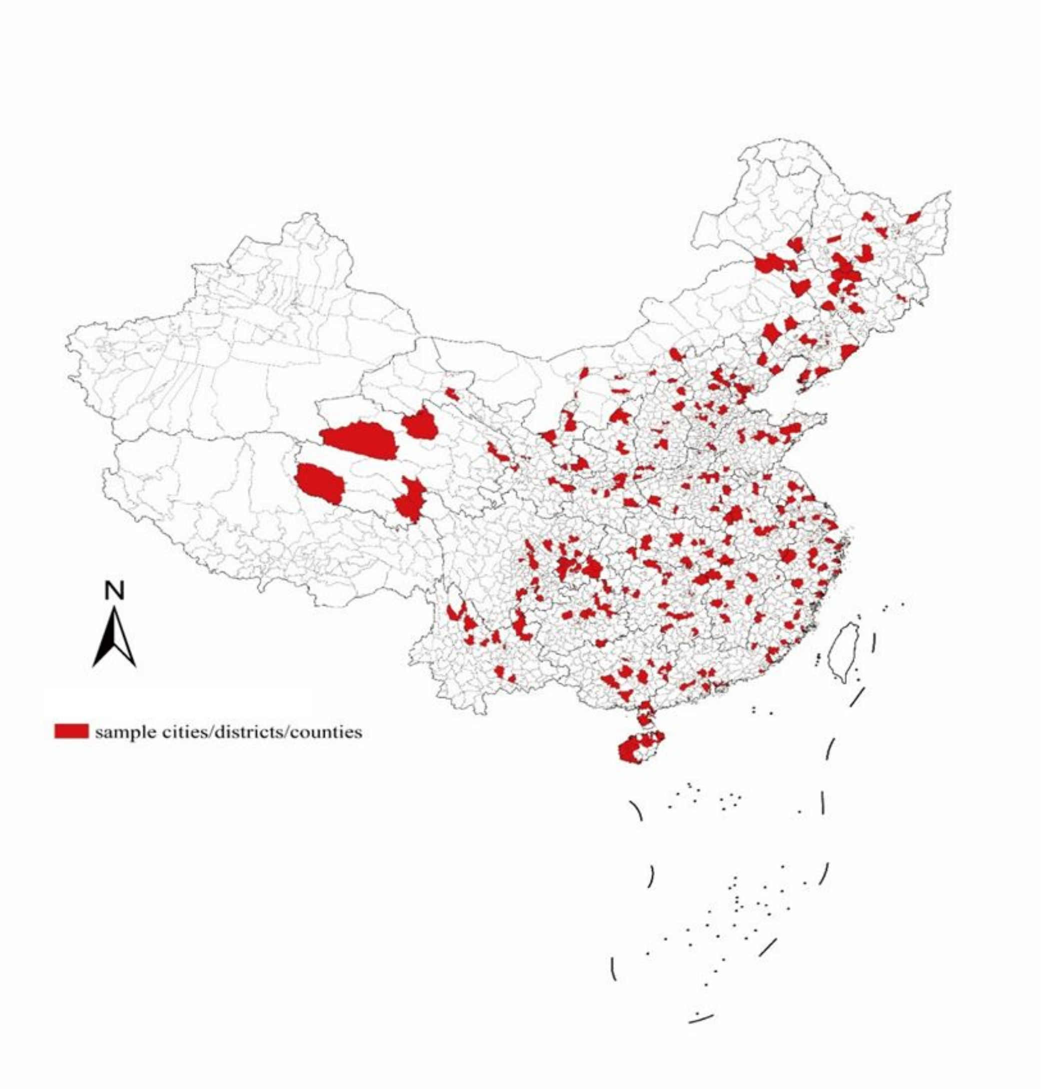

w Only

Figure A4. Level and inequalities of PPE spending per child excluding out-school expenditure

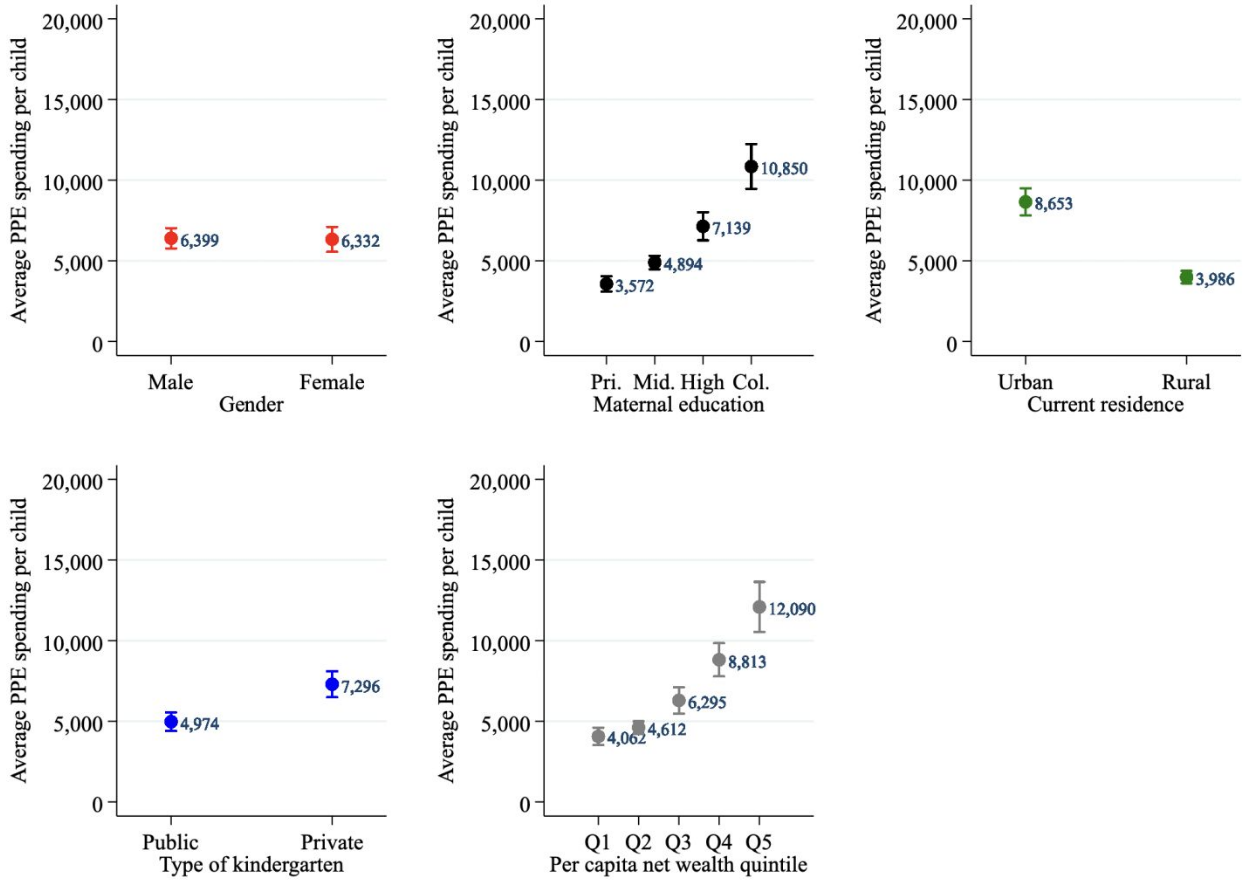

Notes: “Pri.”, “Mid.”, “High”, and “Col.” refers to maternal schooling at the primary and below, middle, high, and college and above levels, respectively.

For Peer Review Only

Figure A5. Level of % of households with HBPPE (threshold=10%) across 29 provinces

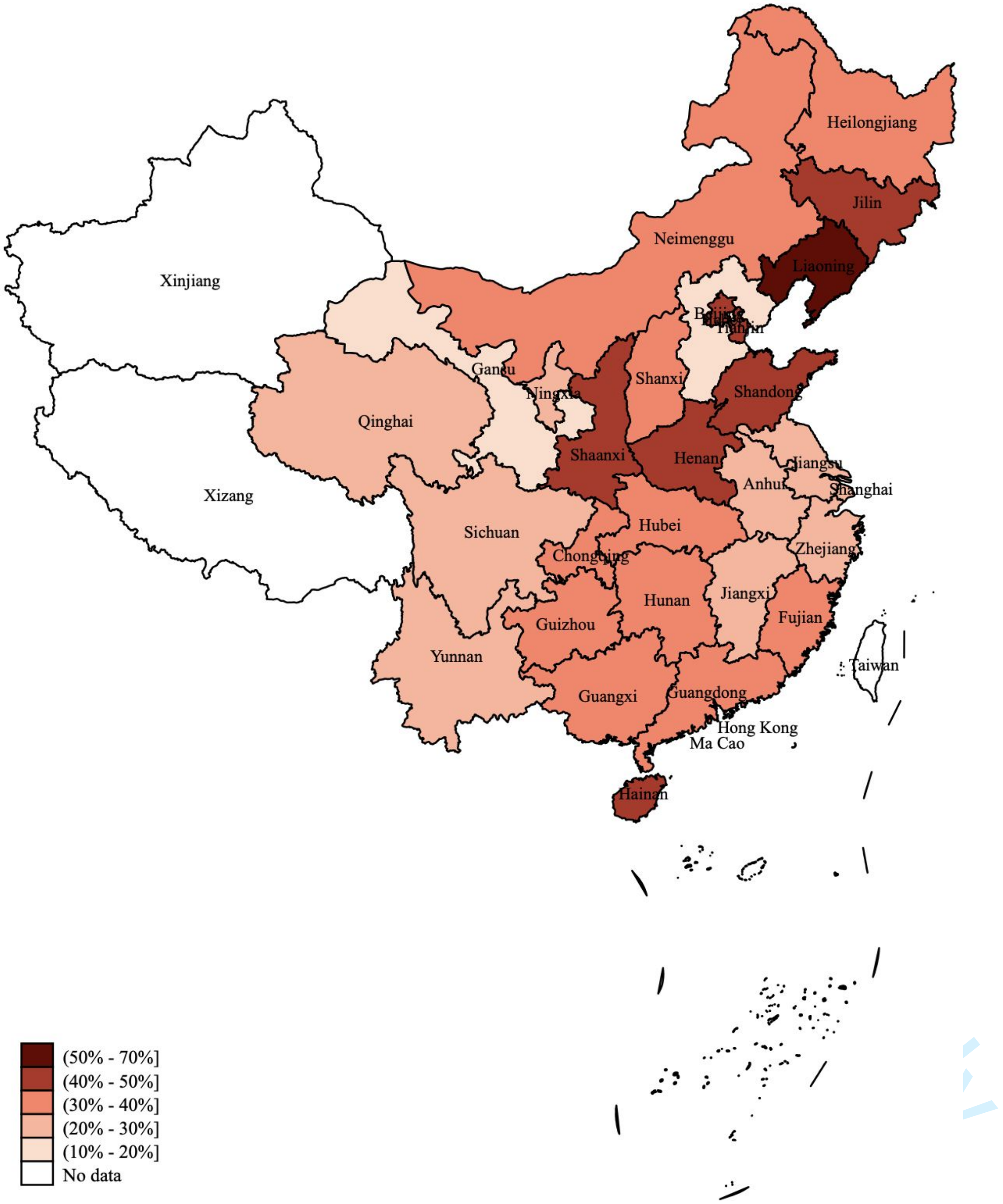

Figure A6. Level of % of households with HBPPE (threshold=20%) across 29 provinces

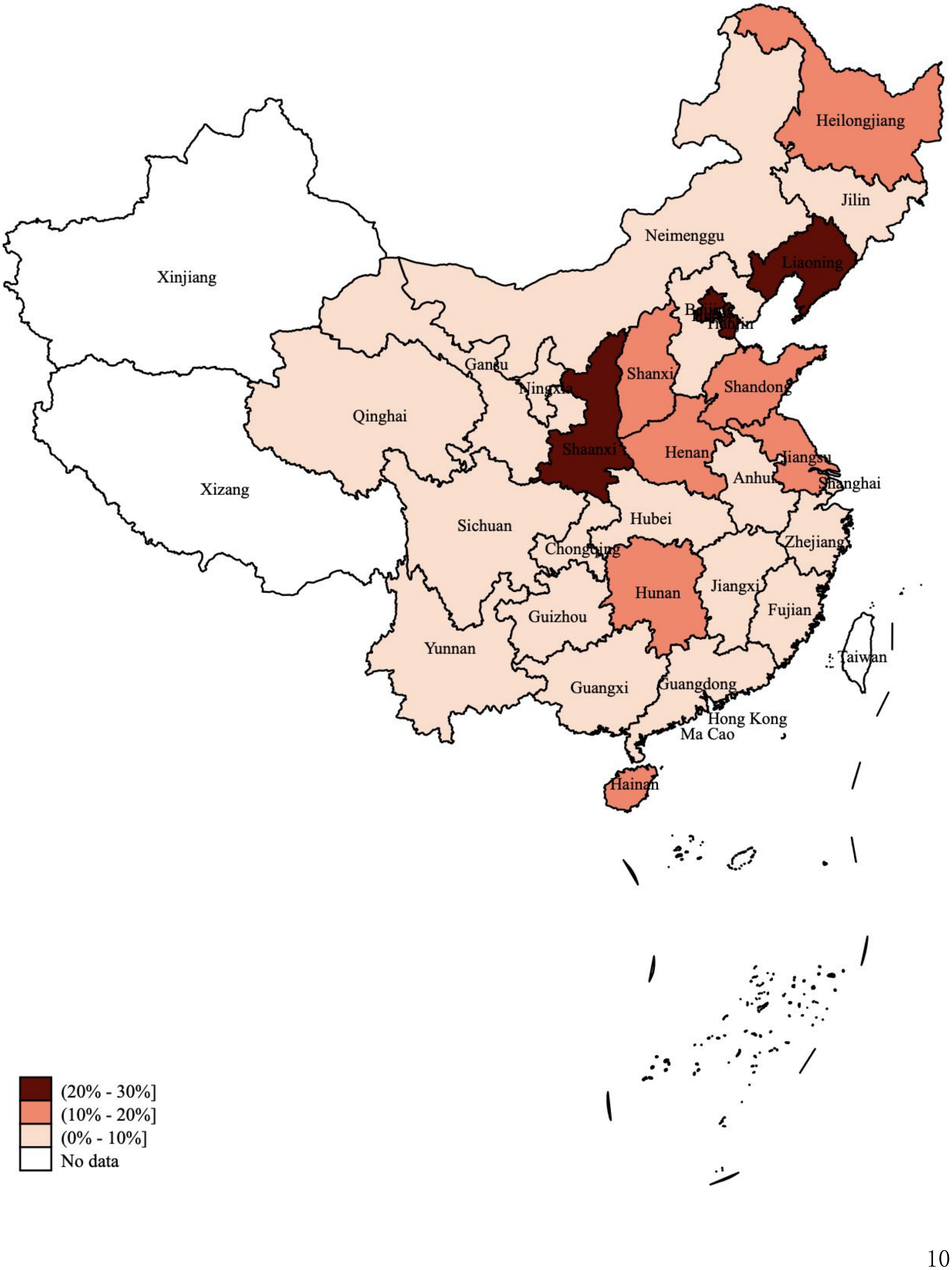

Figure A7. Level and inequalities of % of households with HBPPE (thresehold=10%) by type of kindergarten, maternal education, household net wealth quintile, and current residential area

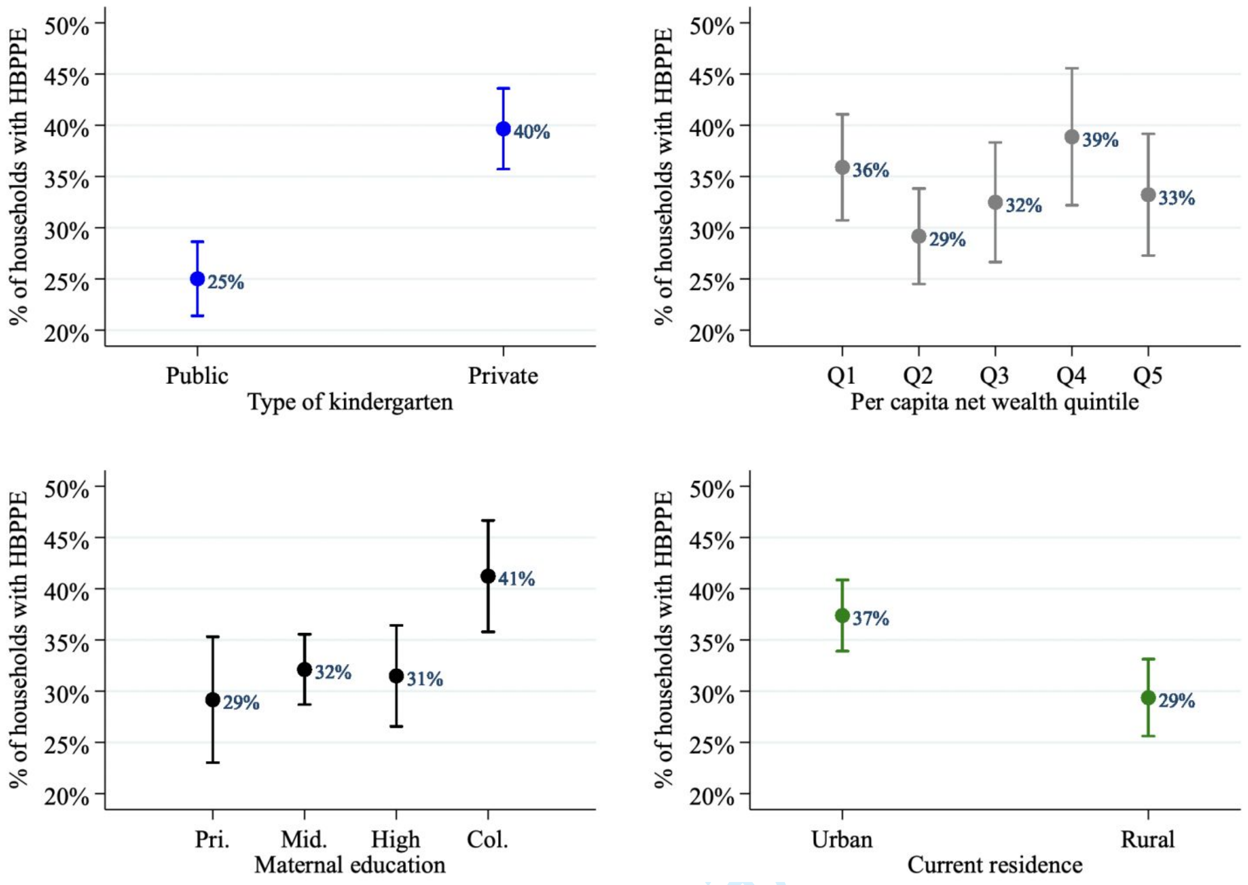

Notes: "Pri.", "Mid.", "High", and "Col." refers to maternal schooling at the primary and below, middle, high, and college and above levels, respectively.

review Only

Figure A8. Level and inequalities of % of households with HBPPE (thresehold=20%) by type of kindergarten, maternal education, household net wealth quintile, and current residential area

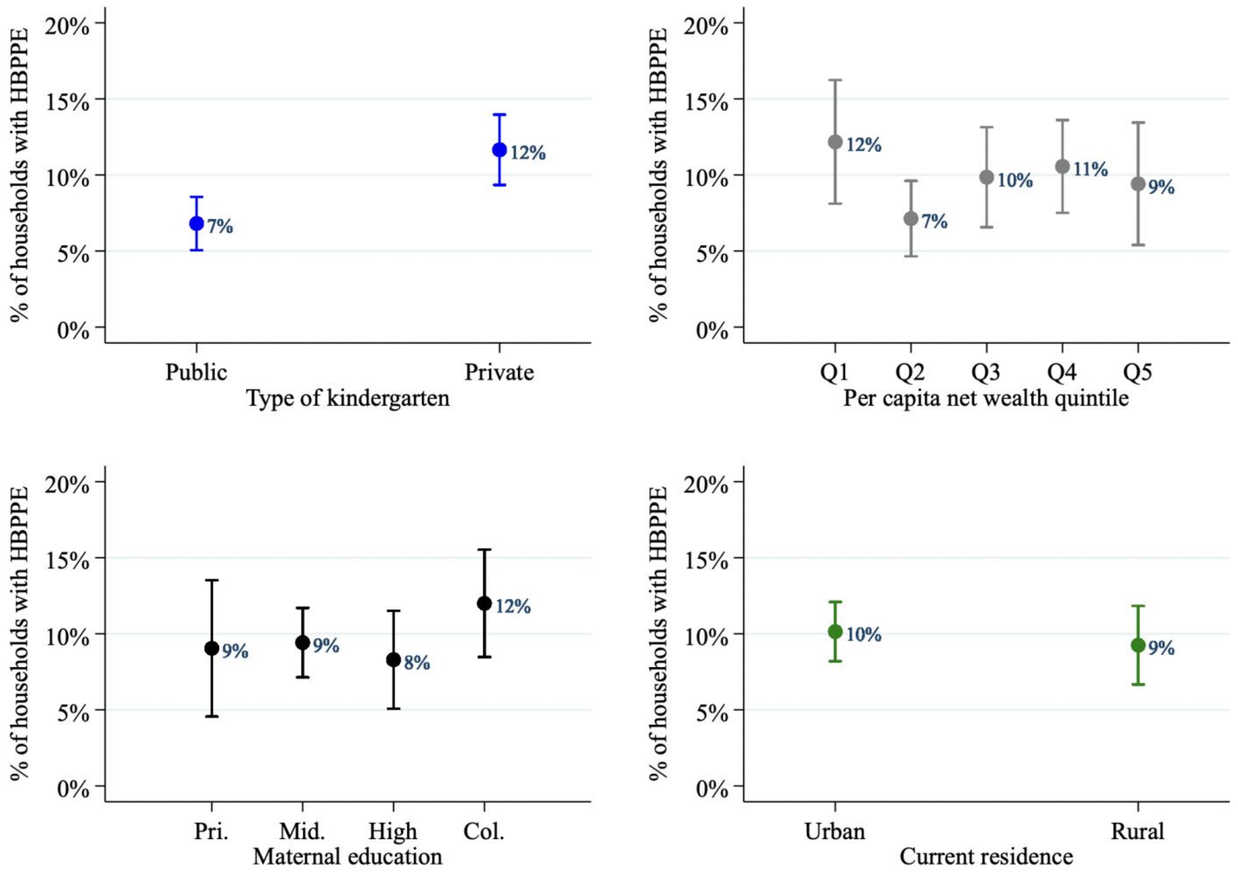

Notes: “Pri.”, “Mid.”, “High”, and “Col.” refers to maternal schooling at the primary and below, middle, high, and college and above levels, respectively.

eview Only

Figure A9. Level and inequalities of % of households with HBPPE (thresehold=7%) by excluding out-school spending from PPE spending

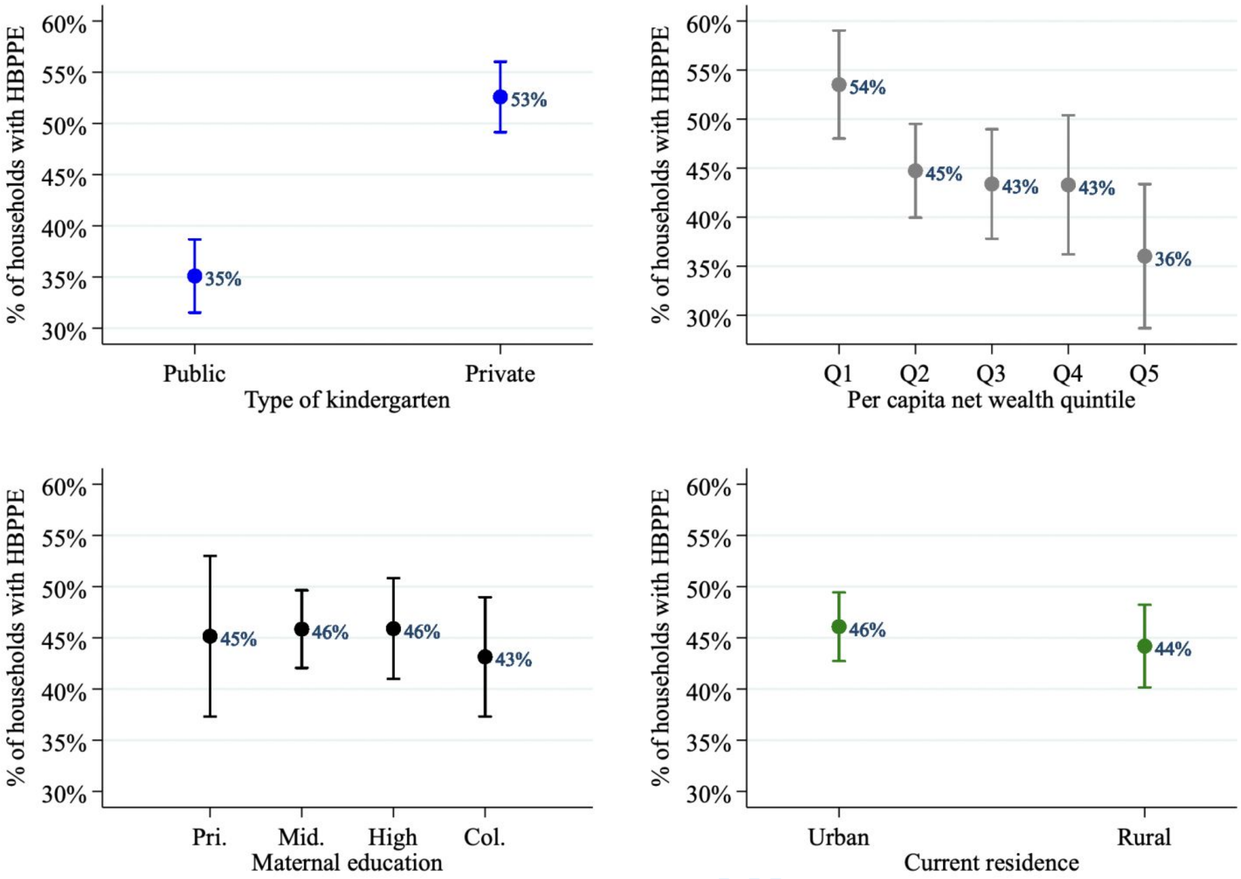

Notes: "Pri.", "Mid.", "High", and "Col." refers to maternal schooling at the primary and below, middle, high, and college and above levels, respectively.

For peer review Only

Figure A10. Level and inequalities of % of households with HBPPE (thresehold=10%) by excluding out-school spending from PPE spending

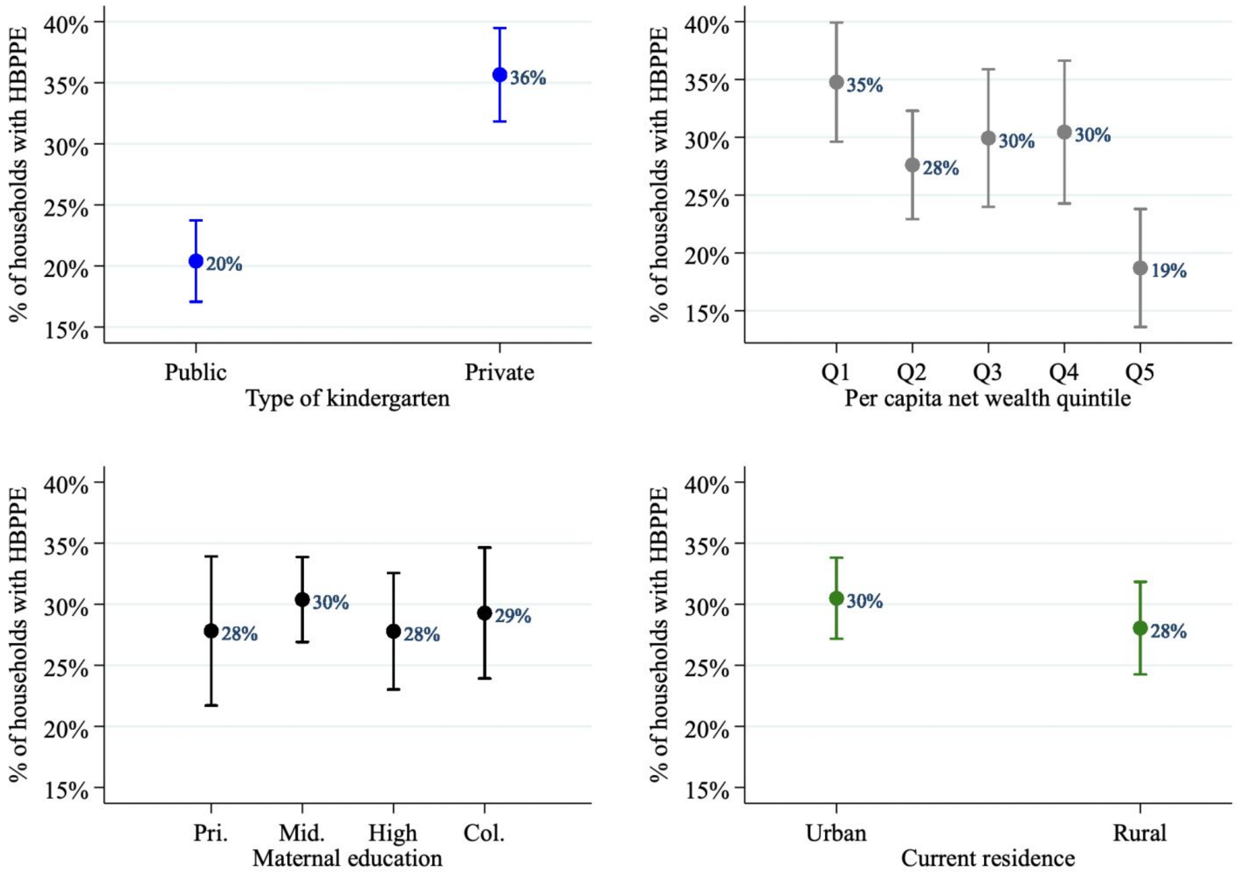

Notes: "Pri.", "Mid.", "High", and "Col." refers to maternal schooling at the primary and below, middle, high, and college and above levels, respectively.

For peer review Only

Figure A11. Level and inequalities of % of households with HBPPE (thresehold=20%) by excluding out-school spending from PPE spending

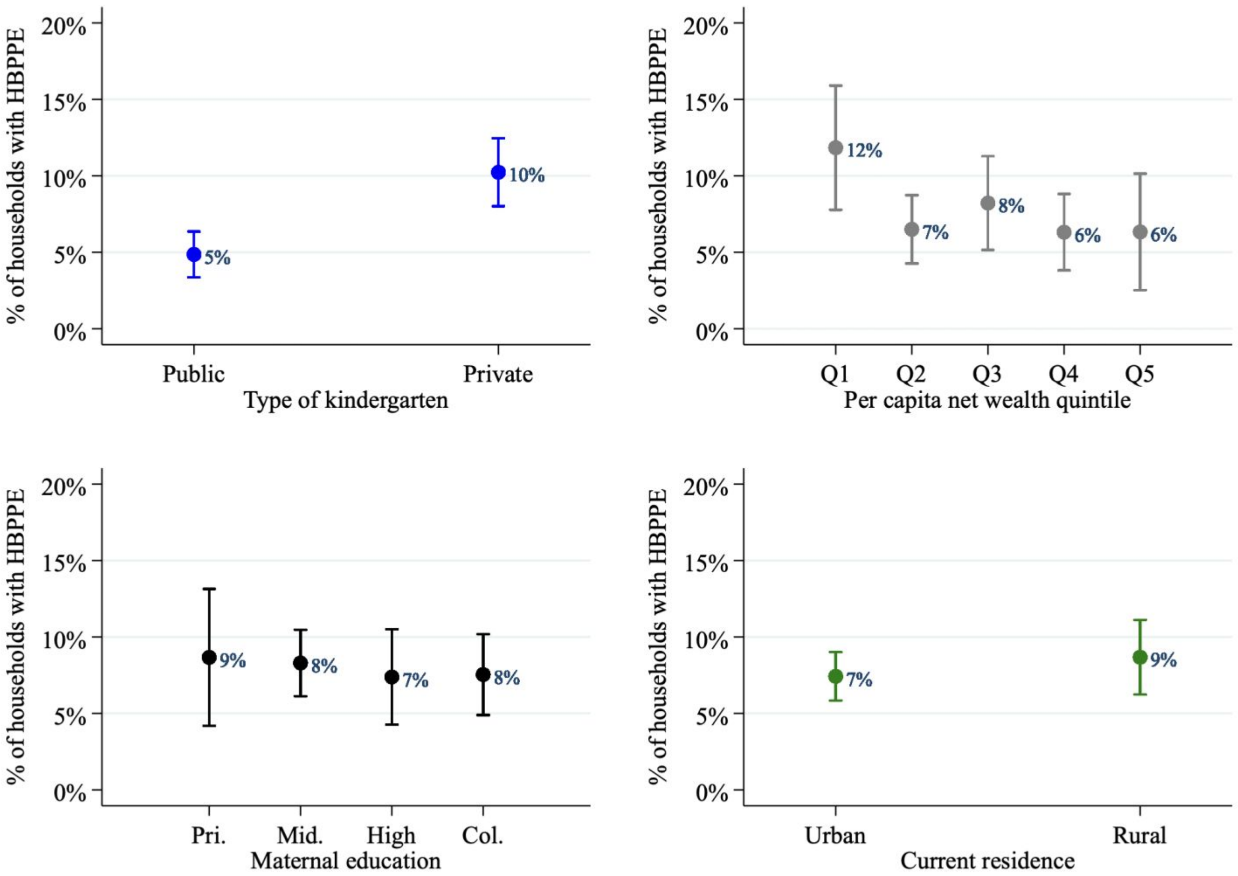

Notes: "Pri.", "Mid.", "High", and "Col." refers to maternal schooling at the primary and below, middle, high, and college and above levels, respectively.

For peer review only

Figure A12. Level of % of household expenditure on PPE across 29 provinces

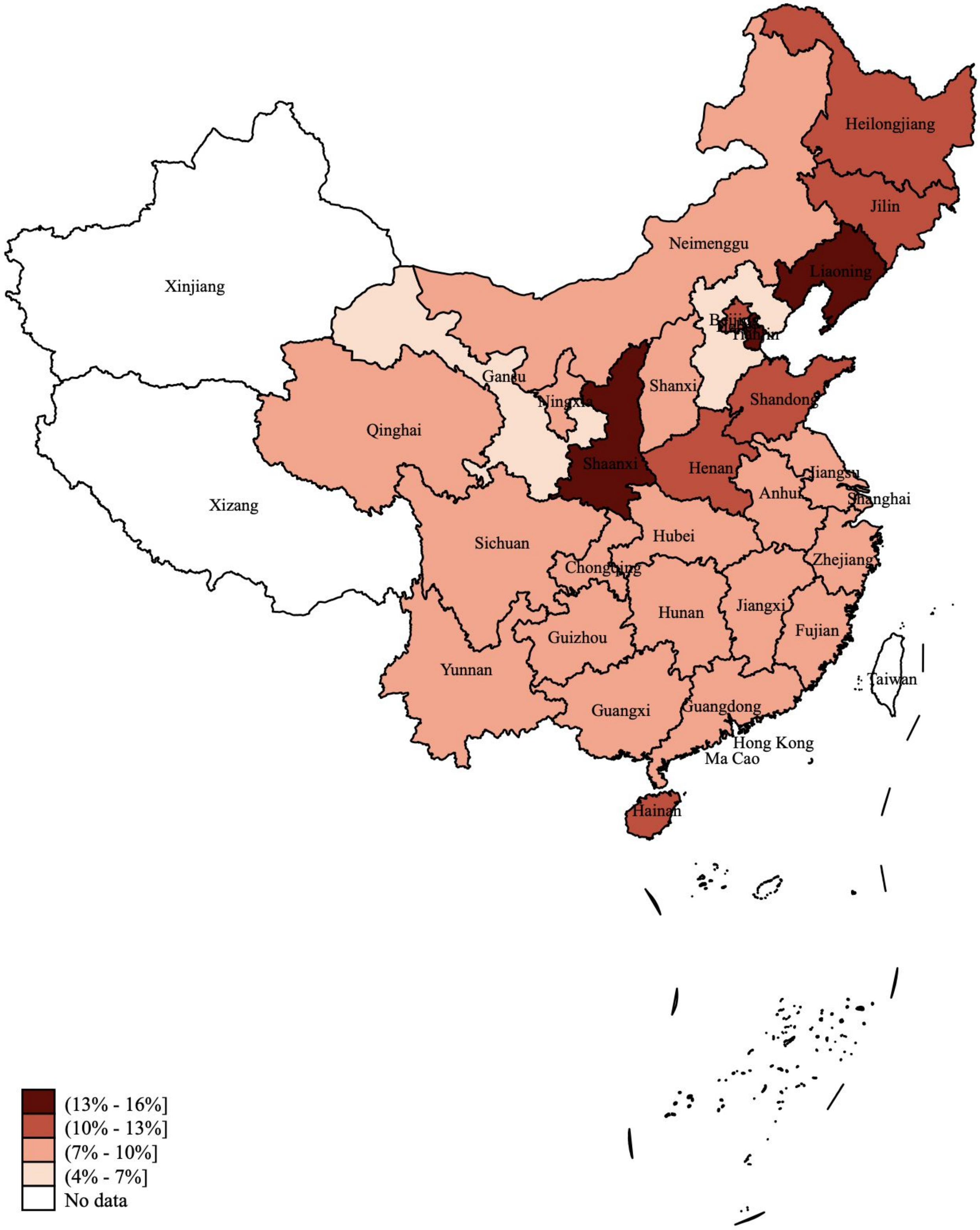

Figure A13. Level and inequalities of % of household expenditure on PPE by type of kindergarten, maternal education, household net wealth quintile, and current residential area

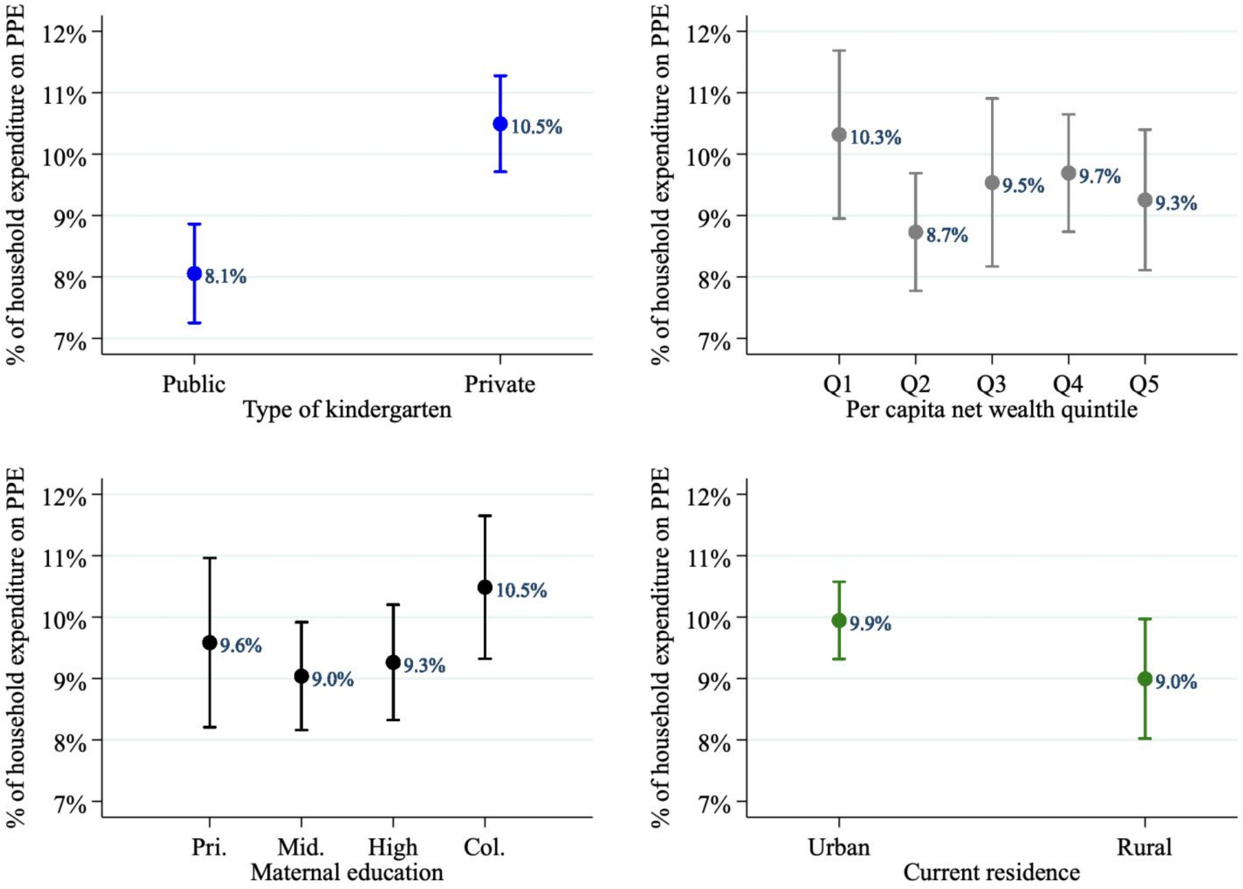

Notes: "Pri.", "Mid.", "High", and "Col." Refers to maternal schooling at the primary and below, middle, high, and college and above levels, respectively.

Figure A14. Level and inequalities of % of household expenditure on PPE by excluding out-school spending from PPE spending

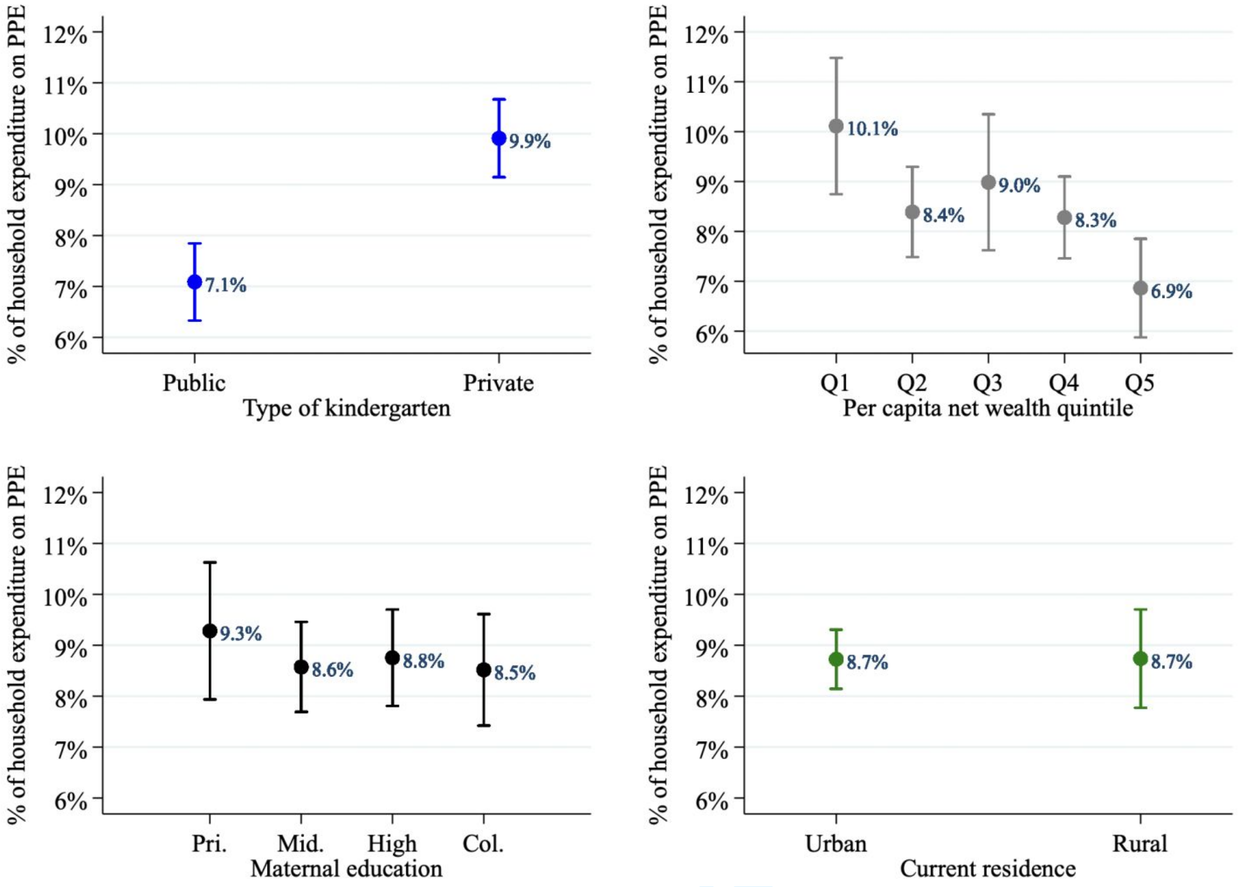

Notes: "Pri.", "Mid.", "High", and "Col." refers to maternal schooling at the primary and below, middle, high, and college and above levels, respectively.

For peer review Only

Confidential: For Review Only

Table A1. Significance testing of inequalities in PPE spending per child using Cumming and Finch's "rule of eye"

	Mean	Lower bound	Higher bound	Margins of error	Proportion overlap			
Gender					vs female			
Male	7437.056	6631.678	8242.434	805.378	1.763			
Female	7238.254	6369.469	8107.039	868.785				
Private school					vs private			
Public	6193.239	5475.135	6911.343	718.104	**-0.244**			
Private	8075.494	7116.165	9034.824	959.330				
Maternal education					vs middle	vs high	vs college	
Primary	3756.339	3163.960	4348.719	592.379	**-0.615**	**-3.341**	**-6.995**	
Middle	5138.580	4673.767	5603.393	464.813	**.**	**-1.940**	**-6.229**	
High	8068.083	7045.833	9090.333	1022.250	**.**	**.**	**-2.314**	
College	13824.870	12178.490	15471.250	1646.381				
Per capita net wealth quintile					vs Q2	vs Q3	vs Q4	vs Q5
Q1	4182.038	3628.280	4735.797	553.759	0.683	**-1.678**	**-4.956**	**-7.985**
Q2	4813.727	4408.062	5219.393	405.666	.	**-1.117**	**-4.816**	**-8.074**
Q3	6812.270	5935.722	7688.817	876.548	.	**.**	**-1.435**	**-5.051**
Q4	10473.690	9218.501	11728.880	1255.188	.	**.**	**.**	**-1.995**
Q5	17007.120	14991.910	19022.340	2015.218				
Current residence					vs rural			
Urban	10433.780	9434.861	11432.700	998.918	**-7.059**			
Rural	4126.709	3733.122	4520.297	393.588				

Notes: we used Cumming and Finch's "rule of eye" to test the statistical significance of inequalities in PPE spending between the two sub-groups (i.e. whether significant difference existed between the two mean estimates). Details for the analytical methods were presented in **Appendix Panel A**. Numbers in bold indicates a proportion overlap that is less than 0.5; that is a significant statistical relationship at the 0.05 level ($p<0.05$)

Confidential: For Review Only

Table A2. Level of % of households with HBPPE (threshold=7%) across 29 provinces and weighted mean difference of the mean between the provincial level and the national level

Province	Level of % of households with HBPPE at the provincial level	Weighted mean difference
Liaoning	76.9%	0.0086
Shaanxi	67.0%	0.0049
Beijing	64.5%	0.0023
Neimenggu	62.5%	0.0024
Henan	61.2%	0.0082
Hainan	60.0%	0.0007
Tianjin	59.9%	0.0012
Hubei	58.5%	0.0039
Heilongjiang	54.4%	0.0014
Hunan	53.8%	0.0022
Jilin	52.7%	0.0007
Guangxi	52.3%	0.0011
Shandong	51.5%	0.0016
Shanxi	49.3%	0.0000
Fujian	48.7%	-0.0002
Guangdong	47.7%	-0.0013
Jiangsu	46.6%	-0.0016
Sichuan	44.4%	-0.0029
Qinghai	43.9%	-0.0002
Jiangxi	42.2%	-0.0023
Chongqing	41.1%	-0.0018
Guizhou	40.9%	-0.0022
Yunnan	40.5%	-0.0030
Ningxia	40.5%	-0.0004
Anhui	39.9%	-0.0043
Shanghai	39.4%	-0.0017
Zhejiang	36.4%	-0.0054
Hebei	35.8%	-0.0073
Gansu	33.1%	-0.0031

*: The measures took zero as the reference, with positive values indicating provincial means higher than the national average, and negative values indicating provincial means lower than the national average.

Confidential: For Review Only

Table A3. Level of % of households with HBPPE (threshold=10%) across 29 provinces and weighted mean difference of the mean between the provincial level and the national level

Province	Level of % of households with HBPPE at the provincial level	Weighted mean difference
Liaoning	63.9%	0.0094
Tianjin	49.5%	0.0018
Beijing	49.1%	0.0024
Shaanxi	48.8%	0.0042
Jilin	47.0%	0.0026
Henan	46.1%	0.0086
Hainan	45.2%	0.0008
Shandong	42.5%	0.0064
Heilongjiang	39.2%	0.0015
Chongqing	36.5%	0.0007
Neimenggu	35.1%	0.0003
Hunan	34.2%	0.0003
Guangdong	33.4%	-0.0001
Fujian	32.0%	-0.0004
Hubei	31.9%	-0.0007
Guangxi	31.6%	-0.0007
Guizhou	30.9%	-0.0007
Shanxi	30.3%	-0.0009
Ningxia	29.7%	-0.0002
Jiangsu	29.3%	-0.0024
Yunnan	27.9%	-0.0020
Sichuan	27.6%	-0.0035
Anhui	25.9%	-0.0035
Shanghai	25.5%	-0.0014
Qinghai	23.6%	-0.0004
Zhejiang	23.1%	-0.0044
Jiangxi	22.7%	-0.0036
Hebei	19.7%	-0.0075
Gansu	19.0%	-0.0028

*: The measures took zero as the reference, with positive values indicating provincial means higher than the national average, and negative values indicating provincial means lower than the national average.

Confidential: For Review Only

Table A4. Level of % of households with HBPPE (threshold=20%) across 29 provinces and weighted mean difference of the mean between the provincial level and the national level

Province	Level of % of households with HBPPE at the provincial level	Weighted mean difference
Liaoning	27.8%	0.0056
Tianjin	23.6%	0.0015
Shaanxi	22.9%	0.0036
Beijing	20.1%	0.0016
Shandong	18.9%	0.0066
Hainan	17.3%	0.0005
Heilongjiang	16.9%	0.0019
Shanxi	12.8%	0.0008
Henan	12.6%	0.0020
Hunan	11.1%	0.0007
Jiangsu	10.2%	0.0003
Yunnan	10.0%	0.0001
Qinghai	9.9%	0.0000
Guangdong	9.3%	-0.0004
Guizhou	8.8%	-0.0002
Jilin	8.4%	-0.0003
Zhejiang	8.3%	-0.0006
Chongqing	6.8%	-0.0006
Neimenggu	6.7%	-0.0005
Hubei	6.4%	-0.0014
Shanghai	5.9%	-0.0007
Guangxi	5.2%	-0.0016
Gansu	5.1%	-0.0009
Ningxia	4.8%	-0.0002
Fujian	4.7%	-0.0014
Sichuan	4.5%	-0.0031
Jiangxi	4.4%	-0.0018
Hebei	4.0%	-0.0031
Anhui	2.9%	-0.0031

*: The measures took zero as the reference, with positive values indicating provincial means higher than the national average, and negative values indicating provincial means lower than the national average.

Confidential: For Review Only

Table A5. Significance testing of inequalities in HBPPE (threshold=7%) using Cumming and Finch's "rule of eye"

	Mean	Lower bound	Higher bound	Margins of error	Proportion overlap			
Private school					vs private			
Public	0.410	0.372	0.448	0.038	**-1.970**			
Private	0.554	0.520	0.588	0.034				
Maternal education					vs middle	vs high	vs college	
Primary	0.463	0.384	0.542	0.079	1.648	1.645	0.890	
Middle	0.484	0.445	0.523	0.039	.	1.952	0.864	
High	0.486	0.436	0.536	0.050	.	.	1.024	
College	0.537	0.483	0.590	0.054				
Per capita net wealth quintile					vs Q2	vs Q3	vs Q4	vs Q5
Q1	0.544	0.490	0.598	0.054	0.667	0.566	0.980	1.383
Q2	0.476	0.427	0.524	0.049	.	1.803	1.817	1.467
Q3	0.465	0.409	0.521	0.056	.	.	1.651	1.330
Q4	0.486	0.425	0.546	0.061	.	.	.	1.673
Q5	0.507	0.439	0.574	0.068				
Current residence					vs rural			
Urban	0.525	0.493	0.557	0.032	**0.168**			
Rural	0.458	0.417	0.499	0.041				

Notes: we used Cumming and Finch's "rule of eye" to test the statistical significance of inequalities in HBPPE between the two sub-groups (i.e. whether significant difference existed between the two mean estimates). Numbers in bold indicates a proportion overlap that is less than 0.5; that is a significant statistical relationship at the 0.05 level (p<0.05)

Confidential: For Review Only

Table A6. Significance testing of inequalities in HBPPE (threshold=10%) using Cumming and Finch's "rule of eye"

	Mean	Lower bound	Higher bound	Margins of error	Proportion overlap			
Private school					vs private			
Public	0.250	0.214	0.286	0.036	**-1.873**			
Private	0.396	0.357	0.436	0.039				
Maternal education					vs middle	vs high	vs college	
Primary	0.292	0.230	0.353	0.062	1.384	1.580	**-0.082**	
Middle	0.321	0.287	0.356	0.034	.	1.851	**-0.053**	
High	0.315	0.266	0.364	0.049	.	.	**0.122**	
College	0.412	0.358	0.467	0.054				
Per capita net wealth quintile					vs Q2	vs Q3	vs Q4	vs Q5
Q1	0.359	0.307	0.411	0.052	0.631	1.380	1.498	1.518
Q2	0.292	0.245	0.338	0.047	.	1.367	**0.288**	1.235
Q3	0.325	0.267	0.383	0.058	.	.	0.979	1.875
Q4	0.389	0.322	0.456	0.067	.	.	.	1.104
Q5	0.332	0.273	0.392	0.059				
Current residence					vs rural			
Urban	0.374	0.339	0.409	0.035	**-0.216**			
Rural	0.294	0.256	0.331	0.038				

Notes: Numbers in bold indicates a proportion overlap that is less than 0.5; that is a significant statistical relationship at the 0.05 level ($p<0.05$)

Confidential: For Review Only

Table A7. Significance testing of inequalities in HBPPE (threshold=20%) using Cumming and Finch's "rule of eye"

	Mean	Lower bound	Higher bound	Margins of error	Proportion overlap			
Private school					vs private			
Public	0.068	0.051	0.086	0.018	**-0.377**			
Private	0.116	0.093	0.140	0.023				
Maternal education					vs middle	vs high	vs college	
Primary	0.090	0.046	0.135	0.045	1.888	1.806	1.261	
Middle	0.094	0.071	0.117	0.023	.	1.591	1.112	
High	0.083	0.051	0.115	0.032	.	.	0.902	
College	0.120	0.085	0.155	0.035				
Per capita net wealth quintile					vs Q2	vs Q3	vs Q4	vs Q5
Q1	0.122	0.081	0.162	0.041	**0.459**	1.368	1.547	1.318
Q2	0.071	0.047	0.096	0.025	.	1.058	0.760	1.298
Q3	0.099	0.066	0.131	0.033	.	.	1.776	1.881
Q4	0.106	0.075	0.136	0.030	.	.	.	1.676
Q5	0.094	0.054	0.134	0.040				
Current residence					vs rural			
Urban	0.101	0.082	0.121	0.019	1.604			
Rural	0.092	0.067	0.118	0.026				

Notes: Numbers in bold indicates a proportion overlap that is less than 0.5; that is a significant statistical relationship at the 0.05 level (p<0.05)

Confidential: For Review Only

Table A8. Level of % of household expenditures on PPE across 29 provinces and weighted mean difference of the mean between the provincial level and the national level

Province	Level of % of household expenditure on PPE at the provincial level	Weighted mean difference
Shaanxi	15.4%	0.00163
Liaoning	14.8%	0.00166
Tianjin	13.1%	0.00040
Shandong	12.9%	0.00242
Beijing	12.6%	0.00048
Hainan	11.9%	0.00016
Henan	11.8%	0.00161
Heilongjiang	11.6%	0.00056
Jilin	10.5%	0.00019
Hunan	9.9%	0.00020
Shanxi	9.6%	0.00003
Hubei	9.3%	-0.00010
Guangdong	9.2%	-0.00024
Neimenggu	9.2%	-0.00006
Jiangsu	9.0%	-0.00026
Guizhou	8.8%	-0.00019
Chongqing	8.6%	-0.00020
Guangxi	8.6%	-0.00033
Fujian	8.3%	-0.00034
Qinghai	8.2%	-0.00006
Jiangxi	8.1%	-0.00046
Sichuan	8.0%	-0.00087
Zhejiang	8.0%	-0.00064
Yunnan	7.9%	-0.00055
Shanghai	7.7%	-0.00031
Ningxia	7.3%	-0.00011
Anhui	7.2%	-0.00106
Hebei	6.4%	-0.00169
Gansu	6.0%	-0.00066

Confidential: For Review Only

Table A9. Multilevel linear regression results of factors associated with logged % of household expenditure on PPE

	Logged % of household spending on PPE	Logged % of household spending on PPE by excluding out-of-school spending from PPE spending
Household net wealth quintile (ref. Quintile 1)		
Quintile 2	-0.134***	-0.128**
Quintile 3	-0.204***	-0.210***
Quintile 4	-0.212***	-0.255***
Quintile 5	-0.304***	-0.479***
Mother's education (ref. Primary school and below)		
Middle school	-0.015	-0.019
High school	0.020	-0.007
College/above	0.058	-0.055
Number of children undertaking PPE	0.499***	0.517***
Household having children in private kindergartens	0.331***	0.381***
Household size	-0.099***	-0.093***
Rural	-0.028	-0.011
Constant	-2.775***	-2.901***
N	2949	2935
ICC (Province)	0.033	0.036
ICC (County | Province)	0.150	0.166

Notes: * p<0.10, ** p<0.05, *** p<0.01. We took the natural logarithm of % of household expenditure on PPE to ensure the variable close to a normal distribution. Of note, because 142 out of 3,111 observations reported zero value for this variable, taking log generated 142 missing values. We adopted a multilevel linear model for estimation.

Confidential: For Review Only

Table A10. Beta regression results of factors associated with % of household expenditure on PPE

	% of household spending on PPE (Odds ratio)	% of household spending on PPE by excluding out-school spending from PPE spending (Odds ratio)
Household net wealth quintile (ref. Quintile 1)		
Quintile 2	0.902**	0.904**
Quintile 3	0.842***	0.840***
Quintile 4	0.862***	0.833***
Quintile 5	0.837***	0.723***
Mother's education (ref. Primary school and below)		
Middle school	1.059	1.054
High school	1.100**	1.082
College/above	1.151***	1.047
Number of children undertaking PPE	1.446***	1.473***
Household having children in private kindergarten	1.319***	1.384***
Household size	0.918***	0.925***
Rural	0.947*	0.961
N	2949	2935

Notes: * p<0.10, ** p<0.05, *** p<0.01. We adopted a beta regression for estimation, which is appropriate for modelling proportional outcome variables that are greater than 0 and less than 1. Of note, because 142 out of 3,111 observations reported zero values for this variable, performing a beta regression excludes these observations with zero values.

Confidential: For Review Only

Table A11. Multilevel logit regression results of factors associated with household status on HBPPE by setting household net wealth as a continuous variable

	HBPPE_7% (Odds ratio)	HBPPE_10% (Odds ratio)	HBPPE_20% (Odds ratio)
Log (Household net wealth)	0.870***	0.855***	0.873***
Mother's education (ref. Primary school and below)			
Middle school	1.084	1.012	0.733
High school	0.967	1.035	0.794
College/above	1.164	1.308	1.031
Number of children undertaking PPE	3.363***	3.433***	3.279***
Household having children in private kindergarten	1.962***	2.018***	1.900***
Household size	0.796***	0.757***	0.805***
Rural	0.824*	0.886	1.151
N	2995	2995	2995
ICC (Province)	0.022	0.033	0.062
ICC (County | Province)	0.117	0.112	0.204

Notes: * p<0.10, ** p<0.05, *** p<0.01. Of note, household net wealth was derived by calculating a household' assets minus its debts, leading to 97 household observations that had negative net wealth values. Taking logarithm of household net wealth generated 97 missing values for this variable.

Confidential: For Review Only

Table A12. Multilevel linear regression and Beta regression results of factors associated with % of household expenditure on PPE by setting household net wealth as a continuous variable

	Logged % of household spending on PPE (Multilevel linear regression)	% of household spending on PPE (Beta regression)
Log (Household net wealth)	-0.065***	-0.041***
Mother's education (ref. Primary school and below)		
Middle school	-0.019	0.041
High school	0.030	0.093*
College/above	0.074	0.146***
Number of children undertaking PPE	0.497***	0.377***
Household having children in private kindergarten	0.336***	0.274***
Household size	-0.099***	-0.085***
Rural	-0.025	-0.048
N	2861	2861

Notes: * p<0.10, ** p<0.05, *** p<0.01.

Confidential: For Review Only

Table A13. Multilevel logit regression results on factors associated with household status on HBPPE defined with various thresholds, children aged 3-6 years only

	HBPPE_7% (Odds ratio)	HBPPE_10% (Odds ratio)	HBPPE_20% (Odds ratio)
Household net wealth quintile (ref. Quintile 1)			
Quintile 2	0.801*	0.730**	0.636**
Quintile 3	0.534***	0.559***	0.561**
Quintile 4	0.596***	0.677**	0.588**
Quintile 5	0.656**	0.552***	0.597**
Mother's education (ref. Primary school and below)			
Middle school	1.129	0.945	0.606**
High school	0.976	1.027	0.795
College/above	1.062	1.189	0.964
Number of children undertaking PPE	3.611***	3.692***	3.336***
Household having children in private kindergarten	1.901***	2.086***	1.897***
Household size	0.782***	0.752***	0.820***
Rural	0.824**	0.920	1.142
N	2538	2538	2538
ICC (Province)	0.011	0.022	0.058
ICC (County | Province)	0.090	0.102	0.186

Notes: * $p<0.10$, ** $p<0.05$, *** $p<0.01$.

Confidential: For Review Only

BMJ Paediatrics Open

Assessing household financial burdens for preprimary education and associated socioeconomic inequalities – a case study in China

Journal:	*BMJ Paediatrics Open*
Manuscript ID	bmjpo-2023-001971.R2
Article Type:	Original research
Date Submitted by the Author:	29-Jun-2023
Complete List of Authors:	Wei, Yi; Peking University, China Institute for Educational Finance Research<br>Liu, Kai; Renmin University of China, School of Labor and Human Resources<br>Kang, Le; Nanjing University, Institute of Education<br>Behrman, Jere; University of Pennsylvania, Department of Economics<br>Richter, Linda; University of the Witwatersrand Johannesburg, DSI-NRF Centre of Excellence in Human Development<br>Stein, Alan; Oxford University, Department of Psychiatry<br>Song, Yingquan; Peking University, China Institute for Educational Finance Research<br>Lu, Chunling; Brigham and Women's Hospital, Division of Global Health Equity; Harvard Medical School, Department of Global Health and Social Medicine
Keywords:	Growth

SCHOLARONE™
Manuscripts

I, the Submitting Author has the right to grant and does grant on behalf of all authors of the Work (as defined in the below author licence), an exclusive licence and/or a non-exclusive licence for contributions from authors who are: i) UK Crown employees; ii) where BMJ has agreed a CC-BY licence shall apply, and/or iii) in accordance with the terms applicable for US Federal Government officers or employees acting as part of their official duties; on a worldwide, perpetual, irrevocable, royalty-free basis to BMJ Publishing Group Ltd ("BMJ") its licensees and where the relevant Journal is co-owned by BMJ to the co-owners of the Journal, to publish the Work in this journal and any other BMJ products and to exploit all rights, as set out in our licence.

The Submitting Author accepts and understands that any supply made under these terms is made by BMJ to the Submitting Author unless you are acting as an employee on behalf of your employer or a postgraduate student of an affiliated institution which is paying any applicable article publishing charge ("APC") for Open Access articles. Where the Submitting Author wishes to make the Work available on an Open Access basis (and intends to pay the relevant APC), the terms of reuse of such Open Access shall be governed by a Creative Commons licence – details of these licences and which Creative Commons licence will apply to this Work are set out in our licence referred to above.

Other than as permitted in any relevant BMJ Author's Self Archiving Policies, I confirm this Work has not been accepted for publication elsewhere, is not being considered for publication elsewhere and does not duplicate material already published. I confirm all authors consent to publication of this Work and authorise the granting of this licence.

Confidential: For Review Only

Assessing household financial burdens for preprimary education and associated socioeconomic inequalities – a case study in China

Yi Wei, Kai Liu, Le Kang, Jere R. Behrman, Linda M. Richter, Alan Stein, Yingquan Song, Chunling Lu

Abstract

Background Providing young children with universal access to preprimary education (PPE) is considered a powerful tool for human-capital development and eliminating the intergenerational transmission of poverty. To remove household financial barrier for achieving universal PPE, this study proposed a measure to identify households incurring "heavy financial burdens from paying for PPE" (HBPPE) and conducted a case study in China.

Methods Using nationally representative data in 2019, we estimated percentage of households with HBPPE (spent 7% or more of their total annual expenditure) and associated socioecnomic inequaities. We also applied a three-level logit-regression model to investigate the factors associated with the probabilities of households incurring HBPPE.

Results Half of the sampled households spent 7% or more of their expenditures on PPE. Households in the lowest wealth quintile (54%) or households with children attending private PPE (55%) had higher percentages of HBPPE than households in other wealth quintiles (e.g., 51% in the highest wealth quintile) or households with children attending public kindergartens (41%). Logit-regression analysis shows that the poorest households and households with children attending private kindergarten were more likely to incur HBPPE than their counterparts.

Conclusion To ensuring universal access to PPE in China, future policy should consider increasing the enrollment of children from low-income families in public kindergartens and increasing governmental investments in low-income households by subsidizing children attending PPE.

Keywords: Access to Kindergarten; Preprimary education, Household financial burden; Socioeconomic inequality

Confidential: For Review Only

WHAT IS ALREADY KNOWN ON THIS TOPIC

- Significant socioeconomic inequalities in preprimary education (PPE) attendance were observed in many low- and middle-income countries (LMICs).
- Empirical evidence on household financial barrier to PPE is very limited in LMICs. In China, disadvantaged households (e.g., low-income, low maternal schooling) paid larger share than their counterparts for sending children to preschool. Little, however, is known on how many households suffered from heavy financial burden from paying for PPE.

WHAT THIS STUDY ADDS

- This study proposed a measure to identify households incurring "heavy financial burdens from paying for PPE" (HBPPE) and conducted a case study in China using nationally representative data.
- Findings showed that almost 50% of the sampled households incurred HBPPE (spent over 7% or more of their annual expenditures on PPE), with the poorest households and households in which children attended private kindergarten more likely to incur HBPPE than their counterparts.

HOW THIS STUDY MIGHT AFFECT RESEARCH, PRACTICE OR POLICY

- This study proposed a measure on HBPPE that could be used for similar research in understanding a country's progress in eliminating financial barrier to universal coverage of PPE.
- Findings of this study could inform policymakers and other stakeholders in China on who suffered HBPPE (e.g., the poorest households or households with children attending private kindergarten) and what could be potentially effective policy instruments in promoting equal access to PPE.

1. Introduction

Preprimary education (PPE) has important influences on the development of cognitive and non-cognitive skills of children, especially for those from disadvantaged groups.[1] The high return on investment in PPE makes it an important strategy for strengthening national human capital and reducing inequality and the intergenerational transmission of poverty.[2-8] Quality PPE for all girls and boys has been included in the 2030 Sustainable Development Goals. [9]

Eensuring universal access to quality PPE requires sufficient investment and effective design of cost-sharing mechanisms between governments and households. In many low- and middle-income countries (LMICs), government financial support to PPE remains rather limited,[10-12] making it difficult for the poor households accessing PPE. In Nigeria, for example, less than 10% of the poorest young children attended PPE in 2010, compared to approximately 80% of the richest young Nigerian children.[13]

China has the second-largest child population in the world and has set a policy goal of universal coverage of PPE in 2010.[14] Since then, public expenditures on PPE have increased rapidly from CNY24.44 billion (1.67% of governmental educational spending) in 2010 to CNY 200.8 billion (5.01% of governmental educational spending) in 2019 (**Figure A1 in the appendix**).[15] The gross-enrollment rate for three-year PPE also grew rapidly from 46.1% in 2000 to 83.4% in 2019 (**Figure A2 in the appendix**).[15]

PPE in China is not free. Households have to pay tuition fees that vary greatly by type of kindergarten, public or private. Public kindergartens are essentially "club goods", which are the extension of the welfare system in the era of planned economy, as a benefit provided by organizations to their "employees". These public kindergartens are not open to the public and have certain requirements for admission, for example, children of employees of governments, universities, or state-owned enterprises, etc. children from more-advantaged groups are more

For peer review only

likely to enter this kind of public kindergarten with relatively low fees and higher quality.[16][17] Currently, government spending on PPE mainly goes to these public kindergartens, especially the kindergartens run by educational departments and other governmental agencies.

Private kindergartens have become an important part of China's PPE system in recent years, with 56% of young children enrolled in private kindergarten in 2019.[15] The majority of private kindergartens have lower quality and are more expensive than public kindergartens. They are used by children who do not have access to public ones. The major share (85%) of the funding in private kindergartens came from tuition fees contributed by households, compared to 25% in public kindergartens.[18]

Limited empirical evidence has shown that disadvantaged households usually paid larger share of their income on PPE than their counterparts in China.[12][19][20] However, little is known regarding how many households suffered from heavy financial burdens from paying for PPE (HBPPE). To fill in this knowledge gap and help policy makers identify those who need support most, this study defined HBPPE using the approach adopted by the World Bank (WB) and the World Health Organization (WHO) in defining catastrophic household health spending. [21][22] For example, the WB defines a household having catastrophic health spending if it spends 10% or more of its total annual expenditures on health. The 10% cut-off has been widely used as an important policy measure for tracking progress towards universal health coverage. This study proposes a similar threshold for HBPPE so as to better understand household financial burdens for paying for PPE and hold governments accountable for their promise of "quality PPE for all girls and boys". If 10% of a household's spending on health has been accepted as "catastrophic" to the household, the same definition could be applied to PPE payments too. We therefore proposed a measure to identify households incurring HBPPE and conducted a case study in China. Using the 2019 China Household Finance Survey (CHFS), we assessed the national- and provincial-level prevalence of HBPPE and associated socioeconomic and inequalities.

2. Methods

We used the CHEERS reporting guidelines in writing of this paper.[23]

Ethical statement: We used a secondary data that has been published and publicly accessible.[24] Identifiers for individuals and families have been removed for public use. Users cannot identify individuals and households from this survey dataset.

This study is an observational study based on public dataset instead of a clinical study. Therefore, a patient and public involvement (PPI) statement is not applicable to this study.

2.1. Defining households with heavy financing burdens for paying for PPE (HBPPE)

There is no available measure for catastrophic household PPE spending. Following the practice of the WB and the WHO in defining catastrophic household health spending, we defined a household incurring HBPPE if it spends 7% (10%, or 20%) or more of its total annual expenditures on PPE. The selection of these thresholds was based on the definiton of catastrophic health spending as well as a current federal benchmark for households receiving

Confidential: For Review Only

subsidies on PPE proposed by the Office of Child Care under the U.S. Department of Health and Human Services, stating that households receive subsidies if their out-of-pocket spending on PPE exceed 7% (previously 10%) of households' incomes.[25] Though the threshold of 7% comes from the US policy on subsidizing PPE, we think that it could also be applied to the context of China as the cutoff is in terms of proportion of income rather than actual income. If households that spent 7% or more on PPE need to be subsidized in high-income countries, it should also be the case in LMICs considering that households in LMICs have less capacities to pay compared to those in high-income countries.

2.2. Data and samples

We used nationally and provincially representative data from the 2019 China Household Finance Survey (CHFS) that collects information on household assets, income, wealth, consumption, as well as socioeconomic and demographic characteristics of the sampled households and their members.[25] The CHFS employs a stratified three-stage random sample design (details in **Chapter 1 and Figure A3 of the Appendix**). The 2019 CHFS data is publicly accessible and covers 29 provinces (excluding Tibet, Xinjiang, Hong Kong, and Macau), 345 cities/districts/counties, 34,643 households, and 107,008 individuals. Our study focused on households with children attending kindergarten, including those under age 3 or over age 6 who still attended kindergarten (18.2% of the sample), because the main purpose of this study is to identify households incurring heavy financial burdens from paying for PPE. The final sample includes 3,371 young children attending kindergartens, from 3,111 households (9% of the total sample), who reported available PPE expenditure data.

2.3. Measuring households with HBPPE

To assess if household suffered from HBPPE, we calculated estimates of household spending on PPE and total expenditures in the previous 12 months. Caregivers reported household expenditure on kindergarten over the 12 months prior to the survey. The expenditure can be categorized into two groups: (i) fees charged by schools (in-school expenditures) including tuition fees, books and materials, uniforms, meals, transport, and accommodation; (ii) fees on out-of-school education (out-of-school expenditure) including after-school tutoring (academically focused), extracurricular activities (e.g., learning piano or sports), and home-learning materials (e.g., books, computers). To estimate the sensitivity of the findings to the definition of PPE spending, we excluded out-of-school spending from PPE spending in an alternative analysis.

The CHFS includes a series of questions on household consumption over the 12 months prior to the survey that cover spending on food, clothing, housing, daily necessities and service, health care, transportation and communication, education and entertainment, and others (e.g. legal service). We obtained the annual household expenditure by summing spending on these items. For each household, we calculated the percentage of household total expenditure devoted to PPE. Based on the cutoff percentage value (7%, 10%, or 20%), we then constructed a dichotomous variable indicating a household's status of HBPPE: if a household spent more than 7% (10% or 20%) of its total expenditure on PPE, we classified the household as experiencing HBPPE.

Confidential: For Review Only

2.4. Measuring socioeconomic disparities in HBPPE

We assessed the disparities in the percentage of households with HBPPE by household economic status (five wealth quintiles), maternal schooling (primary school or below, middle school, high school, and college or above), residential area (rural vs. urban), and type of kindergarten (public vs. private). Disparity in this study was quantified as the absolute differences between the two sub-groups. Details on constructing these variables are presented in **Chapter 2 of the Appendix**.

2.5. Statistical Analysis

We first performed a descriptive analysis by assessing the level and socioeconomic inequalities in PPE spending per child and percentages of households with HBPPE. All estimates were adjusted for sampling weights (individual and household weights), primary sampling unit (county), and stratification variable (province). We estimated the statistical significance of inequalities in PPE spending and % of households with HBPPE between the two sub-groups (e.g. rural vs urban) (details in **Chapter 3 in the Appendix**).[26]

Considering the large variation in economic development and geographical situations across the provinces, we assessed the provincial-level disparities in the level of PPE spending per child and % of households with HBPPE across the 29 provinces, using the gap between the weighted mean of each province from the national mean (details in **Chapter 4 of the Appendix**).

To identify the factors associated with the probabilities of a household incurring HBPPE, we used a three-level logit regression model with random intercepts by province (level 3) and county (level 2), controlling for potential clustering effects at the province and county levels (Equation 1).

$$Logit\ (Y_{ijk}) = \beta_0 + \boldsymbol{\beta X_{ijk}} + u_{0k} + v_{0jk} + \varepsilon_{0ijk} \quad (1)$$

where Logit(Y_{ijk}) represents the probability of incurring HBPPE in the *i*th household who lives in county *j* and province *k*. **β** is a vector of coefficients for $\mathbf{X}_{ijk}$ which is a vector of variables with individual or household characteristics, including the number of children undertaking PPE, children attending private kindergarten, household size, maternal schooling, wealth quintile, and residential area. u_{0k} and v_{0jk} represent between-province random variations and between-county/within-province random variations, respectively. ε_{0ijk}represent random errors. Summary statistics for these variables are presented in **Table 1**.

Regression analyses were conducted using three different thresholds for HBPPE: 7%, 10%, and 20%. To further test the sensitivity of the findings, we also used proportion of household consumption on PPE as an outcome variable and conducted descriptive and regression analysis, as shown in **Chapter 5 of the Appendix**. In addition, we also used continuous household net wealth instead of net wealth quintiles as a predictor and conducted regression analysis. The STATA program (Version 16.1) was used in analyses.

Confidential: For Review Only

3. Results

Among all the children attending kindergarten, 47.0% were girls, and 56.0% attended private kindergarten. Among the total of 3,111 households, 35.2% lived in rural areas; 11.9% or 21.0% were in the highest or lowest wealth quintile, respectively; 24.8% of the mothers held college degrees or higher, whereas over 57.2% less than a high-school diploma (**Table 1**).

3.1. Level and inequalities in household PPE spending per child

On average, PPE spending per child at the national level was CNY 8,220 (**Table 1)**, ranging from CNY4,070 in Qinghai province to CNY 23,348 in Beijing (**Figure 1 and Table 2**). Well-developed provinces had much higher PPE spending per child than the under-developed central and western provinces (**Table 2**).

For households sending children to private kindergartens, or in urban areas, or with college-educated mothers, or from the highest wealth quintile, their PPE spending per child was significantly higher than that of their counterparts (**Figure 2**). For example, PPE spending per child at the private kindergartens was approximately 30% higher higher than that at public kindergartens (CNY8,075 vs. CNY6,193). PPE spending per child in the richest households was four times more than that in the poorest households (CNY17,007 vs. CNY4,182) (statistical testing in **Appendix Table A1**).

When excluding out-of-school expenditure from PPE spending, the main results remain robust (**Appendix Figure A4)**.

3.2. Level and inequalities in the percentage of HBPPE

Approximately 49.5% of households at the national level spent more than 7% of their total annual expenditure on paying for PPE, our initial threshold for HBPPE. This percentage reduced to 34.2% or 9.8% respectively if the threshold was set at 10% or 20% (**Table 1**). Provinces in the Northeastern and Middle areas of China had higher percentages of HBPPE than other areas, with Liaoning province (76.9%) the highest (**Figure 3, Table A2**). When using alternative thresholds (10% or 20%), the percentage of households with HBPPE in each province was reduced, but the pattern across the provinces remained stable (**Appendix Figures A5 and A6 and Tables A3 and A4)**.

In terms of socioeconomic inequalities (**Figure 4**), % of HBPPE was higher among those households sending children to private kindergartens (55%) than those to public kindergartens (41%). The disparity remained significant when using alternative thresholds (**Appendix Figures A7 and A8**, **Appendix Tables A5 -A7)** or when excluding out-of-school spending from PPE spending (**Appendix Figures A9-11**). % of HBPPE among the richest households was significantly lower than that among the poorest households when using threshold of 7% or 10%, after excluding out-of-school spending from PPE spending.

Confidential: For Review Only

3.3. Factors associated with household HBPPE

Regression results (**Table 3**) have identified the following factors associated with HBPPE, across the three thresholds: households in the higher wealth quintiles or with smaller size were less likely to incur HBPPE than their counterparts; households sending children to private kindergarten or with more children in kindergarten were more likely to incur HBPPE than their counterparts.

Results from the sensitivity analysis using proportion of household spending on PPE as outcome variables are presented in the Appendix (**Figures A12-A14**, **Tables A8-A10**). We used both a multilevel linear regression and a beta regression for estimation. As **Tables A9 and A10** show, the results are consistent with the main regression results.

Results from the sensitivity analysis using continuous household net wealth as a predictor are presented in the Appendix (**Tables A11 and A12**). We used a multilevel logit regression, multilevel linear regression and a beta regression for estimation. As **Tables A11 and A12** show, the results are consistent with the main regression results.

4. Discussion

This study proposed a measure to identify households incurring "heavy financial burdens from paying for PPE" (HBPPE) and applied the measure to the data in China. Our findings showed that, in China, approximately 50% (34%, 10%) of the households with young children attending kindergarten suffered from HBPPE, when a threshold of 7% (10%, 20%) of their annual expenditures on PPE was applied. Households from provinces in the Northeastern and Middle Regions had higher percentages of HBPPE on average than those from other areas. Holding everything else constant, households in the lowest-wealth quintile or sending children to private kindergarten incurred the higher probability of HBPPE than their counterparts.

These findings are consistent with previous studies that indicate variation of PPE spending by household income levels and type of kindergartens. [12,19,20] Our study has further identified significant disparities in household with HBPPE, and found that households with children attending private kindergartens had an increase of 88%-105% (across the three thresholds) in the odds of incurring HBPPE compared to households with children attending public kindergartens. The richest households could have a reduction of 37%-50% in the odds of incurring HBPPE compared to the poorest households. The finding of regional disparities in HBPPE is consistent with empirical evidence regarding the slowdown of economic development in the Northeastern and Middle Regions and their lower percentage of public spending on compulsory education than other regions and on PPE in particular. [27-30]

To ensure universal PPE, the Chinese government has initiated the policy of increasing the supply of public kindergartens and regulating the tuition fees of private kindergartens. As shown in our study, even with the expansion of public kindergartens in recent years, more than half of young children still had to go to expensive private kingdergarten. To reduce the financial burden of PPE for households, the Chinese government needs to continue increasing the public kindergartens and make them accessible to everyone. Meanwhile, providing PPE subsidies to

Confidential: For Review Only

low-income households, especially those with children going to the private kindergartens, is necessary for ensuring the poor children having access to affordable PPE.

This study has several limitations. First, the study only included children enrolled in kindergarten at the time of the survey. Children who were not able to attend kindergarten were not included in the analysis sample and they were more likely to live in poor or remote rural areas with limited or even no access to kindergarten. Excluding those children could lead to an underestimation of the probability of HBPPE among the poorest or rural households. Second, the study only used cross-sectional data. With a new wave of survey data available in the future, we will be able to identify the impact of related governmental policies on HBPPE over time. Third, the study used three thresholds to measure households with HBPEE. However, there is no information from households on what percentage of their total spending on PPE would be considered affordable. Fourth, when applying a threshold of HBPPE to households with different income levels, it may underestimate the burden of PPE borne by low-income households because they have less capacity to pay compared to those in the middle or upper quintiles.

Despite the limitations, this study proposed a measure that can be used by researchers to identify households with financial barrier for accessing PPE in LMICs. Using this measure in China, the study provides solid evidence to inform policy makers on who suffered HBPPE and what could be potentially effective policy instruments in promoting equal access to PPE. Future PPE policy in China should consider establishing a financial-aid system on PPE for the children who need it most and expanding the enrollment of children from disadvantaged households in the public kindergarten system.

Source of funding
This work was supported by Ministry of Educaiton in China (7122900028), UNICEF China Office (8430500872), and the UKRI GCRF Harnessing the Power of Global Data to Advance Young Children's Learning and Development (ES/T003936/1).

Conflict of interest
The authors have declared no competing interests.

Authors' contribution
YW was responsible for survey coordination , conducted data analysis, and wrote the first draft of the manuscript; LK drew the map that demonstrates the sample distribution in the survey as well as the financial burdens in PPE at provincial level. KL conducted data analysis on inequalities and regressions and took responsibility for the integrity of data analysis. YS was the

Confidential: For Review Only

PI for expenditures of PPE, survey design, initiated the study, supervised YW's work. CL conceptualized the study, designed the analysis plan, and mentored data analysis. All authors have made contributions to data interpretation and manuscript writing. All authors approve the manuscript for submission.

Data sharign statement

Data from the China Household Finance Survey (CHFS) is available at the website of the Survey and Research Center for China Household Finance, at https://chfs.swufe.edu.cn/
The analysis code is available from the corresponding author upon request.

Confidential: For Review Only

References

1. Black, M. M., Walker, S. P., Fernald, L., Andersen, C. T., DiGirolamo, A. M., Lu, C., McCoy, D. C., Fink, G., Shawar, Y. R., Shiffman, J., Devercelli, A. E., Wodon, Q. T., Vargas-Barón, E., Grantham-McGregor, S., & Lancet Early Childhood Development Series Steering Committee. (2017). Early childhood development coming of age: science through the life course. *Lancet*, 389(10064), 77–90.
2. Chetty, R., Friedman, J. N., Hilger, N., Saez, E., Schanzenbach, D. W., & Yagan, D. (2011). How does your kindergarten classroom affect your earnings? Evidence from Project Star. *Quarterly Journal of Economics*, 126(4), 1593–1660.
3. Heckman, J., Pinto, R. & Savelyev, P. (2013). Understanding the mechanisms through which an influential early childhood program boosted adult outcomes. *American Economic Review*, 103 (6), 2052-86.
4. Holla, Alaka, Magdalena Bendini, Lelys DinarteIva, Iva Trako. (2021). Is Investment in Preprimary Education Too Low? Lessons from (Quasi) Experimental Evidence across Countries. Washington, DC: World Bank Policy Research Working Paper; No. 9723.
5. Nores, M., & Barnett, W. S. (2010). Benefits of early childhood interventions across the world: (Under) Investing in the very young. *Economics of Education Review, 29(2), 271–282.*
6. Berlinski, S., & Schady, N. (2015). The Early Years: Child Well-being and the Role of Public Policy. MacMillan Press, New York.
7. Engle, P. L., Fernald, L. C., Alderman, H., Behrman, J., O'Gara, C., Yousafzai, A., Cabral de Mello, M., Hidrobo, M., Ulkuer, N., Ertem, I., Iltus, S., & the Global Child Development Steering Group. (2011). Strategies for reducing inequalities and improving developmental outcomes for young children in low-income and middle-income countries. *Lancet,* 378(9799), 1339–1353.
8. OECD. (2006). Starting Strong II: Early Childhood Education and Care. OECD Publishing, Paris.
9. Sustainable Development Goals, 2030. Retrieved from: https://www.undp.org/sustainable-development-goals

10. UNESCO. Institute for Statistics. http://uis.unesco.org/en/files/who-pays-what-education-national-revealed-through-accounts-2016-en-pdf

11. Gil, J.D., Ewerling, F., Ferreira, L. Z., & Barros, A.J. (2020). Early childhood suspected

developmental delay in 63 low- and middle-income countries: Large within- and between-country inequalities documented using national health surveys. *Journal of global health*,10(1), 010427.

12. Su, Y. & Rao, N. (2020). Relationship between family's socioeconomic status and parental investments in early learning: Evidence from the China Family Panel Studies. *Peking University Education Review*, 18(3), 86-103. In Chinese
13. Lu, C., Cuartas J, Fink G, et al. (2020). Inequalities in early childhood care and development in low/middle-income countries: 2010–2018. *BMJ Global Health* 2020;5:e002314.
14. Ministry of Education of the People's Republic of China. http://www.moe.gov.cn/publicfiles/business/htmlfiles/moe/moe_2792/index.html
15. China Educational Finance Statistical Yearbook (2020).
16. Song, Y. (2019). Principal contradictions and reformation of public finance in early childhood education and care in China. *China Economics of Education Review*, 4(3), 19-48. In Chinese
17. Yuan, L., & Zhao, J. (2019). Issues in the fiscal policy for preschool education. *China Economics of Education Review*, 2019,4(03), 49-63. In Chinese
18. Ministry of Education of the People's Republic of China. (2020). Educational statistics in 2019. Retrieved from: http://www.moe.gov.cn/jyb_sjzl/moe_560/jytjsj_2019/qg/
19. Tian, Z., Zhang, X., & Yuan, L. (2011). The disparities of preschool educational resources for children with different household registration: A survey in Beijing. *Renmin University of China Education Journal*, (03), 165-180. In Chinese
20. Liu, Y., & Song, Y. (2013). Survey on the family expenditure for pre-school education of 3-to-6-year-old children in urban families. *Journal of Central China Normal University (Humanities and Social Sciences)*, 52(01), 155-160. In Chinese
21. Wagstaff, A., Glores G, Hsu J. et al. Progress on catastrophic health spending in 133 countries: a retrospective observational study. *Lancet Global Health* 2018;6:e169-79.
22. Xu, K., Evans, D. B., Kawabata, K., Zeramdini, R., Klavus, J., & Murray, C. J. (2003). Household catastrophic health expenditure: a multicountry analysis. *Lancet*, 362(9378), 111–117.
23. Husereau D, Drummond M, Petrou S, Carswell C, Moher D, Greenberg D, Augustovski F, Briggs AH, Mauskopf J, Loder E. Consolidated Health Economic Evaluation Reporting Standards (CHEERS) statement.
24. [dataset] Survey and Research Center for China Household Finance. https://chfs.swufe.edu.cn/
25. Office of Child Care (OCC), Administration for Children and Families (ACF), Department of Health and Human Services (HHS). (2016). Child Care and Development Fund (CCDF): Final rule. Retrieved from https://www.federalregister.gov/documents/2016/09/30/2016-22986/child-careand-development-fund-ccdf-program
26. Cumming, G., & Finch, S. (2005). Inference by eye: Confidence intervals and how to read pictures of data. *American Psychologist*, 60(2),170-180.
27. Wang, J., Yuan, C.,& Ma, N. (2022). Government competition, factor flow and regional collapse. *Journal of Xi'an Jiaotong University (Social Science)*, 42(2), 40-52. In Chinese

Confidential: For Review Only

28. Shang, W., Lu, S., & Li, T. (2020). The collapse of central regions in the development of compulsory education in China: Representation, influencing factors, and policy rationales. *Peking University Education Review*, 18(2), 172-192. In Chinese
29. Lei, W., Qian, J.,& Ma, H. (2014). Studies on compulsory education investment collapse in central China. *Economics and Education*, 2014(6), 1-9. In Chinese
30. Li, Z., Gong, X., & Qian, J. (2018). Studies on preschool investment sinking in central China. *China Economics of Education Review*, 3(4), 78-90. In Chinese

Confidential: For Review Only

Table 1. Summary statistics of variables used in the analysis

Variable	Observation	Mean	SD	Min	Max
Child-level variables					
PPE expenditure per child	3,371	8,220	10,088	0.000	204,000
Female	3,371	0.470	0.499	0.000	1.000
Private kindergarten	3,371	0.560	0.496	0.000	1.000
Household-level variables					
PPE expenditure per household (CNY)	3,111	8,907	10,824	0.000	204,000
% of household expenditure on PPE	3,111	0.094	0.096	0.000	0.887
HBPPE (7%)	3,111	0.495	0.500	0.000	1.000
HBPPE (10%)	3,111	0.342	0.475	0.000	1.000
HBPPE (20%)	3,111	0.098	0.297	0.000	1.000
Number of children undertaking PPE	3,111	1.084	0.294	1.000	4.000
Children in private kindergarten	3,091	0.557	0.497	0.000	1.000
Household size	3,111	5.131	1.576	2.000	15.000
Per capita household net wealth (CNY)	3,111	241,004	562,588	-1,974,739	17,020,206
Household net wealth quintile	3,111				
Quintile 1	3,111	0.210	0.407	0.000	1.000
Quintile 2	3,111	0.252	0.434	0.000	1.000
Quintile 3	3,111	0.232	0.422	0.000	1.000
Quintile 4	3,111	0.187	0.390	0.000	1.000
Quintile 5	3,111	0.119	0.324	0.000	1.000
Maternal schooling					
Primary school and below	3,111	0.136	0.343	0.000	1.000
Middle school	3,111	0.436	0.496	0.000	1.000
High school	3,111	0.180	0.384	0.000	1.000
College or above	3,111	0.248	0.432	0.000	1.000
Rural residential area	3,111	0.352	0.478	0.000	1.000

Notes: The number of children undertaking PPE indicates the number of children who attended kindergarten in a household. Household size is defined as the total number of household members in a household. A household unit is defined as all members of a housing unit related by blood, marriage, or some other legal arrangement; or two or

more persons use incomes to make joint expenditures; or a single person living with others but is financially independent. Thus a household may include grandparents, especially in rural area. When analyzing households that have children in private kindergarten, we excluded the observations of 20 households that had children attending both public and private kindergarten. CNY: Chinese Yuan.

Table 2. Average level of PPE spending per child across 29 provinces and weighted mean difference of the mean between the provincial level and the national level

Province	PPE spending at the provincial level	Weighted mean difference*
Beijing	23,348	246
Tianjin	19,487	135
Liaoning	13,417	189
Zhejiang	11,057	155
Shanghai	10,279	51
Neimenggu	10,095	50
Guangdong	9,309	162
Jiangsu	8,163	47
Hunan	8,030	34
Henan	7,942	41
Fujian	7,877	15
Shaanxi	7,740	11
Chongqing	7,062	-6
Heilongjiang	6,980	-10
Hubei	6,964	-16
Shandong	6,852	-35
Jilin	6,850	-10
Hainan	6,614	-5
Jiangxi	6,202	-38
Shanxi	6,090	-33
Yunnan	6,004	-47
Ningxia	5,496	-9
Sichuan	5,456	-113
Anhui	4,869	-113
Guizhou	4,674	-69
Guangxi	4,536	-100
Gansu	4,506	-54
Hebei	4,246	-168
Qinghai	4,070	-14

*: The measures took zero as the reference, with positive values indicating provincial means higher than the national average, and negative values indicating provincial means lower than the national average.

Confidential: For Review Only

Table 3. Multilevel logit regression results on factors associated with household status on HBPPE defined with various thresholds

	HBPPE_7% (Odds ratio)	HBPPE_10% (Odds ratio)	HBPPE_20% (Odds ratio)
Household net wealth quintile (ref. Quintile 1)			
Quintile 2	0.833*	0.756**	0.665**
Quintile 3	0.560***	0.584***	0.581***
Quintile 4	0.598***	0.697***	0.595**
Quintile 5	0.634***	0.512***	0.503***
Mother's education (ref. Primary school and below)			
Middle school	1.192	1.200	1.095
High school	1.004	1.127	1.146
College/above	1.208	1.391*	1.428
Number of children undertaking PPE	3.451***	3.375***	3.233***
Household having children in private kindergarten	1.973***	2.050***	1.880***
Household size	0.799***	0.766***	0.789***
Rural	0.820**	0.892	1.225
Constant	0.744	0.407***	0.061***
N	3091	3091	3091
ICC (Province)	0.018	0.029	0.048
ICC (County | Province)	0.110	0.108	0.187

Notes: * p<0.10, ** p<0.05, *** p<0.01. ICC refers to intraclass correlations; ICC (Province) denotes the intraclass correlations at the province level, and ICC (County | Province) denotes the intraclass correlations at the county and province level. When we define the HBPPE measure with the threshold of 7%, for example, we estimated that province random effects composed 1.8% of the total residual variance of the HBPPE measure, while county and province random effects composed 11.0% of the total residual variance of the HBPPE measure.

Confidential: For Review Only

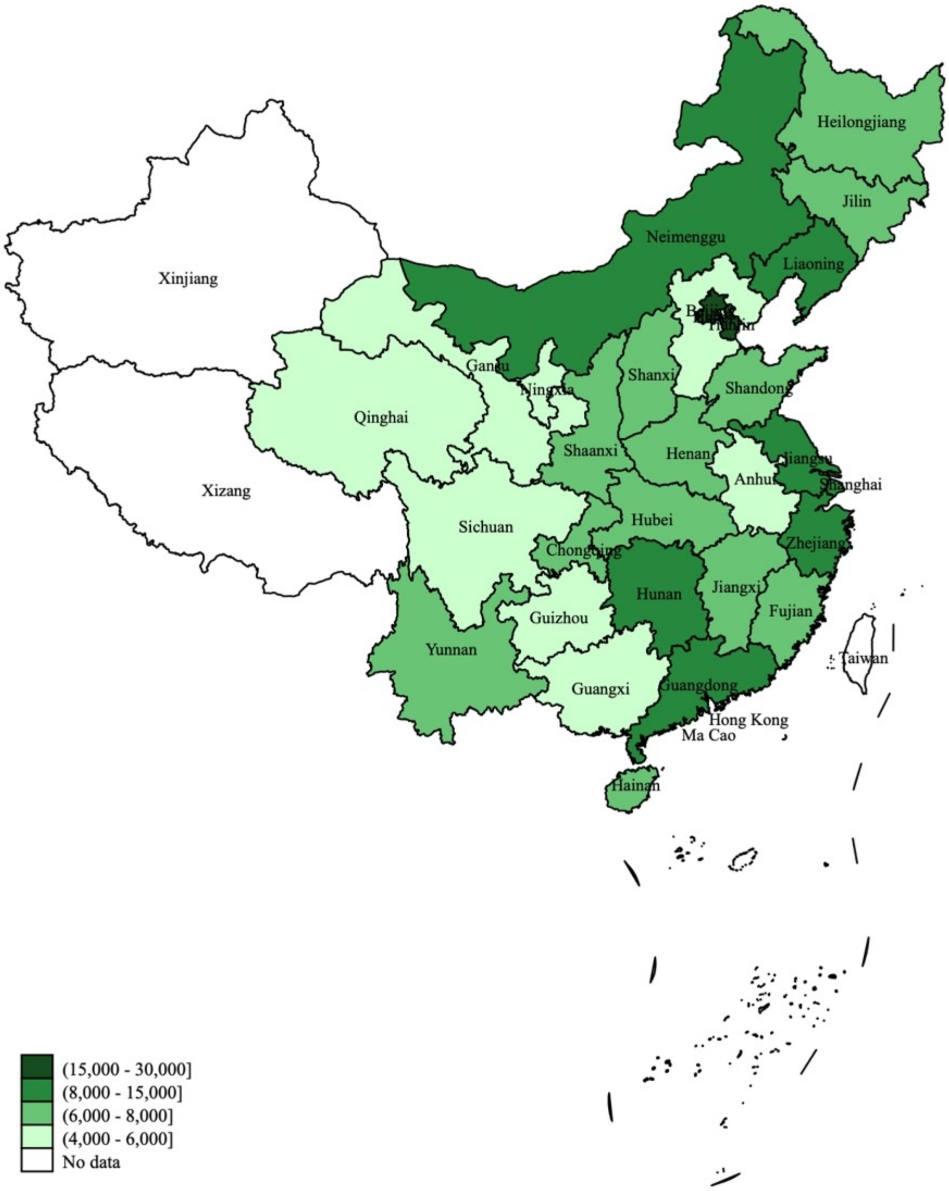

73x93mm (300 x 300 DPI)

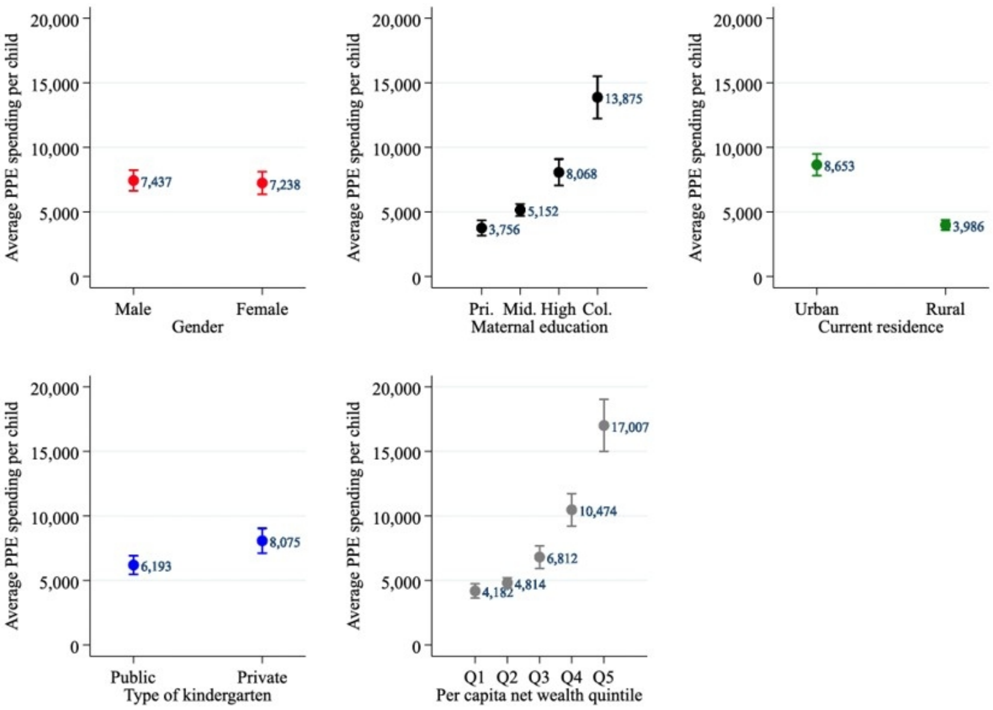

70x50mm (300 x 300 DPI)

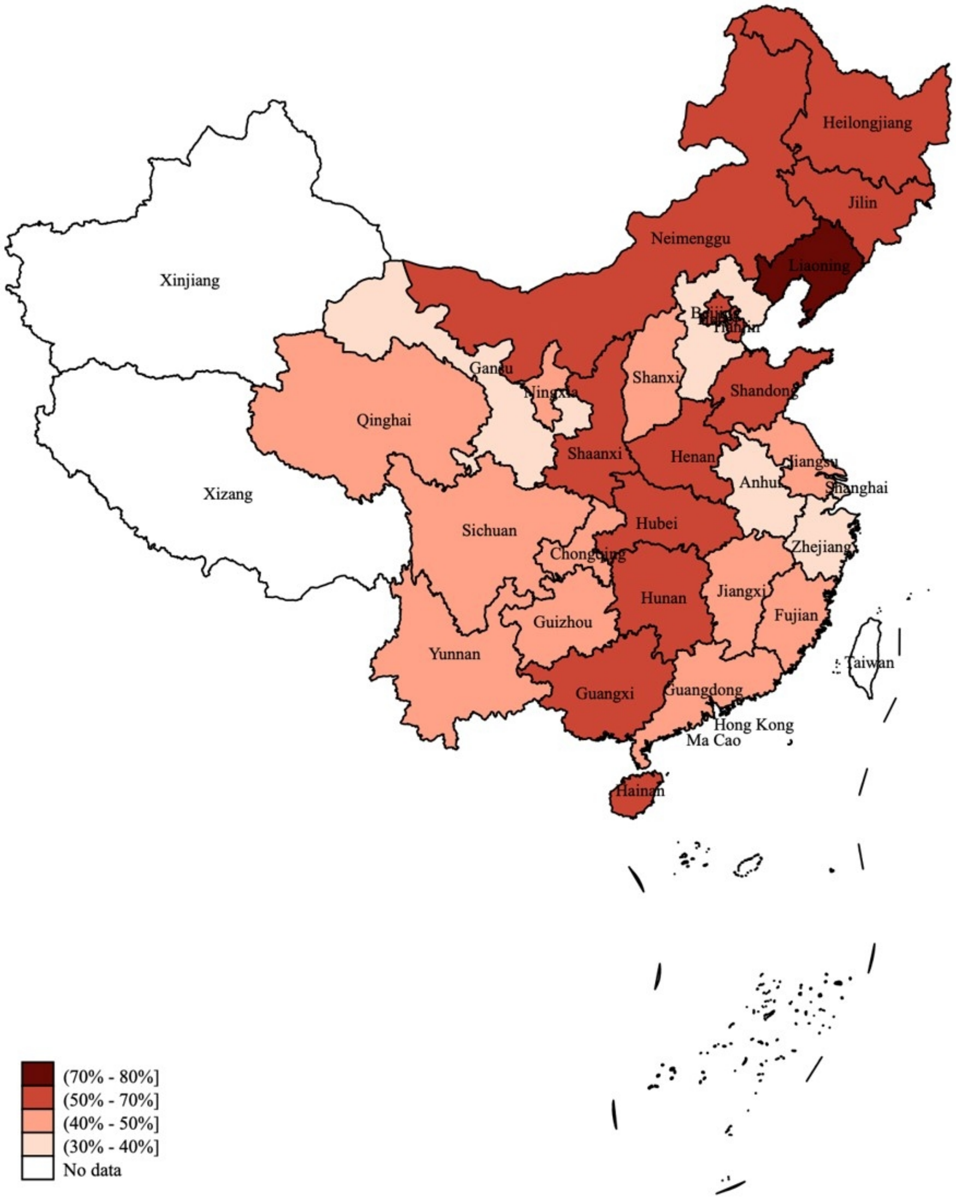

74x92mm (300 x 300 DPI)

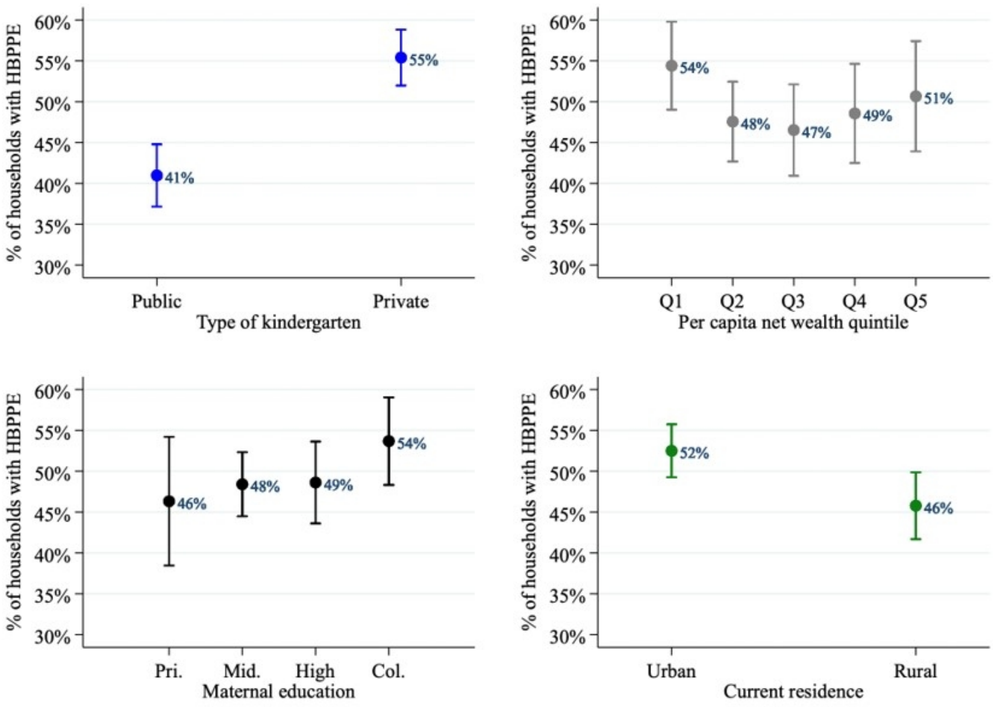

70x49mm (300 x 300 DPI)

Appendix

Chapter 1 Sampling Process of the China Household Finance Survey (CHFS)
Chapter 2 Constructing variables on socioecnomic charisteristics
Chapter 3 Testing the statistical significance of inequalities in PPE spending and HBPPE using Cumming and Finch's "rule of eye"
Chapter 4. Calculating provincial-level disparities in PPE spending and % of households with HBPPE
Chapter 5. Sensitivity tests using proportion of households spending on PPE as outcome variable and using continuous household net wealth as a predictor
Figure A1. Government education spending on preschool education
Figure A2. Preschool enrollment and gross enrollment rate
Figure A3. Geographical distribution of the sampled counties
Figure A4. Level and inequalities of PPE spending per child excluding out-school expenditure
Figure A5. Level of % of households with HBPPE (threshold=10%) across 29 provinces
Figure A6. Level of % of households with HBPPE (threshold=20%) across 29 provinces
Figure A7. Level and inequalities of % of households with HBPPE (thresehold=10%) by type of kindergarten, maternal education, household net wealth quintile, and current residential area
Figure A8. Level and inequalities of % of households with HBPPE (thresehold=20%) by type of kindergarten, maternal education, household net wealth quintile, and current residential area
Figure A9. Level and inequalities of % of households with HBPPE (threshold=7%) by excluding out-school spending from PPE spending
Figure A10. Level and inequalities of % of households with HBPPE (threshold=10%) by excluding out-school spending from PPE spending
Figure A11. Level and inequalities of % of households with HBPPE (threshold=20%) by excluding out-school spending from PPE spending
Figure A12. Level of % of household expenditure on PPE across 29 provinces
Figure A13. Level and inequalities of % of household expenditure on PPE by type of kindergarten, maternal education, household net wealth quintile, and current residential area
Figure A14. Level and inequalities of % of household expenditure on PPE by excluding out-school spending from PPE spending

Table A1. Significance testing of inequalities in PPE spending per child using Cumming and Finch's "rule of eye"
Table A2. Level of % of households with HBPPE (threshold=7%) across 29 provinces and weighted mean difference of the mean between the provincial level and the national level
Table A3. Level of % of households with HBPPE (threshold=10%) across 29 provinces and weighted mean difference of the mean between the provincial level and the national level
Table A4. Level of % of households with HBPPE (threshold=20%) across 29 provinces and weighted mean difference of the mean between the provincial level and the national level
Table A5. Significance testing of inequalities in HBPPE (threshold=7%) using Cumming and Finch's "rule of eye"

Confidential: For Review Only

Table A6. Significance testing of inequalities in HBPPE (threshold=10%) using Cumming and Finch's "rule of eye"

Table A7. Significance testing of inequalities in HBPPE (threshold=20%) using Cumming and Finch's "rule of eye"

Table A8. Level of % of household expenditure on PPE across 29 provinces and weighted mean difference of the mean between the provincial level and the national level

Table A9. Multilevel linear regression results on factors associated with logged % of household expenditure on PPE

Table A10. Beta regression results on factors associated with % of household expenditure on PPE

Table A11. Multilevel logit regression results on factors associated with household status on HBPPE by setting household net wealth as a continuous variable

Table A12. Multilevel linear regression and Beta regression results on factors associated with % of household expenditure on PPE by setting household net wealth as a continuous variable

Chapter 1 Sampling Process of the China Household Finance Survey (CHFS)

The CHFS survey employs a stratified three-stage probability proportion-to-size random sample design. The primary sampling units include 2,585 counties/provincial cities in China. Tibet, Xinjiang, Hong Kong, Macao, and Chinese Taipei are not included in the primary sampling stage. The second stage of sampling involves selecting residential committees/villages from the counties/cities. The last stage involves selecting households from the residential committees/villages. A household unit is defined as all members of a housing unit related by blood, marriage, or some other legal arrangement; or two or more persons use incomes to make joint expenditures, or a single person living with others but is financially independent.

The CHFS is nationally and provincially representative survey conducted by the Southwestern University of Finance and Economics in China.[1] The CHFS collects rich information on household assets, income, wealth, and consumption. It also collects socioeconomic and demographic information on the sampled households and their members, including residential areas, household members' ages, gender, education levels, marital status, etc. The CHFS data are publicly available and have been widely used in the fields of economics, sociology, and education. By the end of November 2021, more than 35,000 researchers and 3,000 research institutions had registered and applied for the dataset, and the related studies have been published in top peer-reviewed journals.[2] Since 2017, the CHFS survey collaborated with the China Institute for Educational Finance Research and included a module on household spending on young children's care and education. The module includes questions on young children's schooling status, types of schooling, and household spending on various items that are related to preprimary attendance.

The CHFS 2019 covers 29 provinces (excluding Tibet, Xinjiang, Inner Mongolia, Hong Kong, and Macau), 345 cities/districts/counties, 34,643 households, and 107,008 individuals. Our focus was on household spending related to attending kindergarten. In China, kindergarten is usually offered to children 3-6 years old, but children under age 3 and over age 6 may also attend. In this study, we focused on children attending kindergarten, including those under age 3 or over age 6

Confidential: For Review Only

who still attended kindergarten (18.2%). The 2019 data includes 4,085 young children aged 3-6 years old and 2,797 of these children attended kindergartens, yielding a 68.5% net enrollment rate.[3] The number of children attending kindergarten in all age groups is 3,415, yielding 83.6% of the gross enrollment rate, very close to the governmental estimate (83.4%).[4] Among households in this dataset, 42 (0.18%) didn't report spending information on PPE, and two reported PPE spending exceeding total household expenditures, both of which groups were excluded. The final sample includes 3,371 young children attending kindergartens, from 3,111 households (9% of the total sample), who reported available PPE expenditure data.

References

1. Gan, L., Yin, Z., Jia, N., Xu, S., & Ma, S. (2013). Data you need to know about China, Springer.
2. Survey and Research Center for China Household Finance, at https://chfs.swufe.edu.cn/
3. The net enrolment rate (NER) is the number of students of the age of preprimary education (age 3-6) that are enrolled in that level of education, expressed as a percentage of the total population in that age group.
4. The gross enrollment rate (GER) is the total enrollment in preprimary education, regardless of age, expressed as a percentage of the population in the official age group (age 3-6) corresponding to this level of education. The GER can exceed 100% because of early or late entry and/or grade repetition.

Chapter 2 Constructing variables on socioecnomic charisteristics

Household economic status. In this study, we used per capita household net wealth to measure household economic status. Household net wealth was derived by calculating the difference between a household's assets and its debts. Household total assets include financial assets (e.g., cash, deposit balances, social security, stock/bond value, etc.) and non-financial assets (e.g., agricultural capital, industrial capital, commercial capital, housing, vehicles, and durable goods). Total household debts include financial asset debts, agricultural debts, business debts, housing debts, vehicle debts, other non-financial asset debts, educational debts, credit card debts, medical-care debts, and other debts. In this study, we divided households into five quintiles according to their per capita net wealth. We examined the difference in % of households with HBPPE across the five wealth quintiles.

Maternal schooling. We divided maternal schooling into four categories: primary school or below, middle school, high school, and college or above. About 12% of households in the sample of the study did not report the mothers' education; we replaced the missing data with the predicted values of mother's education by regressing mothers' education on fathers' education (ordered logit regression coefficient = 1.217, $p < 0.001$; correlation coefficient = 0.746) or household heads' education (ordered logit regression coefficient = 1.393, $p < 0.001$; correlation coefficient = 0.632).

Residential area. Because the urban-rural divide is still one of the main structural problems facing the schooling system in China, we also examined the disparities between urban and rural residential areas.

Confidential: For Review Only

Type of kindergarten. Kindergartens in China can be categorized into public and private types, as discussed above. Households need to pay higher tuition fees to attend private kindergartens than public kindergartens. We assessed the disparities in the percentages of households with HBPPE by kindergarten type.

Chapter 3 Testing the statistical significance of inequalities in PPE spending and HBPPE using Cumming and Finch's "rule of eye"

To test whether inequalities in PPE spending or HBPPE significantly existed between the two sub-groups (e.g. male vs female), we compared the 95% confidence intervals (CIs) of the two estimates using Cumming and Finch's "rule of eye" for comparing two independent means.[1] We calculated three components that were required to implement the "rule of thumb": marginal errors, overlap of the 95% CIs of the two estimates, and proportion overlap. (1) Marginal error refers to the absolute difference between the mean and its lower or higher bound of a 95% CI. (2) An overlap of CIs refers to the difference between one estimate's higher bound and the other estimate's lower bound. (3) Proportion overlap is then calculated as the overlap of the 95% CIs between the two estimates divided by the average marginal errors between the two estimates.

Using Cumming and Finch's "rule of eye", we obtained proportion overlap using Equation PA1 or PA2 as follows, depending on the size of mean estimates for the two sub-groups. For example, when comparing the mean estimates for gender, if the mean estimate for male is lower than that for female, then

$$Proportion\ overlap = \frac{95\%\ CI\ upper\ bound_{male} - 95\%\ CI\ lower\ bound_{female}}{(Margin\ of\ error_{male} + Margin\ of\ error_{female})/2} \qquad \textbf{(PA1)}$$

Or, if the mean estimate for male is higher than that for female, then

$$Proportion\ overlap = \frac{95\%\ CI\ upper\ bound_{female} - 95\%\ CI\ lower\ bound_{male}}{(Margin\ of\ error_{male} + Margin\ of\ error_{female})/2} \qquad \textbf{(PA2)}$$

According to the "rule of eye", when sample sizes of two samples are both at least 10, and the margins of error between the two estimates do not differ by more than a factor of two, a proportion overlap less than 0.5 indicates a significant statistical relationship at the 0.05 level (i.e. $p<0.05$).

References

1. Cumming, G., & Finch, S. (2005). Inference by eye: Confidence intervals and how to read pictures of data. *American Psychologist*, 60(2),170-180.

Chapter 4. Calculating provincial-level disparities in PPE spending and % of households wth HBPPE

Considering the large variation in economic development and geographical situations across the provinces, we further assessed the provincial-level disparities in the level of PPE spending per child and % of households with HBPPE across the 29 provinces. The information will help the central government to identify the provinces with high percentages of households with HBPPE.

Confidential: For Review Only

Provincial-level inequalities were estimated with the weighted mean from the national mean, which was calculated as the values of the mean difference between each province and the national average, weighted by each province's population share (= $\frac{pop_i \times (r_i - r)}{pop}$), where *pop* is the national population size, pop_i is the population size of province *i*, *r* is the national mean, r_i is the mean of province *i*.). The measures took zero as the reference, with positive values indicating provincial means higher than the national average, and negative values indicating provincial means lower than the national average.

Confidential: For Review Only

Chapter 5. Sensitivity tests using proportion of households spending on PPE as the outcome variable and using continuous household net wealth as a predictor

We first calculated the provincial-level disparities in the % of household expenditure on PPE across the 29 provinces. On average, households in the Northeastern and middle areas spend higher % of household expenditure on PPE, with Shaanxi (15.4%) and Liaoning (14.8%) showing the highest level. Hebei (6.4%) and Gansu (6.0%) had the lowest level (**Appendix Figure A12 and Table A8)**. The pattern across the provinces is similar to the % of households with HBPPE.

In terms of socioeconomic inequalities (**Figure A13**), % of expenditure on PPE was higher among those households sending children to private kindergartens (10.5%) than to public kindergartens (8.1%). Statistically significant differences were not observed across urban and rural households, households with different levels of maternal education, households with different wealth quintiles. The results are consistent with the ones using dichotomous HBPPE. **Figure A14** shows results excluding out-school spending from PPE. The difference between private and public kindergartens increases. Meanwhile, the difference between the highest wealth quintile and the lowest one was statistically sigficant.

Tables A9 and A10 present the regression results using % of household total expenditure on PPE as outcome variable. We used both a multilevel linear regression and a beta regression for estimation. The results are consistent with the main regression results.

Tables A11 and A12 present the regression results using continuous household instead of net wealth quintiles as the predictor. We used a multilevel logit regression, multilevel linear regression and a beta regression for estimation. The results are consistent with the main regression results.

Figure A1. Government education spending on preschool education (Source: China Educational Finance Statistical Yearbook)

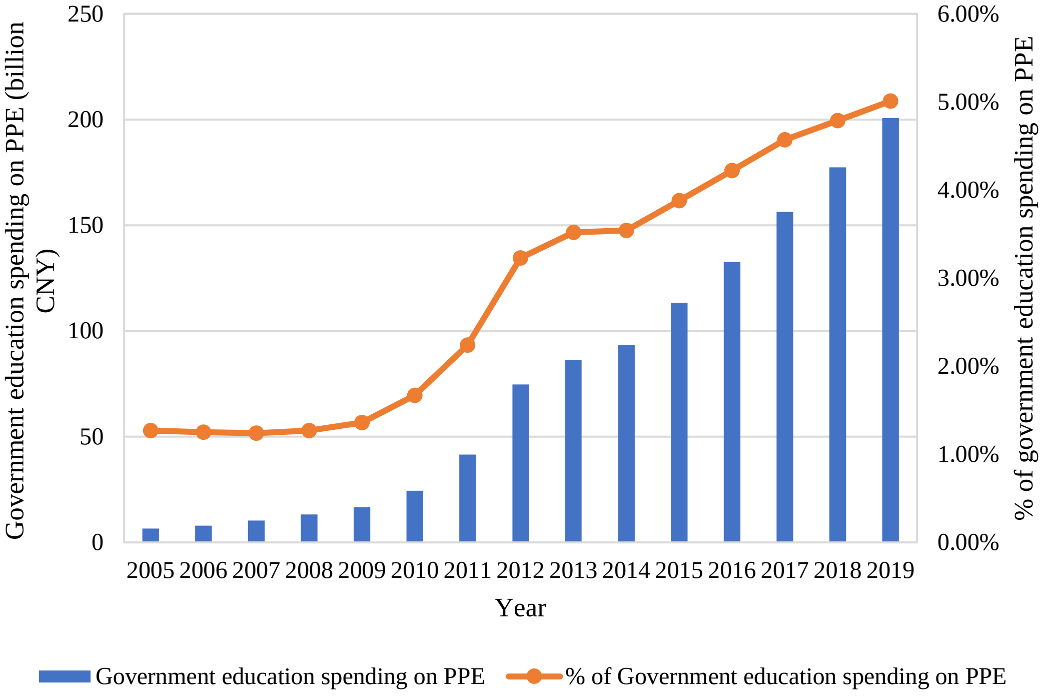

Figure A2. Preschool enrollment and gross enrollment rate (Source: China Education Statistical Yearbook)

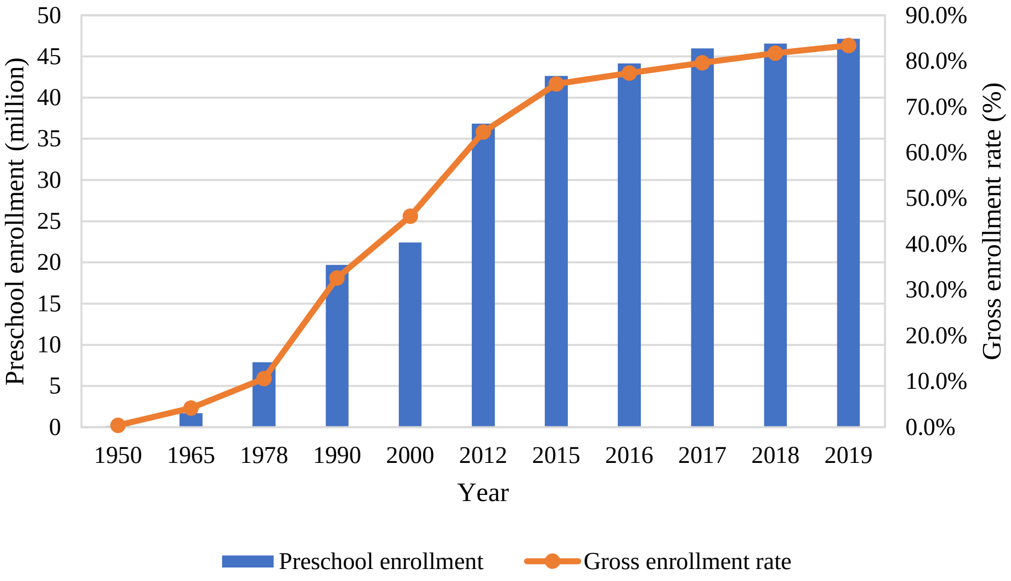

Figure A3. Geographical distribution of the sampled counties

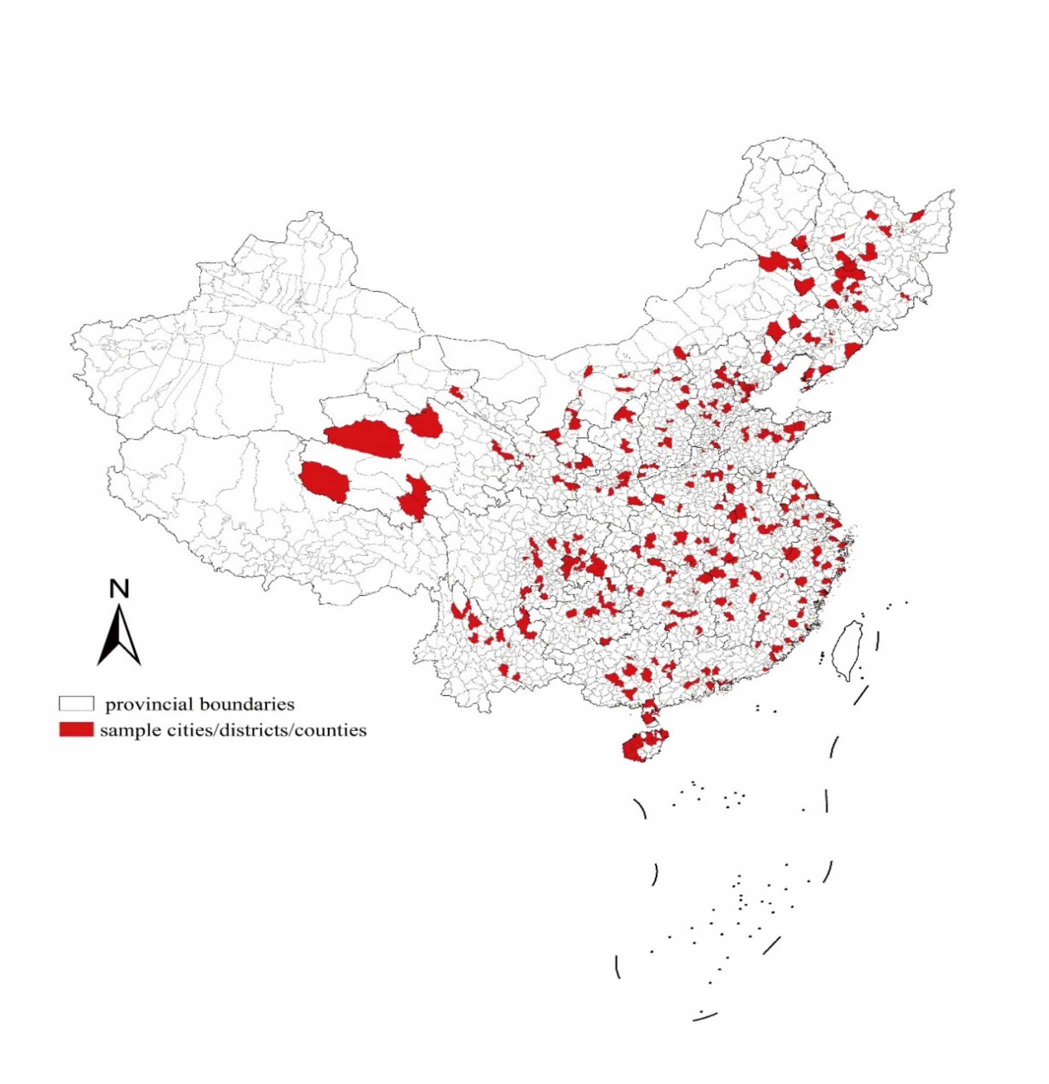

7

For peer review only

=

Confidential: For Review Only

Figure A4. Level and inequalities of PPE spending per child excluding out-school expenditure

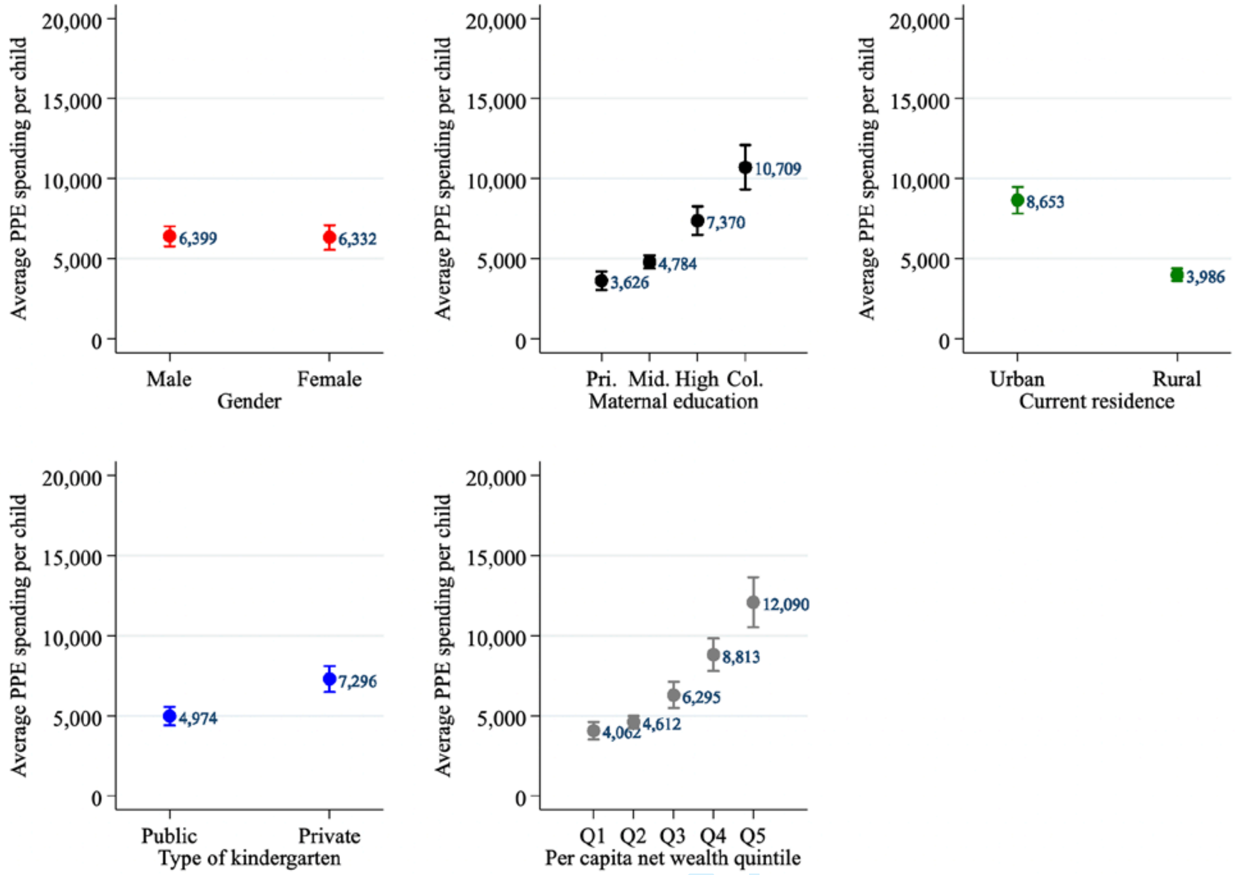

Notes: “Pri.”, “Mid.”, “High”, and “Col.” refers to maternal schooling at the primary and below, middle, high, and college and above levels, respectively.

Review Only

Figure A5. Level of % of households with HBPPE (threshold=10%) across 29 provinces

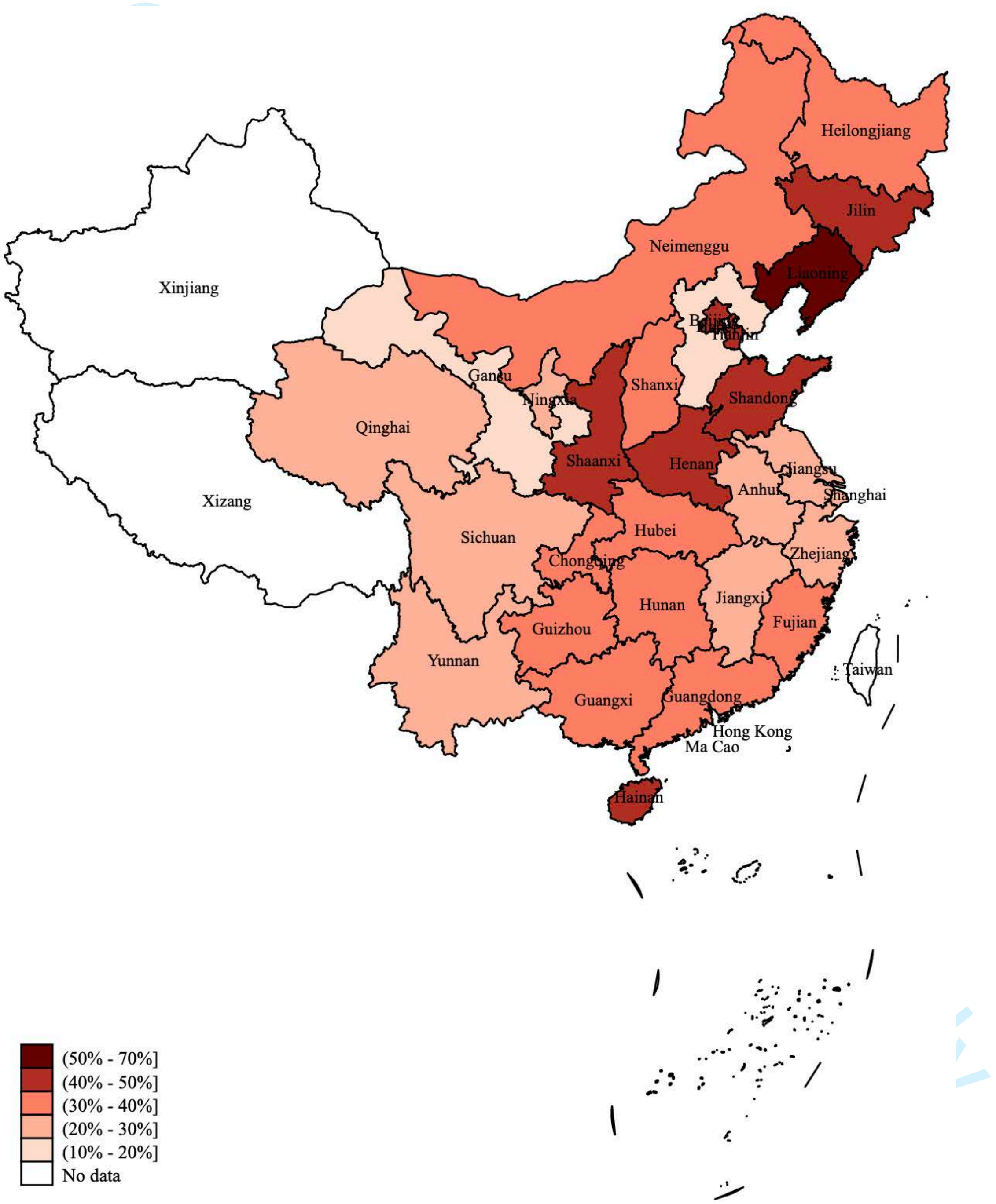

10

Figure A6. Level of % of households with HBPPE (threshold=20%) across 29 provinces

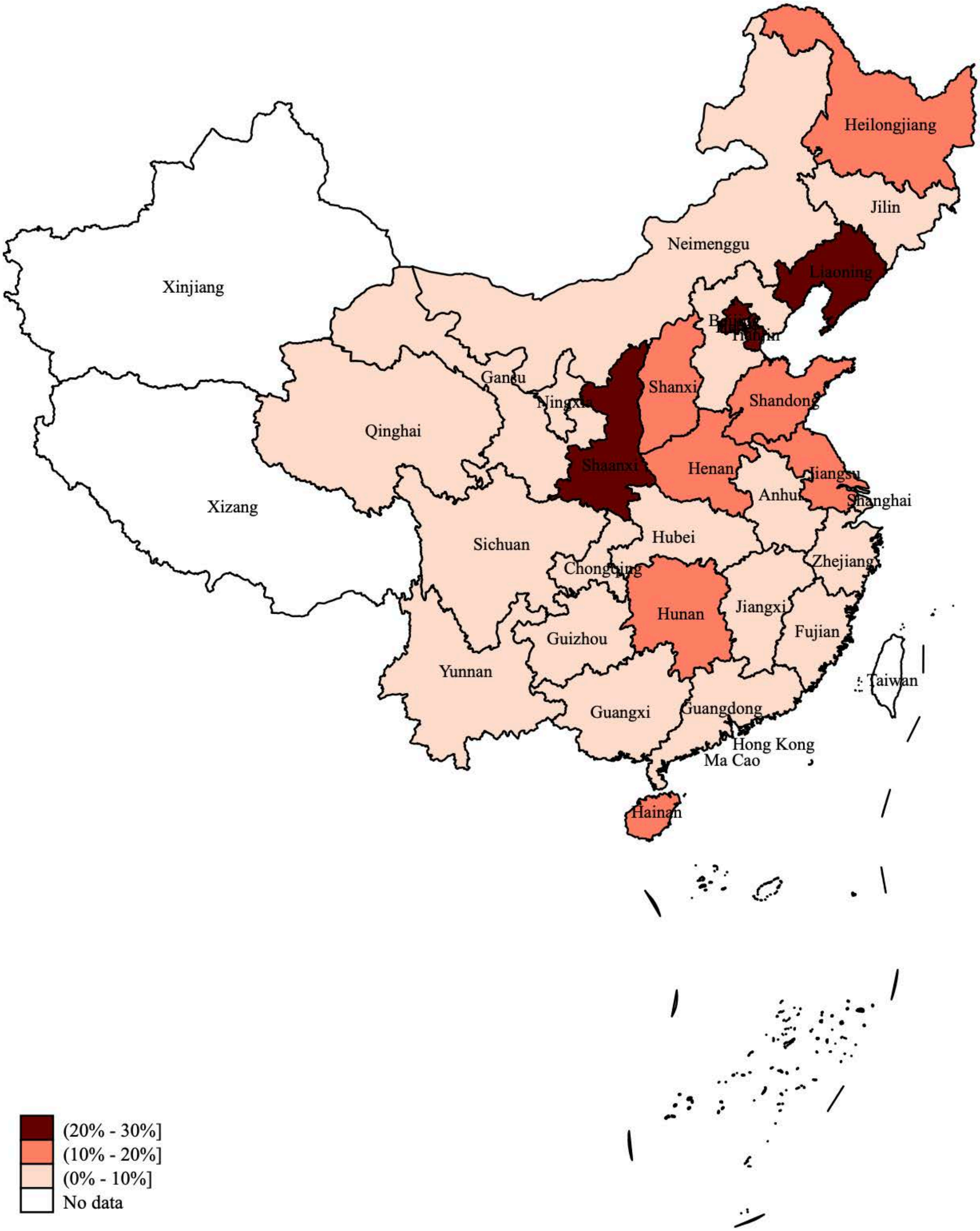

Figure A7. Level and inequalities of % of households with HBPPE (thresehold=10%) by type of kindergarten, maternal education, household net wealth quintile, and current residential area

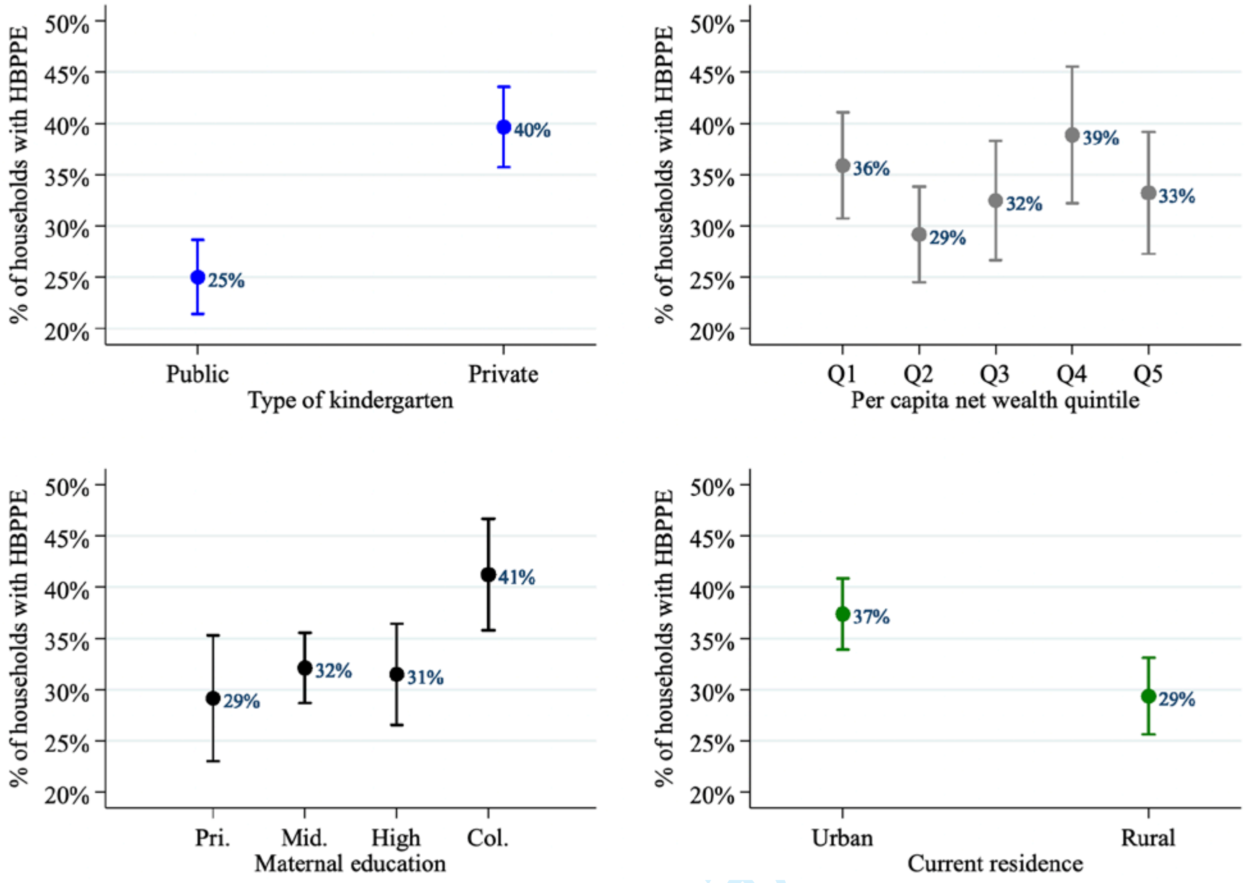

Notes: "Pri.", "Mid.", "High", and "Col." refers to maternal schooling at the primary and below, middle, high, and college and above levels, respectively.

Figure A8. Level and inequalities of % of households with HBPPE (thresehold=20%) by type of kindergarten, maternal education, household net wealth quintile, and current residential area

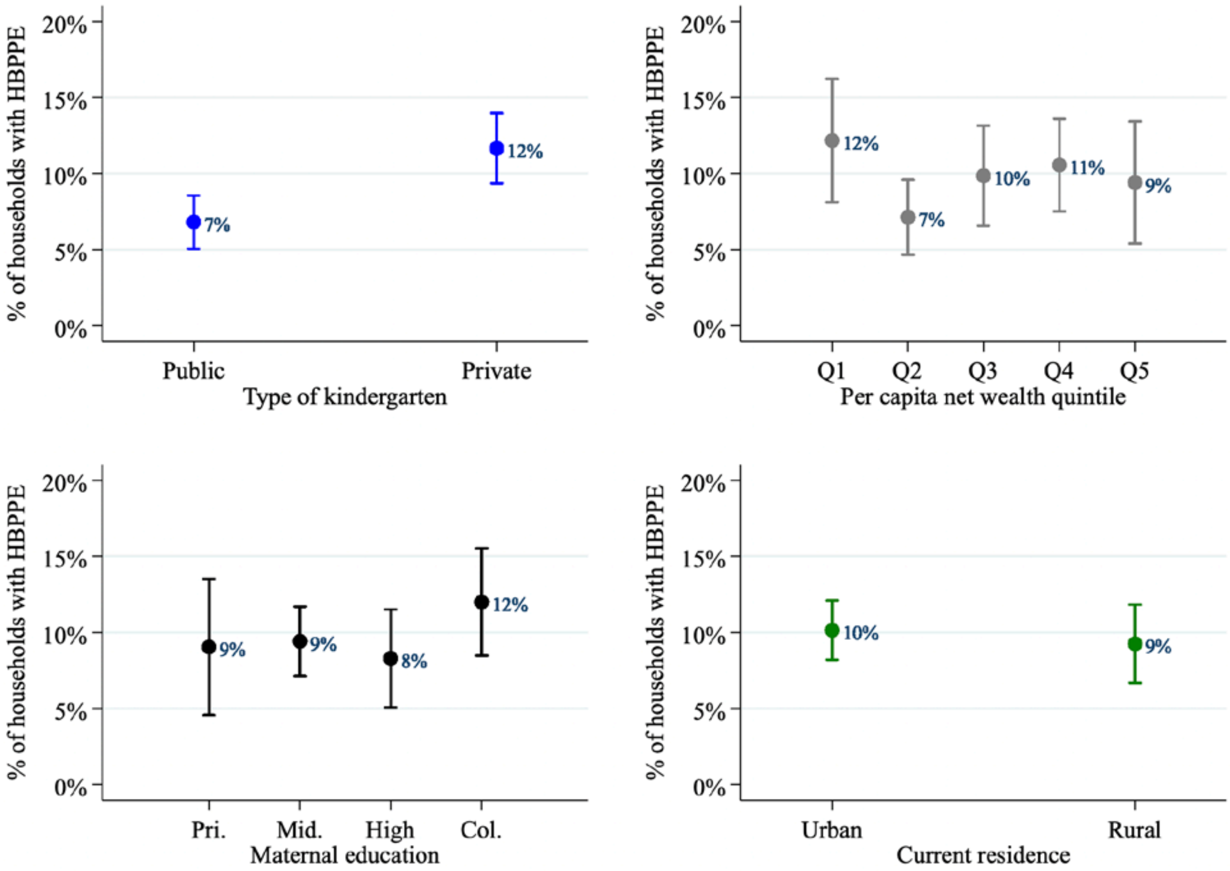

Notes: "Pri.", "Mid.", "High", and "Col." refers to maternal schooling at the primary and below, middle, high, and college and above levels, respectively.

Figure A9. Level and inequalities of % of households with HBPPE (thresehold=7%) by excluding out-school spending from PPE spending

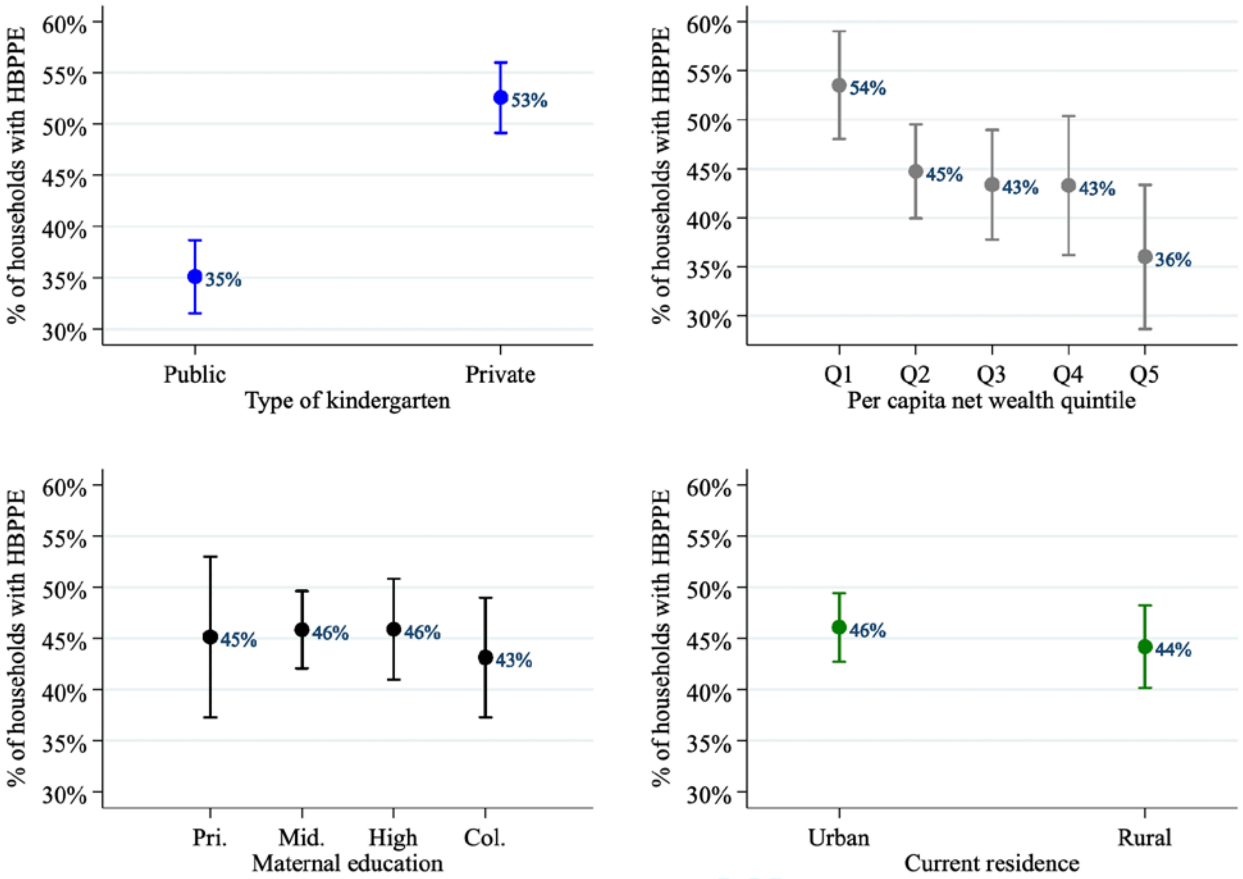

Notes: “Pri.”, “Mid.”, “High”, and “Col.” refers to maternal schooling at the primary and below, middle, high, and college and above levels, respectively.

Figure A10. Level and inequalities of % of households with HBPPE (thresehold=10%) by excluding out-school spending from PPE spending

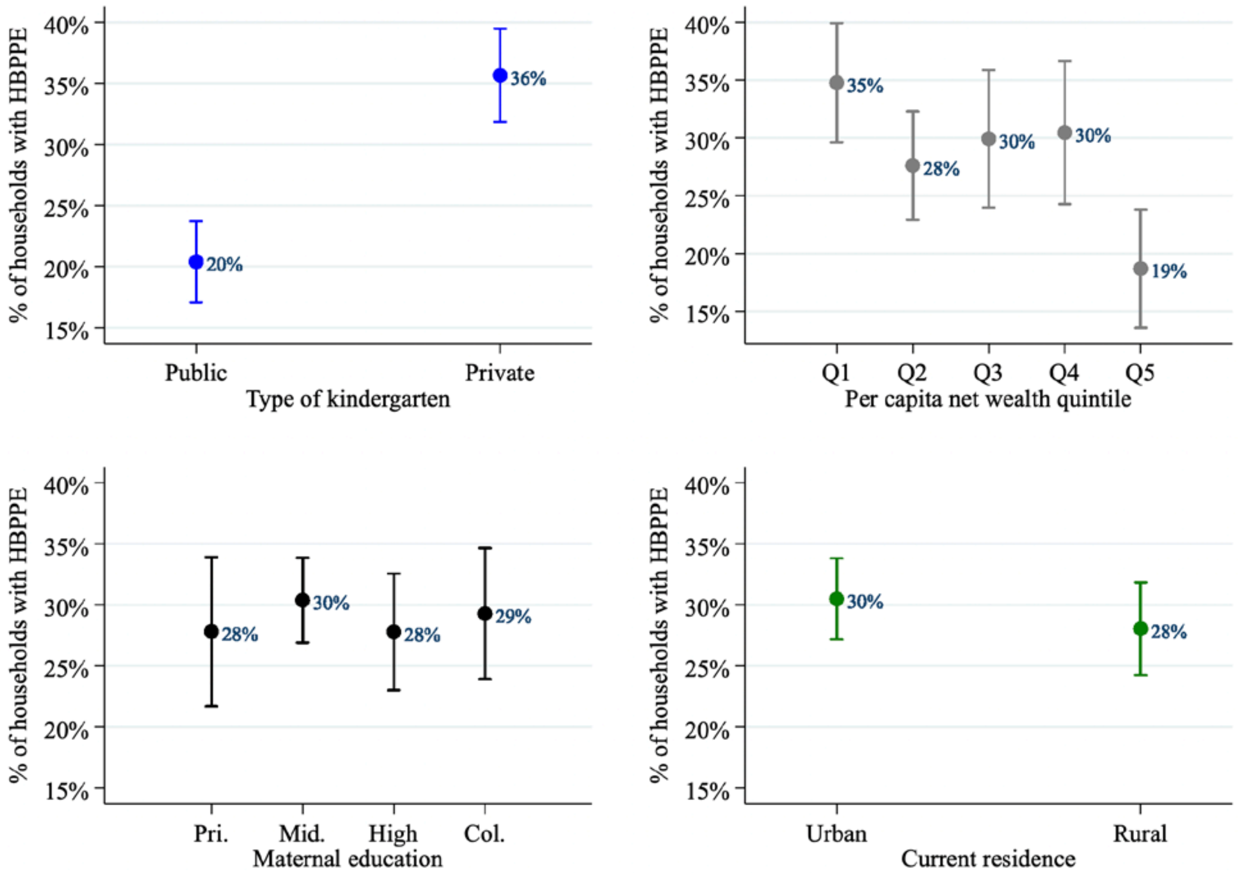

Notes: “Pri.”, “Mid.”, “High”, and “Col.” refers to maternal schooling at the primary and below, middle, high, and college and above levels, respectively.

review Only

Figure A11. Level and inequalities of % of households with HBPPE (thresehold=20%) by excluding out-school spending from PPE spending

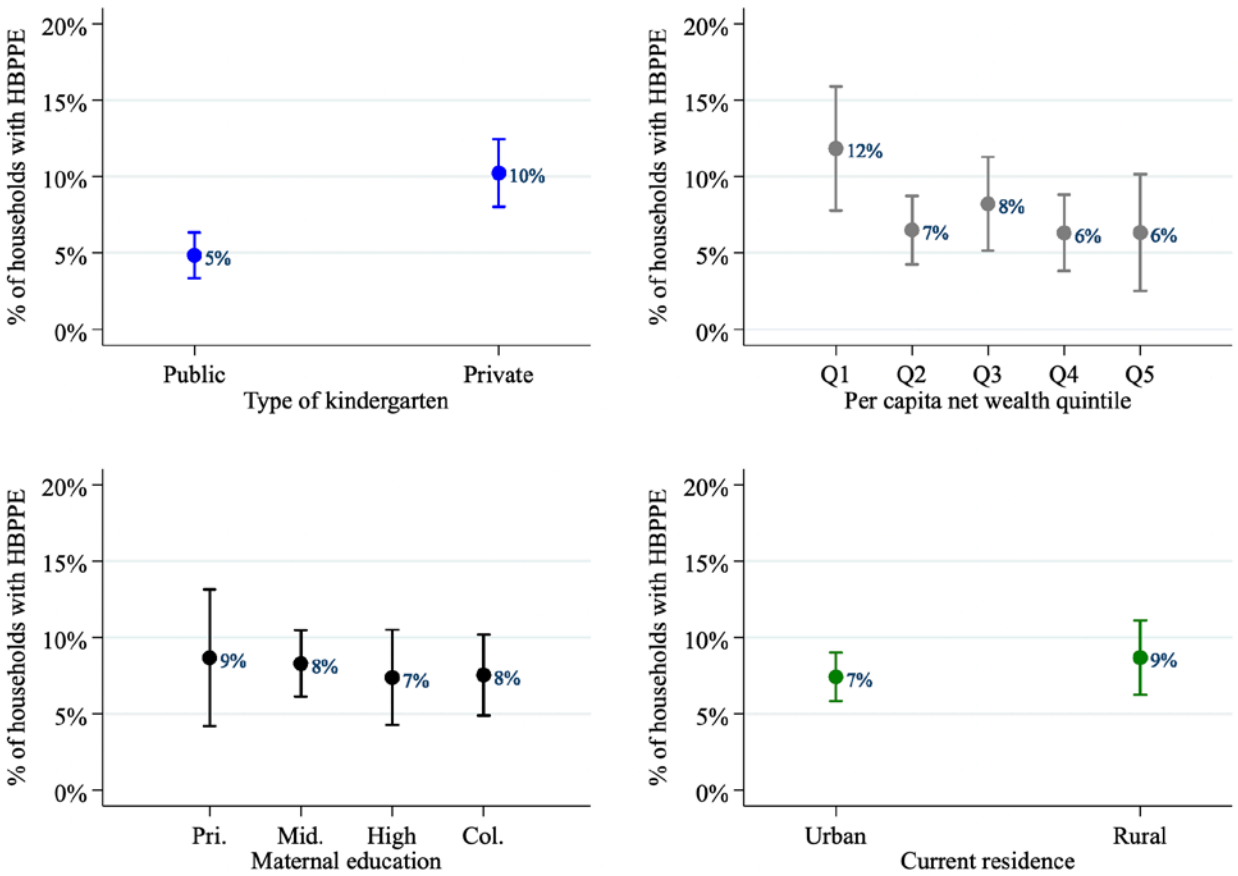

Notes: "Pri.", "Mid.", "High", and "Col." refers to maternal schooling at the primary and below, middle, high, and college and above levels, respectively.

Review Only

Figure A12. Level of % of household expenditure on PPE across 29 provinces

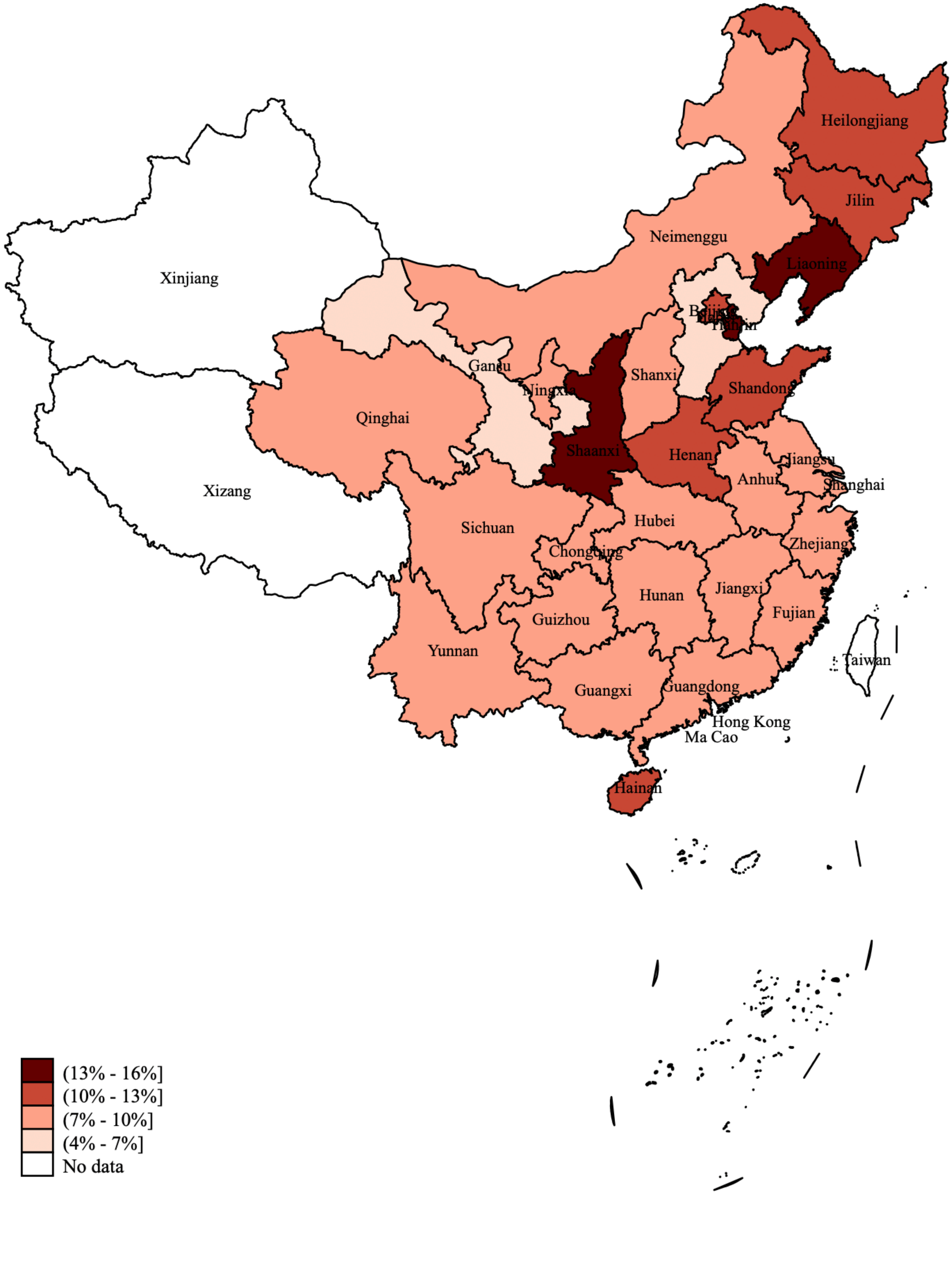

Figure A13. Level and inequalities of % of household expenditure on PPE by type of kindergarten, maternal education, household net wealth quintile, and current residential area

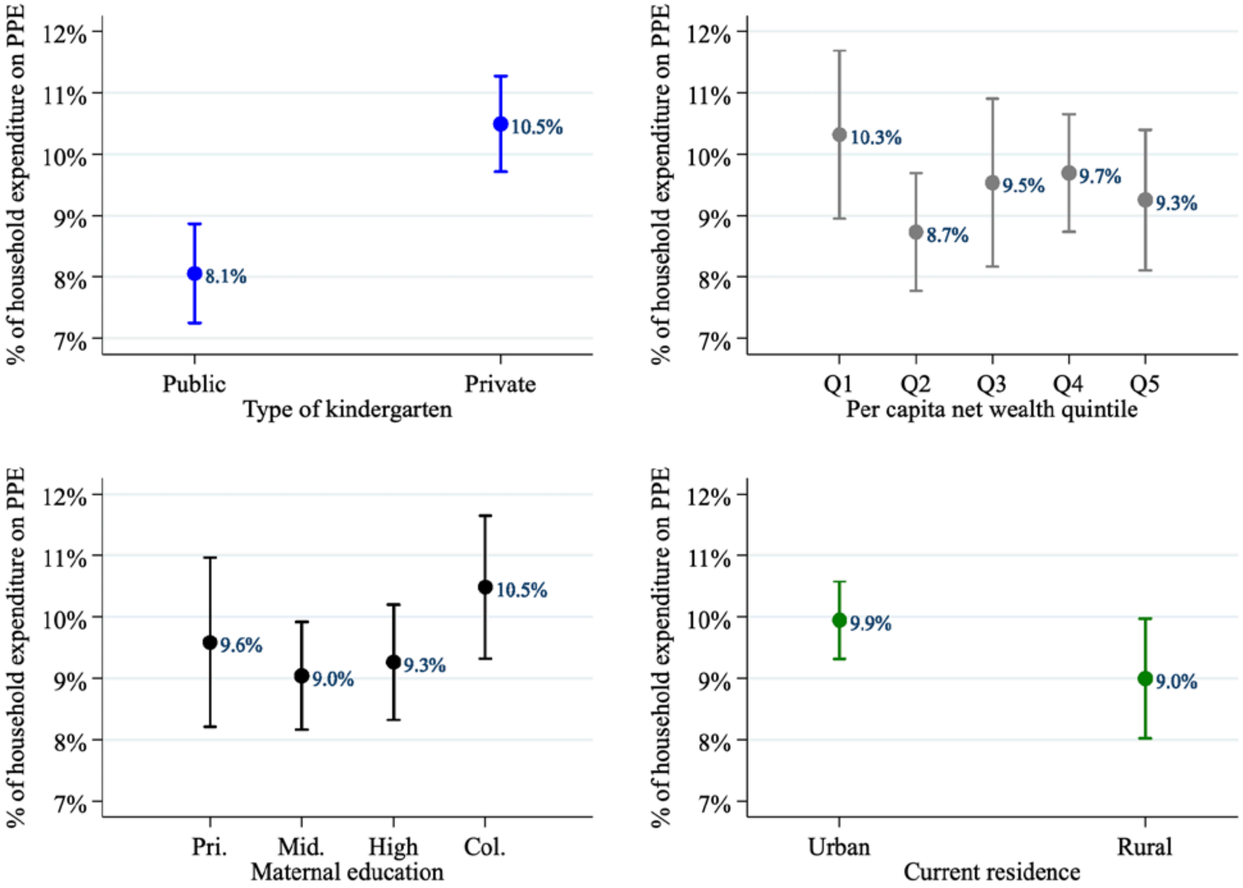

Notes: “Pri.”, “Mid.”, “High”, and “Col.” Refers to maternal schooling at the primary and below, middle, high, and college and above levels, respectively.

Figure A14. Level and inequalities of % of household expenditure on PPE by excluding out-school spending from PPE spending

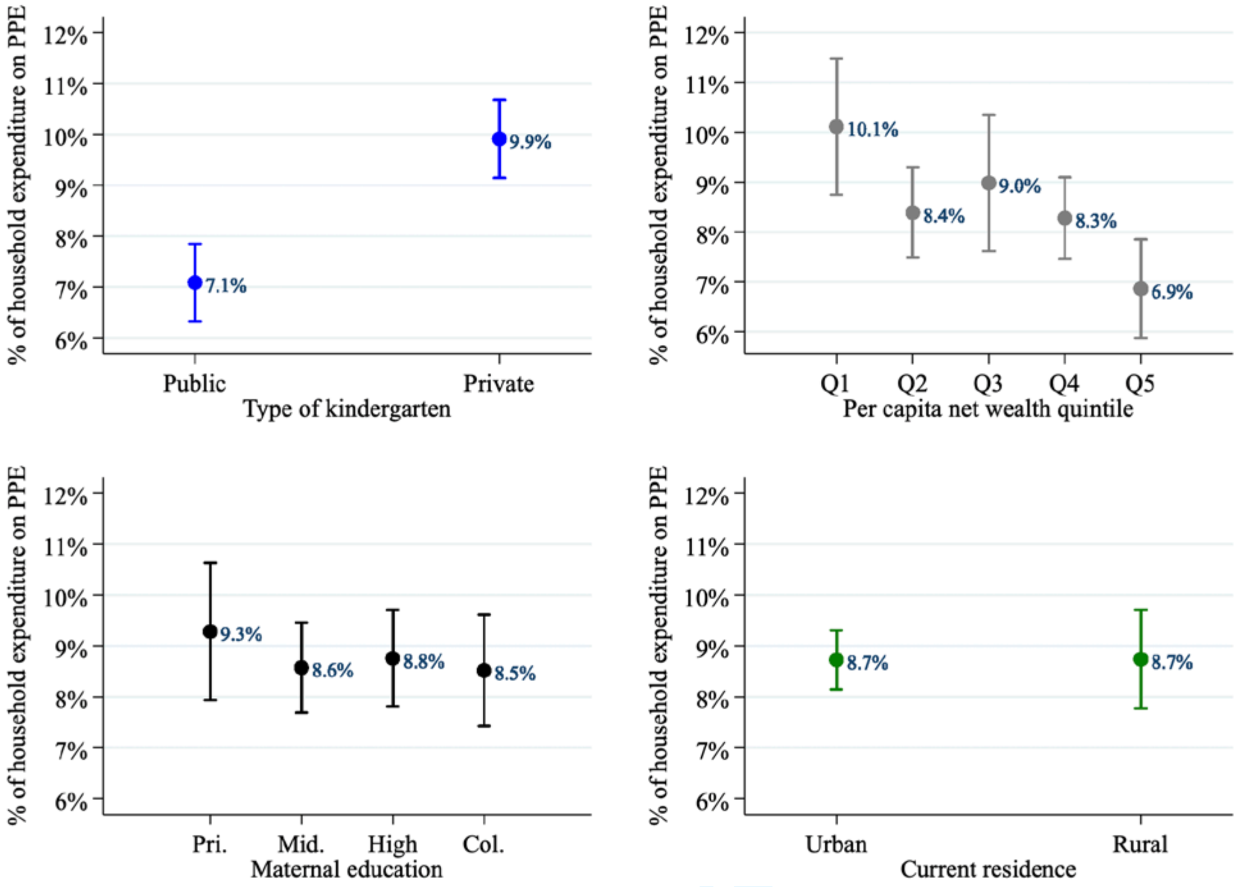

Notes: "Pri.", "Mid.", "High", and "Col." refers to maternal schooling at the primary and below, middle, high, and college and above levels, respectively.

For peer review Only

Confidential: For Review Only

Table A1. Significance testing of inequalities in PPE spending per child using Cumming and Finch's "rule of eye"

	Mean	Lower bound	Higher bound	Margins of error	Proportion overlap			
Gender					vs female			
Male	7437.056	6631.678	8242.434	805.378	1.763			
Female	7238.254	6369.469	8107.039	868.785				
Private school					vs private			
Public	6193.239	5475.135	6911.343	718.104	**-0.244**			
Private	8075.494	7116.165	9034.824	959.330				
Maternal education					vs middle	vs high	vs college	
Primary	3756.339	3163.960	4348.719	592.379	**-0.615**	**-3.341**	**-6.995**	
Middle	5138.580	4673.767	5603.393	464.813	.	**-1.940**	**-6.229**	
High	8068.083	7045.833	9090.333	1022.250	.	.	**-2.314**	
College	13824.870	12178.490	15471.250	1646.381				
Per capita net wealth quintile					vs Q2	vs Q3	vs Q4	vs Q5
Q1	4182.038	3628.280	4735.797	553.759	0.683	**-1.678**	**-4.956**	**-7.985**
Q2	4813.727	4408.062	5219.393	405.666	.	**-1.117**	**-4.816**	**-8.074**
Q3	6812.270	5935.722	7688.817	876.548	.	.	**-1.435**	**-5.051**
Q4	10473.690	9218.501	11728.880	1255.188	.	.	.	**-1.995**
Q5	17007.120	14991.910	19022.340	2015.218				
Current residence					vs rural			
Urban	10433.780	9434.861	11432.700	998.918	**-7.059**			
Rural	4126.709	3733.122	4520.297	393.588				

Notes: we used Cumming and Finch's "rule of eye" to test the statistical significance of inequalities in PPE spending between the two sub-groups (i.e. whether significant difference existed between the two mean estimates). Details for the analytical methods were presented in **Appendix Panel A**. Numbers in bold indicates a proportion overlap that is less than 0.5; that is a significant statistical relationship at the 0.05 level (p<0.05)

Confidential: For Review Only

Table A2. Level of % of households with HBPPE (threshold=7%) across 29 provinces and weighted mean difference of the mean between the provincial level and the national level

Province	Level of % of households with HBPPE at the provincial level	Weighted mean difference
Liaoning	76.9%	0.0086
Shaanxi	67.0%	0.0049
Beijing	64.5%	0.0023
Neimenggu	62.5%	0.0024
Henan	61.2%	0.0082
Hainan	60.0%	0.0007
Tianjin	59.9%	0.0012
Hubei	58.5%	0.0039
Heilongjiang	54.4%	0.0014
Hunan	53.8%	0.0022
Jilin	52.7%	0.0007
Guangxi	52.3%	0.0011
Shandong	51.5%	0.0016
Shanxi	49.3%	0.0000
Fujian	48.7%	-0.0002
Guangdong	47.7%	-0.0013
Jiangsu	46.6%	-0.0016
Sichuan	44.4%	-0.0029
Qinghai	43.9%	-0.0002
Jiangxi	42.2%	-0.0023
Chongqing	41.1%	-0.0018
Guizhou	40.9%	-0.0022
Yunnan	40.5%	-0.0030
Ningxia	40.5%	-0.0004
Anhui	39.9%	-0.0043
Shanghai	39.4%	-0.0017
Zhejiang	36.4%	-0.0054
Hebei	35.8%	-0.0073
Gansu	33.1%	-0.0031

*: The measures took zero as the reference, with positive values indicating provincial means higher than the national average, and negative values indicating provincial means lower than the national average.

Confidential: For Review Only

Table A3. Level of % of households with HBPPE (threshold=10%) across 29 provinces and weighted mean difference of the mean between the provincial level and the national level

Province	Level of % of households with HBPPE at the provincial level	Weighted mean difference
Liaoning	63.9%	0.0094
Tianjin	49.5%	0.0018
Beijing	49.1%	0.0024
Shaanxi	48.8%	0.0042
Jilin	47.0%	0.0026
Henan	46.1%	0.0086
Hainan	45.2%	0.0008
Shandong	42.5%	0.0064
Heilongjiang	39.2%	0.0015
Chongqing	36.5%	0.0007
Neimenggu	35.1%	0.0003
Hunan	34.2%	0.0003
Guangdong	33.4%	-0.0001
Fujian	32.0%	-0.0004
Hubei	31.9%	-0.0007
Guangxi	31.6%	-0.0007
Guizhou	30.9%	-0.0007
Shanxi	30.3%	-0.0009
Ningxia	29.7%	-0.0002
Jiangsu	29.3%	-0.0024
Yunnan	27.9%	-0.0020
Sichuan	27.6%	-0.0035
Anhui	25.9%	-0.0035
Shanghai	25.5%	-0.0014
Qinghai	23.6%	-0.0004
Zhejiang	23.1%	-0.0044
Jiangxi	22.7%	-0.0036
Hebei	19.7%	-0.0075
Gansu	19.0%	-0.0028

*: The measures took zero as the reference, with positive values indicating provincial means higher than the national average, and negative values indicating provincial means lower than the national average.

Confidential: For Review Only

Table A4. Level of % of households with HBPPE (threshold=20%) across 29 provinces and weighted mean difference of the mean between the provincial level and the national level

Province	Level of % of households with HBPPE at the provincial level	Weighted mean difference
Liaoning	27.8%	0.0056
Tianjin	23.6%	0.0015
Shaanxi	22.9%	0.0036
Beijing	20.1%	0.0016
Shandong	18.9%	0.0066
Hainan	17.3%	0.0005
Heilongjiang	16.9%	0.0019
Shanxi	12.8%	0.0008
Henan	12.6%	0.0020
Hunan	11.1%	0.0007
Jiangsu	10.2%	0.0003
Yunnan	10.0%	0.0001
Qinghai	9.9%	0.0000
Guangdong	9.3%	-0.0004
Guizhou	8.8%	-0.0002
Jilin	8.4%	-0.0003
Zhejiang	8.3%	-0.0006
Chongqing	6.8%	-0.0006
Neimenggu	6.7%	-0.0005
Hubei	6.4%	-0.0014
Shanghai	5.9%	-0.0007
Guangxi	5.2%	-0.0016
Gansu	5.1%	-0.0009
Ningxia	4.8%	-0.0002
Fujian	4.7%	-0.0014
Sichuan	4.5%	-0.0031
Jiangxi	4.4%	-0.0018
Hebei	4.0%	-0.0031
Anhui	2.9%	-0.0031

*: The measures took zero as the reference, with positive values indicating provincial means higher than the national average, and negative values indicating provincial means lower than the national average.

Confidential: For Review Only

Table A5. Significance testing of inequalities in HBPPE (threshold=7%) using Cumming and Finch's "rule of eye"

	Mean	Lower bound	Higher bound	Margins of error	Proportion overlap			
Private school					vs private			
Public	0.410	0.372	0.448	0.038	**-1.970**			
Private	0.554	0.520	0.588	0.034				
Maternal education					vs middle	vs high	vs college	
Primary	0.463	0.384	0.542	0.079	1.648	1.645	0.890	
Middle	0.484	0.445	0.523	0.039	.	1.952	0.864	
High	0.486	0.436	0.536	0.050	.	.	1.024	
College	0.537	0.483	0.590	0.054				
Per capita net wealth quintile					vs Q2	vs Q3	vs Q4	vs Q5
Q1	0.544	0.490	0.598	0.054	0.667	0.566	0.980	1.383
Q2	0.476	0.427	0.524	0.049	.	1.803	1.817	1.467
Q3	0.465	0.409	0.521	0.056	.	.	1.651	1.330
Q4	0.486	0.425	0.546	0.061	.	.	.	1.673
Q5	0.507	0.439	0.574	0.068				
Current residence					vs rural			
Urban	0.525	0.493	0.557	0.032	**0.168**			
Rural	0.458	0.417	0.499	0.041				

Notes: we used Cumming and Finch's "rule of eye" to test the statistical significance of inequalities in HBPPE between the two sub-groups (i.e. whether significant difference existed between the two mean estimates). Numbers in bold indicates a proportion overlap that is less than 0.5; that is a significant statistical relationship at the 0.05 level ($p<0.05$)

Confidential: For Review Only

Table A6. Significance testing of inequalities in HBPPE (threshold=10%) using Cumming and Finch's "rule of eye"

	Mean	Lower bound	Higher bound	Margins of error	Proportion overlap			
Private school					vs private			
Public	0.250	0.214	0.286	0.036	**-1.873**			
Private	0.396	0.357	0.436	0.039				
Maternal education					vs middle	vs high	vs college	
Primary	0.292	0.230	0.353	0.062	1.384	1.580	**-0.082**	
Middle	0.321	0.287	0.356	0.034	.	1.851	**-0.053**	
High	0.315	0.266	0.364	0.049	.	.	**0.122**	
College	0.412	0.358	0.467	0.054				
Per capita net wealth quintile					vs Q2	vs Q3	vs Q4	vs Q5
Q1	0.359	0.307	0.411	0.052	0.631	1.380	1.498	1.518
Q2	0.292	0.245	0.338	0.047	.	1.367	**0.288**	1.235
Q3	0.325	0.267	0.383	0.058	.	.	0.979	1.875
Q4	0.389	0.322	0.456	0.067	.	.	.	1.104
Q5	0.332	0.273	0.392	0.059				
Current residence					vs rural			
Urban	0.374	0.339	0.409	0.035	**-0.216**			
Rural	0.294	0.256	0.331	0.038				

Notes: Numbers in bold indicates a proportion overlap that is less than 0.5; that is a significant statistical relationship at the 0.05 level (p<0.05)

Confidential: For Review Only

Table A7. Significance testing of inequalities in HBPPE (threshold=20%) using Cumming and Finch's "rule of eye"

	Mean	Lower bound	Higher bound	Margins of error	Proportion overlap			
Private school					vs private			
Public	0.068	0.051	0.086	0.018	**-0.377**			
Private	0.116	0.093	0.140	0.023				
Maternal education					vs middle	vs high	vs college	
Primary	0.090	0.046	0.135	0.045	1.888	1.806	1.261	
Middle	0.094	0.071	0.117	0.023	.	1.591	1.112	
High	0.083	0.051	0.115	0.032	.	.	0.902	
College	0.120	0.085	0.155	0.035				
Per capita net wealth quintile					vs Q2	vs Q3	vs Q4	vs Q5
Q1	0.122	0.081	0.162	0.041	**0.459**	1.368	1.547	1.318
Q2	0.071	0.047	0.096	0.025	.	1.058	0.760	1.298
Q3	0.099	0.066	0.131	0.033	.	.	1.776	1.881
Q4	0.106	0.075	0.136	0.030	.	.	.	1.676
Q5	0.094	0.054	0.134	0.040				
Current residence					vs rural			
Urban	0.101	0.082	0.121	0.019	1.604			
Rural	0.092	0.067	0.118	0.026				

Notes: Numbers in bold indicates a proportion overlap that is less than 0.5; that is a significant statistical relationship at the 0.05 level (p<0.05)

Confidential: For Review Only

Table A8. Level of % of household expenditure on PPE across 29 provinces and weighted mean difference of the mean between the provincial level and the national level

Province	Level of % of household expenditure on PPE at the provincial level	Weighted mean difference
Shaanxi	15.4%	0.00163
Liaoning	14.8%	0.00166
Tianjin	13.1%	0.00040
Shandong	12.9%	0.00242
Beijing	12.6%	0.00048
Hainan	11.9%	0.00016
Henan	11.8%	0.00161
Heilongjiang	11.6%	0.00056
Jilin	10.5%	0.00019
Hunan	9.9%	0.00020
Shanxi	9.6%	0.00003
Hubei	9.3%	-0.00010
Guangdong	9.2%	-0.00024
Neimenggu	9.2%	-0.00006
Jiangsu	9.0%	-0.00026
Guizhou	8.8%	-0.00019
Chongqing	8.6%	-0.00020
Guangxi	8.6%	-0.00033
Fujian	8.3%	-0.00034
Qinghai	8.2%	-0.00006
Jiangxi	8.1%	-0.00046
Sichuan	8.0%	-0.00087
Zhejiang	8.0%	-0.00064
Yunnan	7.9%	-0.00055
Shanghai	7.7%	-0.00031
Ningxia	7.3%	-0.00011
Anhui	7.2%	-0.00106
Hebei	6.4%	-0.00169
Gansu	6.0%	-0.00066

Confidential: For Review Only

Table A9. Multilevel linear regression results on factors associated with logged % of household expenditure on PPE

	Logged % of household spending on PPE	Logged % of household spending on PPE by excluding out-school spending from PPE spending
Household net wealth quintile (ref. Quintile 1)		
Quintile 2	-0.134***	-0.128**
Quintile 3	-0.204***	-0.210***
Quintile 4	-0.212***	-0.255***
Quintile 5	-0.304***	-0.479***
Mother's education (ref. Primary school and below)		
Middle school	-0.015	-0.019
High school	0.020	-0.007
College/above	0.058	-0.055
Number of children undertaking PPE	0.499***	0.517***
Household having children in private kindergarten	0.331***	0.381***
Household size	-0.099***	-0.093***
Rural	-0.028	-0.011
Constant	-2.775***	-2.901***
N	2949	2935
ICC (Province)	0.033	0.036
ICC (County | Province)	0.150	0.166

Notes: * p<0.10, ** p<0.05, *** p<0.01. We took the natural logarithm of % of household expenditure on PPE to ensure the variable close to a normal distribution. Of note, because 142 out of 3,111 observations reported zero value for this variable, taking log generated 142 missing values. We adopted a multilevel linear model for estimation.

Table A10. Beta regression results on factors associated with % of household expenditure on PPE

	% of household spending on PPE (Odds ratio)	% of household spending on PPE by excluding out-school spending from PPE spending (Odds ratio)
Household net wealth quintile (ref. Quintile 1)		
Quintile 2	0.902**	0.904**
Quintile 3	0.842***	0.840***

Confidential: For Review Only

Quintile 4	0.862***	0.833***
Quintile 5	0.837***	0.723***
Mother's education (ref. Primary school and below)		
Middle school	1.059	1.054
High school	1.100**	1.082
College/above	1.151***	1.047
Number of children undertaking PPE	1.446***	1.473***
Household having children in private kindergarten	1.319***	1.384***
Household size	0.918***	0.925***
Rural	0.947*	0.961
N	2949	2935

Notes: * p<0.10, ** p<0.05, *** p<0.01. We adopted a beta regression for estimation, which is appropritate for modelling proportional outcome variables that are greater than 0 and less than 1. Of note, because 142 out of 3,111 observations reported zero value for this variable, performing a beta regression would exclude these observations with zero values.

Table A11. Multilevel logit regression results on factors associated with household status on HBPPE by setting household net wealth as a continuous variable

	HBPPE_7% (Odds ratio)	HBPPE_10% (Odds ratio)	HBPPE_20% (Odds ratio)
Log (Household net wealth)	0.870***	0.855***	0.873***
Mother's education (ref. Primary school and below)			
Middle school	1.084	1.012	0.733
High school	0.967	1.035	0.794
College/above	1.164	1.308	1.031
Number of children undertaking PPE	3.363***	3.433***	3.279***
Household having children in private kindergarten	1.962***	2.018***	1.900***
Household size	0.796***	0.757***	0.805***
Rural	0.824*	0.886	1.151
N	2995	2995	2995
ICC (Province)	0.022	0.033	0.062
ICC (County | Province)	0.117	0.112	0.204

Notes: * p<0.10, ** p<0.05, *** p<0.01. Of note, household net wealth was derived by calculating a household' assets minus its debts, leading to 97 household observations that had negative net wealth values. Taking logarithm of household net wealth generated 97 missing values for this variables.

Table A12. Multilevel linear regression and Beta regression results on factors associated with % of household expenditure on PPE by setting household net wealth as a continuous variable

	Logged % of household spending on PPE	% of household spending on PPE

Confidential: For Review Only

	(Multilevel linear regression)	(Beta regression)
Log (Household net wealth)	-0.065***	-0.041***
Mother's education (ref. Primary school and below)		
Middle school	-0.019	0.041
High school	0.030	0.093*
College/above	0.074	0.146***
Number of children undertaking PPE	0.497***	0.377***
Household having children in private kindergarten	0.336***	0.274***
Household size	-0.099***	-0.085***
Rural	-0.025	-0.048
N	2861	2861

Notes: * p<0.10, ** p<0.05, *** p<0.01.

Confidential: For Review Only
